# The prevalence of wholly attributable alcohol conditions in the United Kingdom hospital system: a systematic review, meta‐analysis and meta‐regression

**DOI:** 10.1111/add.14642

**Published:** 2019-07-03

**Authors:** Emmert Roberts, Rachel Morse, Sophie Epstein, Matthew Hotopf, David Leon, Colin Drummond

**Affiliations:** ^1^ National Addiction Centre and the Department of Psychological Medicine Institute of Psychiatry, Psychology and Neuroscience, King's College London and South London and the Maudsley NHS Foundation Trust London UK; ^2^ Brighton and Sussex Medical School University of Brighton and the University of Sussex Brighton UK; ^3^ NIHR Maudsley Biomedical Research Centre, South London and the Maudsley NHS Foundation Trust; Department of Child and Adolescent Psychiatry Institute of Psychiatry, Psychology and Neuroscience, King's College London London UK; ^4^ Department of Psychological Medicine Institute of Psychiatry, Psychology and Neuroscience, King's College London and South London and the Maudsley NHS Foundation Trust London UK; ^5^ Department of Non‐communicable Disease Epidemiology London School of Hygiene and Tropical Medicine London UK

**Keywords:** Alcohol, in‐patient, meta‐analysis, meta‐regression, prevalence, United Kingdom

## Abstract

**Background and Aims:**

The prevalence of alcohol‐related conditions is often reported as higher in hospital in‐patients compared with the general population. However, formal prevalence estimates are commonly derived from small studies which report highly varied results. This systematic review and meta‐analysis, within the UK hospital system, aimed to estimate the pooled prevalence of the 26 ICD‐10 conditions that are wholly attributable to alcohol in in‐patient settings.

**Methods:**

We searched Medline, Embase, PsychINFO and CENTRAL from database inception until 1 May 2018. We included studies of any design that reported the prevalence of one of 26 wholly attributable alcohol conditions defined by the ICD‐10. Studies were required to be conducted in one or more of the constituent nations of the United Kingdom and in an in‐patient setting (general wards, intensive care units, accident and emergency departments or mental health in‐patient units). Estimates were pooled using random‐effects meta‐analysis, and meta‐regression tested study and patient factors contributing to variation. Quality was assessed using the Grading of Recommendations Assessment Development and Evaluation (GRADE) framework.

**Results:**

A total of 124 studies were included, reporting on a total of 1 657 614 patients. The majority of studies reported on harmful use of alcohol and alcohol dependence, for which the pooled prevalence was 19.76% [95% confidence interval (CI) = 15.61–24.26%] and 10.25% (95% CI = 7.06–13.96%), respectively. Mean patient age and type of in‐patient setting were identified as the main sources of variation in prevalence estimates, but not date of data collection. Both estimates were deemed very low quality according to GRADE.

**Conclusions:**

An estimated one in five patients in the UK hospital system use alcohol harmfully, and one in 10 are alcohol‐dependent.

## INTRODUCTION

The prevalence of alcohol‐related conditions is often reported as higher in hospital in‐patients compared to the general population 1; however, formal prevalence estimates are frequently derived from small studies which report highly variable results. Such estimates are also often restricted to subsets of patients with particular diseases 2, making it difficult to interpret the true overall prevalence of these conditions in the in‐patient population.

Alcohol‐related conditions are estimated to cost the UK National Health Service (NHS) approximately 3.5 billion pounds per year 3. In the current epoch of stretched financial resources, accurate quantification of their prevalence in in‐patient settings is important to ensure appropriate resource allocation. Alcohol is a potential causative factor for a plethora of conditions 4, and without dedicated in‐hospital screening many alcohol‐related conditions may be missed, and not receive appropriate treatment 5. Accurate prevalence estimates are therefore vital to inform patients, clinicians, commissioners and policymakers as to the scale of the problem, and are currently particularly pertinent given the UK government's development of a new alcohol strategy and the NHS 10‐year plan which includes funding allocations to combat alcohol‐related conditions 6.

The current evidence base is confused and contradictory, with a large range of differing prevalence estimates of alcohol‐related conditions reported in the literature 7, 8. Previous attempts to synthesize data through systematic review have made no attempt to pool data, have been narratively reported and have either focused on general medical in‐patients or on subpopulations of patients with particular conditions 9, 10. Reviews have also been narrowly focused, often reporting on a singular alcohol‐related condition 11. As such, no robust pooled estimates in the UK hospital system are currently available in the literature.

**Figure 1:**
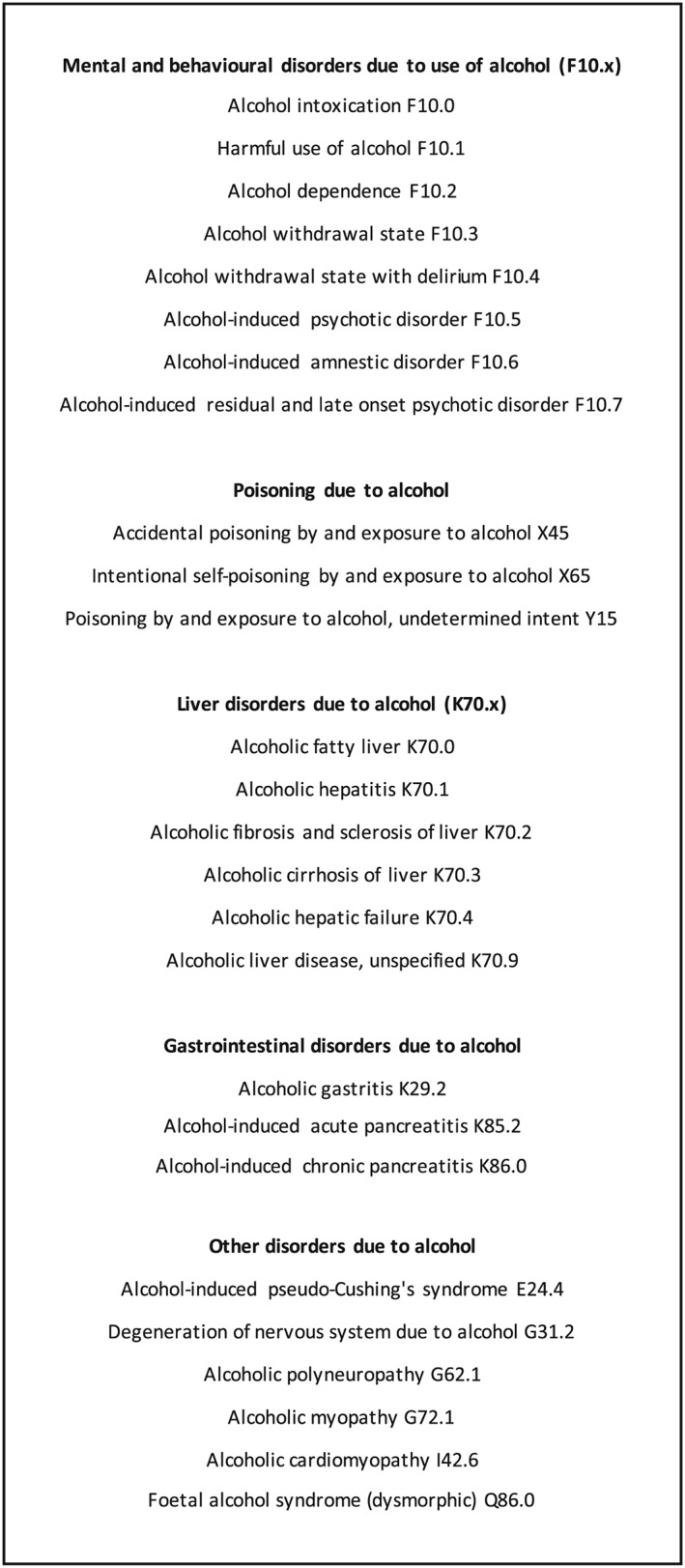
The 26 wholly attributable alcohol diagnoses as coded in the International Classification of Diseases Diagnostic Criteria for Research Volume 10 (ICD‐10 DCR) 12

The UK uses the International Classification of Diseases—Diagnostic Criteria for Research volume 10 (ICD‐10 DCR) as the gold standard to code alcohol‐related diagnoses 12. This defines a diagnostic classification system of 26 unique conditions which are wholly attributable to alcohol. A list of these, along with their diagnostic codes, is shown in Fig. 1. We aimed to use meta‐analysis to generate pooled prevalence estimates of these 26 conditions within the UK hospital in‐patient system. Using meta‐regression, we aimed to test which study and patient factors contribute to any observed variation in prevalence estimates.

## METHODS

This study is reported according to the Preferred Reporting Items for Systematic Reviews and Meta‐Analyses (PRISMA) guidelines 13, and in line with the Meta‐analysis of Observational Studies in Epidemiology (MOOSE) checklist 14. The study did not require ethical approval.

### Search strategy

We searched Medline, Embase, PsychINFO and the Cochrane Central Register of Controlled Trials (CENTRAL) from database inception to 1 May 2018. The complete search strategies can be found in the Supporting information, Fig. S1.

### Study selection

Three authors (E.R., R.M. and S.E.) initially assessed titles and abstracts and reviewed the full text of the remaining articles for inclusion. Any discrepancy was resolved by discussion, and where agreement could not be reached a fourth author (C.D.) was consulted. All relevant references were checked for additional citations.

The review protocol can be found in the Supporting information, Fig. S2. We included studies of any design, using any method of diagnostic ascertainment, that were reported in English, and contained data which enabled the calculation of a prevalence of any of the 26 wholly attributable alcohol conditions as coded in ICD‐10 12. Included studies had to be conducted in one or more of the constituent nations of the United Kingdom (England, Scotland, Wales or Northern Ireland), and report a prevalence in a hospital setting. The setting was defined a priori into four groups: general medical or surgical wards, intensive care units (ICU), accident and emergency (A&E) or mental health in‐patient units.

We excluded studies conducted in specific substance misuse in‐patient settings such as detoxification units or rehabilitation centres. We also excluded studies which reported only a prevalence of ‘alcohol use’ or a compound prevalence such as ‘alcohol or drug dependence’. Studies which reported only historical diagnoses or a ‘history of’ a wholly attributable alcohol condition were also excluded.

### Data extraction

Three authors (E.R., R.M. and S.E.) independently extracted data from all eligible studies using a standardized data extraction spreadsheet and coding dictionary, which can be found in the Supporting information, Figs S3 and S4. In the case of incomplete reporting of data, we searched studies’ online supplementary appendices, and contacted authors as necessary.

The main outcome extracted was prevalence of a wholly attributable alcohol condition. Other parameters which were deemed a priori to have the potential to contribute to variation in prevalence estimates were also extracted. These included: type of patient population, setting, type of diagnostic assessment and whether this was considered robust, mean age of sample, number of females, year of data collection and the constituent nation of the United Kingdom in which the study was conducted. A complete list with descriptions can be found in the Supporting information, Fig. S5.

Some studies reported prevalence estimates in more than one sample of patients, or for more than one wholly attributable alcohol condition; these estimates were extracted separately. Where multiple studies reported on the same patient sample the more conservative estimate was used for meta‐analysis.

### Quality assessment

The quality of each pooled prevalence estimate was assessed using the Grading of Recommendations Assessment, Development and Evaluation (GRADE) framework. Each estimate is given a rating of ‘high’, ‘moderate’, ‘low’ or ‘very low’ quality based upon scores in five domains: risk of bias, inconsistency, indirectness, imprecision and other considerations. Due to the observational nature of the data the default quality score is ‘low’, which can then be up‐ or downgraded according to the quality of the evidence for each estimate. Risk of bias was assessed using a quality assessment tool adapted from the Newcastle–Ottawa Scale 15 (see Supporting information, Fig. S6). Three reviewers (E.R., R.M. and S.E.) independently scored the quality of each study. Any discrepancy was resolved by discussion, and where agreement could not be reached a fourth author (C.D.) was consulted. A complete description of the GRADE quality scoring can be found in the Supporting information, Fig. S7.

### Statistical analysis

Individual prevalence estimates for each of the 26 conditions were stratified into four types of patient population: (1) non‐selective patients, i.e. patients without any pre‐specified characteristics who were sampled solely due to being in an in‐patient setting, (2) patients with specific alcohol diagnoses, (3) patients with specific health disorders and (4) patients within specific medical specialities. Within these groups, prevalence estimates were pooled in meta‐analysis if there were ≥ 2 estimates of the same condition in the same patient population grouping. All estimates from the non‐selective patient population were pooled, and estimates from patients with the same alcohol specific diagnosis, the same health disorder or from the same medical speciality were also pooled. Where only a single prevalence estimate was available within a particular patient population this was reported individually.

True prevalence was likely to vary between studies due to a variety of factors, including differences in study design and diagnostic definitions. As such, a high degree of heterogeneity was anticipated and we chose a priori to perform random‐effects meta‐analysis 16. Prevalence as a measure is bounded by 0 and 100%, and some reported prevalence estimates may be close to these boundaries. As a normal approximation can break down at these extremes we used the Freeman–Tukey double arcsine transformation as a method of stabilizing the variances 17.

Heterogeneity was examined through meta‐regression if there were sufficient numbers of prevalence estimates (*n* ≥ 10) 18. Parameters were sequentially entered as univariate explanatory variables to assess their contribution to the variation in the prevalence estimates. Meta‐regression was restricted a priori to estimates derived from samples in which the mean age of patients was ≥ 18 years of age, as this is the legal limit to purchase alcohol in the United Kingdom, and it was felt to include studies in which patients had a mean age < 18 would not lead to results that were clinically meaningful.

We compared τ^2^ (the between‐study variance) and *I* ^2^ values from the meta‐analysis with the τ^2^ and *I* ^2^ values, and the adjusted *R* ^2^ (the percentage of variation in the prevalence estimates explained by parameter) from each univariate meta‐regression. Where explanatory variables were continuous or binary, results were reported to show how the pooled prevalence estimate changes with each unit increase of the explanatory variable. For categorical variables, results were reported to show how pooled prevalence estimates differ across categories. If there was a reduction in the τ^2^ and *I* ^2^, and an *R* ^2^ > 0 for any individual explanatory variable on univariate meta‐regression, we planned to introduce these variables using a forward approach into a multivariate meta‐regression model, provided that this would not breach the rules of data sparsity and result in overparameterization (in this case defined as fewer than 10 prevalence estimates for each covariate) of the model. All analyses were conducted in Stata IC version 15.

## RESULTS

The search generated 1524 unique results and 41 additional references were identified from citation searching, leading to a total of 1565. We examined 357 full texts and included 124 studies. Two hundred and thirty‐three studies were excluded; common reasons for exclusion included reporting in a non‐in‐patient sample (*n* = 43) and reporting the prevalence of ‘alcohol use’ or alcohol ‘problems’ (*n* = 72). Full reasons for each study's exclusion can be found in the Supporting information, Table S1.

**Figure 2:**
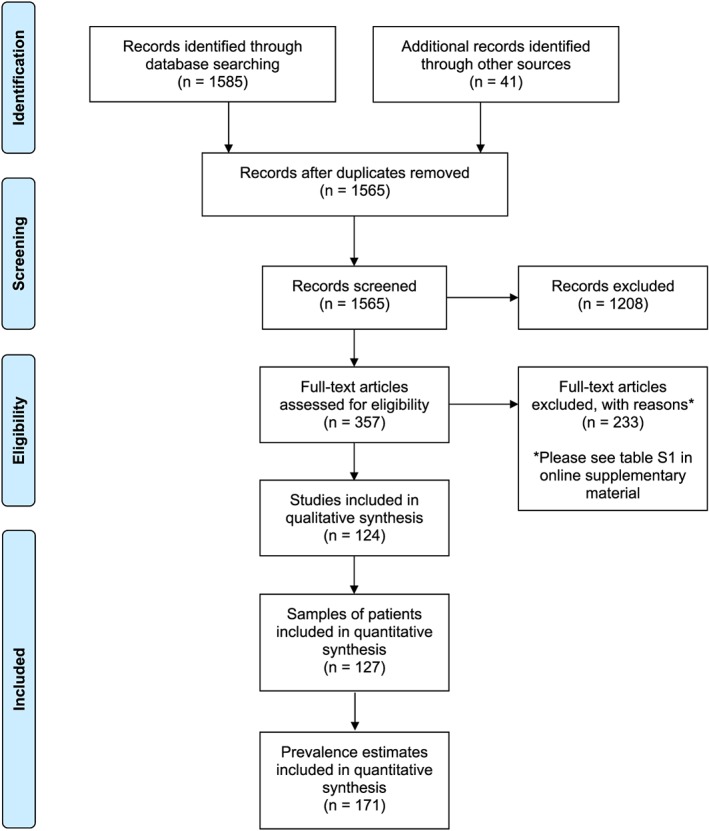
Preferred Reporting Items for Systematic Reviews and Meta‐Analyses (PRISMA) flow diagram describing study selection. [Colour figure can be viewed at wileyonlinelibrary.com]

The 124 included studies consisted of 127 unique patient samples, and reported 171 individual prevalence estimates in a total of 1 657 614 patients. The PRISMA diagram in Fig. 2 describes the study selection. Two studies were RCTs, 14 were cohort studies and 188 were cross‐sectional.

A description of all included study characteristics can be found in the Supporting information, Table S2. No studies reported a prevalence of alcohol‐induced residual and late‐onset psychotic disorder (F10.7), accidental poisoning by and exposure to alcohol (X45), alcoholic fatty liver (K70.0), alcohol‐induced pseudo‐Cushing's syndrome (E24.4), degeneration of the nervous system due to alcohol (G31.2), alcoholic cardiomyopathy (I42.6) or fetal alcohol syndrome (Q86.0); 64 of 124 of the included studies offered no description as to how their reported wholly attributable alcohol condition was diagnosed. Of the 60 that did report a description, 51 were considered to have used robust diagnostic methods to ascertain the diagnosis.

### Non‐selective patients

Eighty‐three prevalence estimates were available in non‐selective patients. Pooled prevalence estimates and their GRADE quality rating stratified by setting can be found in Table 1.

**Table 1:** Pooled prevalence for wholly attributable alcohol conditions in non‐selective patients in the UK hospital system stratified by setting.

Non‐selective patients	Number of prevalence estimates (n)	Number of patients (n)	Prevalence % (95% CI)				
			General medical or surgical ward	ICU	A&E	Mental health in‐patient unit	Overall
Mental and behavioural disorders due to use of alcohol (F10.x)							
Alcohol intoxication F10.0	5	81 990	6.17 (4.41–8.19)	–	12.76 (0.44–37.87)	–	8.99 (0.58–25.38)
Harmful use of alcohol F10.1	29	29 533	16.10 (13.87–18.45)	17.07 (14.23–20.35)	24.23 (11.69–39.56)	36.21 (15.35–60.21)	19.76 (15.61–24.26)
Alcohol dependence F10.2	23	992 784	6.21 (3.57–9.48)	17.41 (14.54–20.71)	16.01 (12.77–19.55)	14.63 (10.36–19.49)	10.25 (7.06–13.96)
Alcohol withdrawal state F10.3	3	48 664	1.17 (0.03–3.47)	–	–	–	1.17 (0.03–3.47)
Alcohol withdrawal state with delirium F10.4	3	7100	1.66 (0.71–3.83)	–	0.06 (0.02–0.16)	0.00 (0.00–2.16)	0.28 (0.00–1.68)
Alcohol‐induced psychotic disorder F10.5	2	43 726	–	–	–	0.04 (0.02–0.08)	0.04 (0.02–0.08)
Poisoning due to alcohol							
Intentional self‐poisoning by and exposure to alcohol X65	1	12 702	–	1.54 (1.34–1.76)	–	–	1.54 (1.34–1.76)*
Liver disorders due to alcohol (K70.x)							
Alcoholic hepatitis K70.1	1	104	0.96 (0.17–5.25)	–	–	–	0.96 (0.17–5.25)*
Alcoholic cirrhosis of liver K70.3	5	18 249	0.66 (0.18–2.39)	2.38 (1.95–2.85)	–	–	2.23 (1.77–2.74)
Alcoholic hepatic failure K70.4	1	257 081	0.22 (0.20–0.24)	–	–	–	0.22 (0.20–0.24)
Alcoholic liver disease, unspecified K70.9	5	644 003	1.24 (1.19–1.29)	1.08 (1.05–1.12)	–	–	2.01 (1.52–2.56)
Gastrointestinal disorders due to alcohol							
Alcohol‐induced acute pancreatitis K85.2	1	257 081	0.41 (0.39–0.44)	–	–	–	0.41 (0.39–0.44)
Alcohol‐induced chronic pancreatitis K86.0	1	257 081	0.41 (0.39–0.44)	–	–	–	0.41 (0.39–0.44)
Alcoholic gastritis K29.2	1	257 081	2.15 (2.09–2.20)	–	–	–	2.15 (2.09–2.20)
Other disorders due to alcohol							
Alcoholic polyneuropathy G62.1	1	301	1.00 (0.34–2.89)	–	–	–	1.00 (0.34–2.89)

All estimates were deemed very low quality according to Grading of Recommendations Assessment Development and Evaluation (GRADE) unless marked by *, which indicates low quality; CI = confidence interval; A&E = accident and emergency; ICU = intensive care unit.

**Figure 3:**
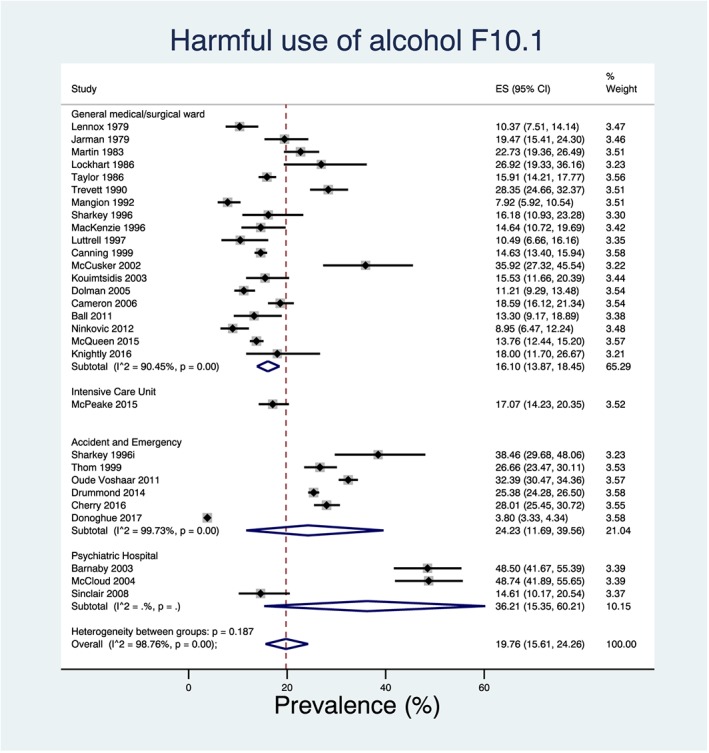
Forest plot of the pooled prevalence for harmful use of alcohol in non‐selective in‐patients in the UK hospital system stratified by setting and ordered by the year in which the study was conducted. [Colour figure can be viewed at wileyonlinelibrary.com]

**Figure 4:**
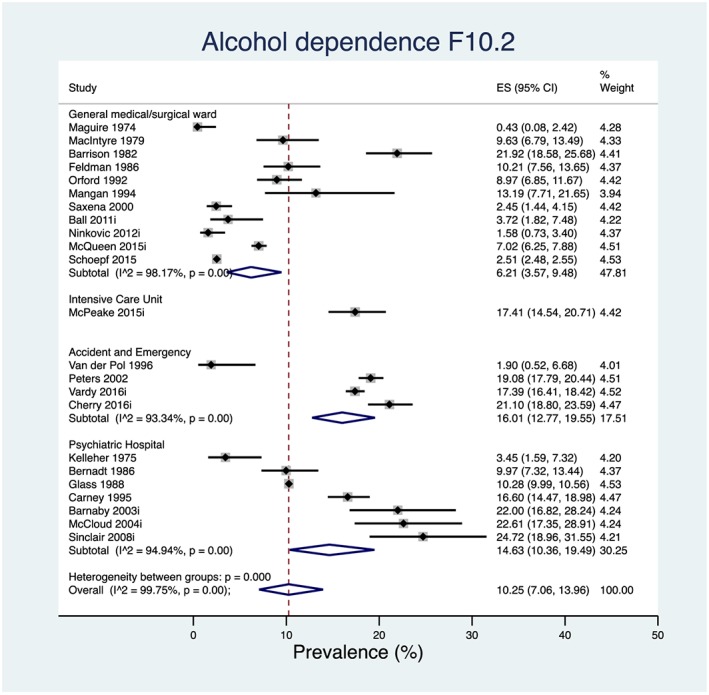
Forest plot of the pooled prevalence for alcohol dependence in non‐selective in‐patients in the UK hospital system stratified by setting and ordered by the year in which the study was conducted. [Colour figure can be viewed at wileyonlinelibrary.com]

Twenty‐nine prevalence estimates were available for a diagnosis of harmful use of alcohol F10.1, 27 of which were considered to have used a robust diagnostic assessment; the overall pooled prevalence using all estimates was 19.76% [95% confidence interval (CI) = 15.61–24.26%]. Twenty‐three prevalence estimates were available for a diagnosis of alcohol dependence F10.2, 18 of which were considered to have used a robust diagnostic assessment; the overall pooled prevalence using all estimates was 10.25% (95% CI = 7.06–13.96%). Both estimates were deemed ‘very low’ quality according to GRADE. Forest plots for these analyses can be found in Figs 3 and 4.

Forest plots for prevalence estimates for all other wholly attributable alcohol conditions and GRADE evidence profiles in non‐selective patients can be found the Supporting information, Fig. S8 and Table S3.

Patients with a specific alcohol diagnosis, specific health disorders and within a specific medical speciality:

Three prevalence estimates were available in patients with a specific alcohol diagnosis, and no prevalence estimates could be combined in meta‐analysis.

Seventy‐five prevalence estimates were available in patients with specific health disorders, and meta‐analysis was possible for eight wholly specific alcohol diagnoses. Four estimates reported a prevalence of harmful use of alcohol in patients with self‐harm (16.14%, 95% CI = 3.07–36.54%), two estimates reported a prevalence of harmful use of alcohol in patients with self‐poisoning (2.50%, 95% CI = 1.01–4.54%), two estimates reported a prevalence of alcohol dependence in patients with serious mental illness (16.76%, 95% CI = 13.10–20.76%), three estimates reported a prevalence of alcohol dependence in patients with self‐harm (11.17% 95% CI = 8.35–14.32%), two estimates reported a prevalence of alcoholic hepatitis in patients with decompensated cirrhosis (9.93%, 95% CI = 7.67–12.44%), five estimates reported a prevalence of alcoholic cirrhosis in patients with decompensated cirrhosis (74.39%, 95% CI = 52.82–91.16%), three estimates reported a prevalence of alcoholic liver disease, unspecified in patients with chronic liver disease (52.70%, 95% CI = 29.55–75.25%) and seven estimates of alcohol‐induced acute pancreatitis in patients with acute pancreatitis (23.55%, 95% CI = 17.39–30.32%). All were deemed to be ‘very low’ quality according to GRADE.

Eleven prevalence estimates were available in patients within a specific medical speciality, and meta‐analysis was possible for two estimates which reported harmful use of alcohol in patients in high‐security hospitals (7.19% 95% CI = 6.03–8.43%). This was deemed to be ‘very low’ quality according to GRADE.

All prevalence estimates, their GRADE evidence profiles and forest plots can be found in the Supporting information, Tables S4–S9 and Figs S9–S10.

### Meta‐regression

Two pooled prevalence estimates contained data from ≥ 10 studies: harmful use of alcohol and of alcohol dependence in non‐selective patients. Tables 2 and 3 show the complete results of the meta‐regression.

**Table 2:** Univariate meta‐regression of study and patient characteristics on prevalence of harmful use of alcohol in non‐selective patients in the UK hospital system.

*Harmful use of alcohol in non‐selective patients*	*Prevalence estimates* (n)	*Patients* (n)	*Prevalence*	*LCI*	*UCI*	*τ* ^2^	I ^2^ (%)		
	28	23 529	20.47	17.39	23.73	0.04	96.99		
	*Prevalence estimates* (n)	*Patients* (n)	*Beta*	*LCI*	*UCI*	*τ* ^2^	I ^2^ (%)	P‐*value* a	*Adjusted* R ^2^ (%)
Is the study a conference abstract? (binary)	28	23 529	−3.60	−16.82	9.62	0.008	82.64	0.58	0
Is the alcohol diagnosis diagnostic assessment robust? (binary)	28	23 529	−10.38	−23.89	3.14	0.006	76.83	0.13	14.20
Mean age of patients (continuous)	13	5838	−0.81	−1.19	−0.44	0.001	42.49	0.001	84.07
Proportion female (continuous)	18	10 925	−0.16	−0.44	0.12	0.011	84.18	0.25	2.51
Total Newcastle–Ottawa scale quality score (continuous)	28	23 529	−0.02	−3.78	3.75	0.008	82.63	0.99	0
Year study conducted (continuous)	19	17 360	0.35	−1.94	2.64	0.006	77.99	0.75	0
Type of setting patients admitted to (categorical)	28	23 529	–	–	–	0.002	52.59	< 0.001	79.26
Nation study conducted in (categorical)	28	23 529	–	–	–	0.008	80.58	0.46	0

a Result from *t*‐test where the null hypothesis was no linear relationship between prevalence and each explanatory variable. LCI = lower confidence interval; UCI = upper confidence interval; τ^2^ = between‐study variance; adjusted *R* ^2^ = percentage of variation in prevalence explained by a particular covariate.

**Table 3:** Univariate meta‐regression of study and patient characteristics on prevalence of alcohol dependence in non‐selective patients in the UK hospital system.

*Alcohol dependence in non‐selective patients*	*Prevalence estimates* (n)	*Patients* (n)	*Prevalence*	*LCI*	*UCI*	*τ* ^2^	I ^2^ (%)		
	23	992 784	10.25	7.06	13.96	0.070	99.75		
	*Prevalence estimates* (n)	*Patients* (n)	*Beta*	*LCI*	*UCI*	*τ* ^2^	I ^2^ (%)	P‐*value* a	*Adjusted* R ^2^ (%)
Is the study a conference abstract? (binary)	23	992 784	−1.39	−11.80	9.03	0.004	96.21	0.79	0
Is the alcohol diagnosis diagnostic assessment robust? (binary)	23	993 784	−1.09	−9.30	7.13	0.004	95.22	0.79	0
Mean age of patients (continuous)	7	7496	−0.80	−1.36	−0.24	0.000	0.00	0.02	100.00
Proportion female (continuous)	10	8588	0.03	−0.29	3.50	0.002	46.80	0.83	0
Total Newcastle–Ottawa scale quality score (continuous)	23	992 784	0.31	−2.14	2.77	0.004	95.00	0.79	0
Year study conducted in (continuous)	16	986 504	0.45	−1.10	2.01	0.004	94.59	0.54	0
Type of setting patients admitted to (categorical)	23	992 784	–	–	–	0.001	64.09	0.009	60.84
Nation study conducted in (categorical)	23	992 784	–	–	–	0.004	95.38	0.40	2.01

a Result from *t*‐test where the null hypothesis was no linear relationship between prevalence and each explanatory variable. LCI = lower confidence interval; UCI = upper confidence interval; τ^2^ = between‐study variance; adjusted *R* ^2^ = percentage of variation in prevalence explained by a particular covariate

When restricted to samples where the mean age of patients was > 18 years, univariate meta‐regression data from 13 prevalence estimates showed that the mean age of patients explained 84.07% of the variation in the prevalence of harmful use, and data from seven prevalence estimates showed that the mean age of patients explained 100% of the variation in prevalence of alcohol dependence. Due to the low number of data points used to estimate these parameters, they should be interpreted with caution. There was strong evidence that for every 1‐year increase in the mean age of patients the prevalence of harmful use of alcohol reduces by 0.81% (95% CI = –1.19 to −0.44%, *P* = 0.001), and strong evidence that for every 1‐year increase in the mean age of patients the prevalence of alcohol dependence reduces by 0.80% (95% CI = –1.36 to −0.24%, *P* < 0.001). Bubble plots displaying the relationship between change in mean age and change in prevalence estimates can be found in the Supporting information, Fig. S11. The alcohol dependence model contained data from only seven prevalence estimates and suffers from overparameterization; accordingly, this result should be interpreted with caution.

Univariate meta‐regression data from 28 prevalence estimates showed that type of setting explained 79.26% of the variation in the prevalence of harmful use, and data from 23 prevalence estimates showed that type of setting explained 60.84% of the variation in the prevalence of alcohol dependence. Individual prevalence estimates for each different type of setting can be found in the forest plots in Figs 3 and 4. However, as setting is a categorical variable with four groups, both models for harmful use of alcohol and alcohol dependence suffer from overparameterization, and these results should be interpreted with caution.

It is notable that neither the year of data collection, whether the study reported a robust diagnostic assessment nor the constituent nation of the United Kingdom in which the study was conducted appeared to substantially explain or contribute to the variation in prevalence estimates on meta‐regression.

Due to data sparsity, we were unable to combine any covariates in multivariate meta‐regression. As there were ≥ 10 studies included for these pooled prevalence estimates funnel plots were generated and can be seen in the Supporting information, Fig. S12.

## DISCUSSION

Approximately one in five in‐patients in hospital in the United Kingdom is using alcohol harmfully, and one in 10 is alcohol‐dependent. Compared to the UK general population this is 10 and eight times higher, respectively 19. High levels of heterogeneity were observed, even within similar patient populations, and the data suggest that the main sources of variation are (a) different types of in‐patient setting and (b) the mean age of patients.

Harmful use ranges from being most prevalent in mental health in‐patient units to least prevalent in general wards. Alcohol dependence ranges from being most prevalent in A&E to least prevalent in general wards. We might have anticipated the prevalence to be higher in mental health in‐patient units and A&E due to the high level of substance use comorbidity in patients with psychiatric illness and those with injuries 20, 21. This appears to be supported by the data which, despite high *I* ^2^ values, demonstrate coherent patterns of prevalence estimates across each setting. It seems reasonable to conclude that setting plays an important role in the variation of the prevalence in hospital settings.

Mean patient age demonstrates strong evidence of a linear association with prevalence of both harmful use and alcohol dependence, after restriction to those samples with a mean age > 18 years. This fits with what we may have expected a priori; however, the strength of association is marked and the mean age of patients may perhaps be more important in determining the overall in‐hospital prevalence than is currently considered in clinical practice.

Little of the variation appears to be explained by year of data collection. Diagnostic criteria and case definitions for wholly attributable alcohol diagnoses have changed substantially during the time‐period covered in the review. Classification has changed with each iteration of the ICD 12, alongside societal and regulatory changes in what is considered a high‐risk level of drinking. Changes over time in coding practice may also have affected the accuracy of prevalence estimates. Throughout the United Kingdom, in‐hospital diagnoses are coded according to the ICD‐10 in a system known as ‘Hospital Episode Statistics’ (HES) 22. During the time–course studied in this review, sequentially more diagnoses have been recorded for each patient per in‐patient episode (e.g. in 2000 the number of coded diagnoses increased from seven to 14, and in 2007 it increased again to 20). This has the potential over time to have underestimated prevalence due to the previous lack of space for alcohol attributable conditions to be recorded. However, when investigated through meta‐regression, the year of data collection did not appear to contribute substantially to the variation in prevalence estimates, nor was there a coherent pattern of the effect of time on visual inspection of the data.

It is also notable that little of the variation appears to be explained by gender, as in the United Kingdom general population estimates would suggest that higher numbers of males meet the criteria for harmful use 19. This may potentially be explained by fewer males, or greater numbers of females seeking in‐patient health care.

The study has several strengths and limitations. We implemented robust methods to conduct the review, using a broad search strategy, and a pre‐defined protocol to capture studies of any design. However, prevalence estimates are not often the primary aim of a study, and as such were often buried within the main body of article texts, prevalence not specifically indexed as an outcome. This may have led to some studies being missed at the inclusion stage. We chose to limit included studies to those only reported in the English language; however, as this review was focused on the United Kingdom it was deemed unlikely that studies would be reported in languages other than English.

A further caveat is that formal evaluation of the quality of all prevalence estimates was either ‘low’ or ‘very low’ according to GRADE, resulting in little variation in estimates being explained by study quality. GRADE was, however, initially designed to focus on intervention studies; as its use has become more widespread there have been critiques regarding its application to observational data. A 2013 survey of public health researchers identified GRADE's limited applicability to diverse epidemiological study types as a key drawback 23. It should be noted that the GRADE default position to begin the overall quality rating at ‘low’ for observational data seemingly does not take into account that observational studies are the most appropriate study design to obtain prevalence estimates. Another drawback is that the criteria requiring fulfilment in order to upgrade the quality rating do not translate easily when the outcome measure is a prevalence estimate. These criteria include a ‘large magnitude of effect’, demonstration of a ‘dose–response gradient’ or consideration of adequate control of confounding 24. Nevertheless, while we could have chosen to simply use the total Newcastle–Ottawa scale score as a quality measure, as has been performed in other systematic reviews of prevalence data 25, GRADE provides a single overall quality rating per estimate, having taken into account additional potential sources of bias including inconsistency and publication bias, which are important in prevalence meta‐analyses 18. As such, GRADE was deemed the most appropriate method to assess quality measures despite the limitations outlined above.

More than half the studies did not report their method of diagnostic ascertainment, and those that did used different methods or screening instruments. Even those studies using the same screening instrument, e.g. the Alcohol Use Disorders Identification Test (AUDIT), often either a different cut‐off was used to diagnose certain disorders (e.g. AUDIT ≥ 15, ≥ 16 or ≥ 20 for alcohol dependence) 26 or ranges were not specified for cut‐offs, leading to the potential for more severe alcohol disease such as dependence being misclassified as harmful use of alcohol 27. This may have the consequence of overestimating the prevalence of those less severe conditions, while underestimating the prevalence of more severe conditions. As such, the finding that whether or not the study reported a robust diagnostic assessment appeared to substantially explain or contribute to the variation in prevalence estimates should be interpreted with caution, given the different diagnostic ascertainment methods used. With regard to the assessment of harmful use and alcohol dependence in non‐selective patients, more than 75% of both diagnoses were considered to be robust. Meta‐regression also demonstrated that whether the study reported a robust diagnostic assessment for the wholly attributable alcohol condition did not appear to contribute substantially to the variation in prevalence estimates.

While alcohol is wholly causative for the conditions discussed in this review, we acknowledge that it is also a contributory factor to a plethora of other conditions. As such, this study does not purport to be an exhaustive exploration of the burden of disease attributable to alcohol in the UK hospital system.

In 1982 McIntosh stated that it was ‘not possible to say, either precisely or even within what limits, what proportion of general hospital patients have alcohol‐related disorders’ 1. We hope this review goes some way to demonstrate this proportion within the in‐patient population. While clinicians may be aware that the prevalence of these conditions is ‘high’ in hospital settings, prevalence is often discussed in the literature without quantification 4. We have attempted to provide this quantification, and are of the opinion that our results represent a much larger magnitude than is currently anecdotally assumed.

Our data support the fact that hospital clinicians should be skilled in the diagnosis and management of alcohol‐related conditions given their ubiquity in this setting. Nevertheless, current rates of formal screening for alcohol‐related conditions in hospital remain low 5. Given the fact that other chronic diseases with a lower in‐hospital prevalence (e.g. diabetes) are both routinely screened for and often have dedicated in‐hospital specialist care teams, our study provides weight to advocate for increased routine universal screening and, given the fact that hospital clinicians often report that they do not feel confident in the management of alcohol use disorders 28, our study adds to the evidence to support improved training concerning alcohol‐related conditions. Due consideration should be given to the need for dedicated in‐patient specialist alcohol care teams to ensure that the widespread problem is being addressed, particularly in the current context of diminishing numbers of specialist alcohol services in the United Kingdom.

## Declaration of interests

None.

## Supporting information


**Figure** **S1** Search Strategies.
**Figure S2** Study Protocol.
**Figure S3** Data Coding Sheet.
**Figure S4** Data Extraction Spreadsheet.
**Figure S5** Extracted Parameters.
**Figure S6** Adapted Newcastle‐Ottawa Checklist.
**Figure S7** GRADE Quality Assessment.
**Figure S8** Forest plots of meta‐analysis for pooled prevalence of wholly attributable alcohol conditions in non‐selective patients in the UK hospital system.
**Figure S9** Forest plots of meta‐analysis for pooled prevalence of wholly attributable alcohol conditions in patients with specific health disorders in the UK hospital system.
**Figure S10** Forest plots of meta‐analysis for pooled prevalence of wholly attributable alcohol conditions in patients within specific medical specialties in the UK hospital system.
**Figure S11** Bubble plots to demonstrate the relationship of mean age to prevalence of wholly attributable alcohol conditions in non‐selective patients in the UK hospital system adjusted for setting.
**Figure S12** Funnel plot of prevalence estimates for wholly attributable alcohol conditions in non‐selective patients in the UK hospital reported by setting.
**Table S1** Excluded Studies.
**Table S2** Description of Included Studies.
**Table S3** GRADE clinical evidence profile for overall prevalence estimates in non‐selective patients.
**Table S4** Pooled prevalence for wholly attributable alcohol conditions in patients with an alcohol diagnosis in the UK hospital system.
**Table S5** GRADE clinical evidence profile for wholly attributable alcohol conditions in patients with an alcohol diagnosis in the UK hospital system.
**Table S6** Pooled prevalence for wholly attributable alcohol conditions in patients with specific health disorders in the UK hospital system.
**Table S7** GRADE clinical evidence profile for wholly attributable alcohol conditions in patients with specific health disorders in the UK hospital system.
**Table S8** Pooled prevalence for wholly attributable alcohol conditions in patients within a specific medical speciality in the UK hospital system.
**Table S9** GRADE clinical evidence profile for wholly attributable alcohol conditions in patients within a specific medical speciality in the UK hospital system.
**Table S10** Preferred Reporting Items for Systematic Reviews and Meta‐Analyses (PRISMA) Checklist.
**Table S11** Meta‐analysis of Observational Studies in Epidemiology (MOOSE) checklist.
