# Supplementary material for: The prevalence of wholly attributable alcohol conditions in the United Kingdom hospital system: a systematic review, meta‐analysis and meta‐regression
Source: Addiction. 2019 Jul 3;114(10):1726–37. doi: 10.1111/add.14642 (PMC6771834; doi:10.1111/add.14642)
Supplement: Supplementary file 1 — Figure S1 Search Strategies. Figure S2 Study Protocol. Figure S3 Data Coding Sheet. Figure S4 Data Extraction Spreadsheet. Figure S5 Extracted Parameters. Figure S6 Adapted Newcastle‐Ottawa Checklist. Figure S7 GRADE Quality Assessment. Figure S8 Forest plots of meta‐analysis for pooled prevalence of wholly attributable alcohol conditions in non‐selective patients in the UK hospital system. Figure S9 Forest plots of meta‐analysis for pooled prevalence of wholly attributable alcohol conditions in patients with specific health disorders in the UK hospital system. Figure S10 Forest plots of meta‐analysis for pooled prevalence of wholly attributable alcohol conditions in patients within specific medical specialties in the UK hospital system. Figure S11 Bubble plots to demonstrate the relationship of mean age to prevalence of wholly attributable alcohol conditions in non‐selective patients in the UK hospital system adjusted for setting. Figure S12 Funnel plot of prevalence estimates for wholly attributable alcohol conditions in non‐selective patients in the UK hospital reported by setting. Table S1 Excluded Studies. Table S2 Description of Included Studies. Table S3 GRADE clinical evidence profile for overall prevalence estimates in non‐selective patients. Table S4 Pooled prevalence for wholly attributable alcohol conditions in patients with an alcohol diagnosis in the UK hospital system. Table S5 GRADE clinical evidence profile for wholly attributable alcohol conditions in patients with an alcohol diagnosis in the UK hospital system. Table S6 Pooled prevalence for wholly attributable alcohol conditions in patients with specific health disorders in the UK hospital system. Table S7 GRADE clinical evidence profile for wholly attributable alcohol conditions in patients with specific health disorders in the UK hospital system. Table S8 Pooled prevalence for wholly attributable alcohol conditions in patients within a specific medical speciality in the UK hospital system. Tabl [file ADD-114-1726-s001.docx]

**Online Supplementary Material**

Figure S1: Search Stretegies

Figure S2: Study Protocol

Figure S3: Data Coding Sheet

Figure S4: Data Extraction Spreadsheet

Figure S5: Extracted Parameters

Figure S6: Adapted Newcastle-Ottawa Checklist

Figure S7: GRADE Quality Assessment

Table S1: Excluded Studies

Table S2: Description of Included Studies

Table S3: GRADE clinical evidence profile for overall prevalence estimates in non-selective patients

Figure S8: Forest plots of meta-analysis for pooled prevalence of wholly attributable alcohol conditions in non-selective patients in the UK hospital system

Table S4: Pooled prevalence for wholly attributable alcohol conditions in patients with an alcohol diagnosis in the UK hospital system

Table S5: GRADE clinical evidence profile for wholly attributable alcohol conditions in patients with an alcohol diagnosis in the UK hospital system

Table S6: Pooled prevalence for wholly attributable alcohol conditions in patients with specific health disorders in the UK hospital system

Table S7: GRADE clinical evidence profile for wholly attributable alcohol conditions in patients with specific health disorders in the UK hospital system

Figure S9: Forest plots of meta-analysis for pooled prevalence of wholly attributable alcohol conditions in patients with specific health disorders in the UK hospital system

Table S8: Pooled prevalence for wholly attributable alcohol conditions in patients within a specific medical speciality in the UK hospital system

Table S9: GRADE clinical evidence profile for wholly attributable alcohol conditions in patients within a specific medical speciality in the UK hospital system

Figure S10: Forest plots of meta-analysis for pooled prevalence of wholly attributable alcohol conditions in patients within specific medical specialties in the UK hospital system

Figure S11: Bubble plots to demonstrate the relationship of mean age to prevalence of wholly attributable alcohol conditions in non-selective patients in the UK hospital system adjusted for setting

Figure S12: Funnel plot of prevalence estimates for wholly attributable alcohol conditions in non-selective patients in the UK hospital reported by setting

Table S10: Preferred Reporting Items for Systematic Reviews and Meta-Analyses (PRISMA) Checklist

Table S11: Meta-analysis of Observational Studies in Epidemiology (MOOSE) checklist

**Figure S1:**

Search strategy for Medline, Embase and PsychINFO using the Ovid interface

Search strategy for the Cochrane Central Register of Controlled Trials (CENTRAL)

It is possible to use an 'explosion' facility within a database using the command 'exp'. This makes use of a hierarchicsal thesaurus and 'explode' a high level term to search for many terms at once. The 'exploded' Alcohol Related Disorders term includes the second order terms: Alcohol-Induced Disorders [C25.775.100.087], Alcoholic Intoxication [C25.775.100.175], Alcoholism [C25.775.100.250], Binge Drinking [C25.775.100.437] and Wernicke Encephalopathy [C25.775.100.625]

**Figure S2: Study Protocol**

**Figure S3: Data Coding Sheet**

**Variable: Study ID (String)**

Free text study name and year e.g. Kirkham 1988

**Variable: Conference Abstract? (Numeric)**

No=0

Yes=1

**Variable: Alcohol Diagnosis ICD 10 DCR (Numeric)**

Alcohol Intoxication F10.0=1

Harmful Use of Alcohol F10.1=2

Alcohol Dependence F10.2=3

Alcohol Withdrawal State F10.3=4

Alcohol Withdrawal State with Delirium F10.4=5

Alcohol induced Psychotic Disorder F10.5=6

Alcohol induced Amnestic Disorder F10.6=7

Alcohol induced Residual and Late Onset Psychotic Disorder F10.7=8

Accidental poisoning by and exposure to alcohol X45=9

Intentional self-poisoning by and exposure to alcohol X65=10

Poisoning by and exposure to alcohol, undetermined intent Y15=11

Alcoholic Fatty Liver K70.0=12

Alcoholic Hepatitis K70.1=13

Alcoholic Fibrosis and Sclerosis of Liver K70.2=14

Alcoholic Cirrhosis of Liver K70.3=15

Alcoholic Hepatic Failure K70.4=16

Alcoholic Liver Disease, Unspecified K70.9=17

Alcohol-induced pseudo-Cushing's syndrome E24.4=18

Degeneration of nervous system due to alcohol G31.2=19

Alcoholic polyneuropathy G62.1=20

Alcoholic myopathy G72.1=21

Alcoholic cardiomyopathy I42.6=22

Alcoholic gastritis K29.2=23

Alcohol-induced acute pancreatitis K85.2=24

Alcohol-induced chronic pancreatitis K86.0=25

Fetal alcohol syndrome (dysmorphic) Q86.0=26

Missing/Not Reported =.

Notes:

Exclude: Methanol poisoning T51.1

**Variable: Alcohol Diagnosis Diagnostic Assessment (String)**

Free text description of method used

NR=.

**Variable: Acceptability of Alcohol Diagnosis Diagnostic Assessment - Adequate to fulfil ICD-10 Criteria (Numeric)**

Unacceptable=0

Acceptable=1

**Variable: Cohort (Numeric)**

Non-selective/Consecutive=1

Alcohol-specific diagnosis cohort=2

Disease-specific cohort=3

Specialty-specific cohort=4

Missing/Not Reported=.

**Variable: Cohort Description (String)**

Brief free text description of patients in cohort

**Variable: Setting (Numeric)**

General hospital ward inpatients=1

General hospital intensive care unit (ICU) inpatients=2

Accident and emergency inpatients=3

Psychiatric hospital inpatients=4

Missing/Not Reported=.

**Variable: Alcohol diagnosis causal or incidental to admission: (Numeric)**

Causal=1

Incidental=2

Unclear=3

**Variable: Year of data collection (Numeric)**

<1960=1

Jan 1st 1960 - 31st Dec 1964=2

Jan 1st 1965 - 31st Dec 1969=3

Jan 1st 1970 - 31st Dec 1974=4

Jan 1st 1975 - 31st Dec 1979=5

Jan 1st 1980 - 31st Dec 1984=6

Jan 1st 1985 - 31st Dec 1989=7

Jan 1st 1990 - 31st Dec 1994=8

Jan 1st 1995 - 31st Dec 1999=9

Jan 1st 2000 - 31st Dec 2004=10

Jan 1st 2005 - 31st Dec 2009=11

Jan 1st 2010 - 31st Dec 2014=12

Jan 1st 2015 - Present =13

Missing/Not reported=.

**Variable: Country (Numeric)**

England=1

Scotland=2

Wales=3

Northern Ireland=4

United Kingdom (constituent Nation prevalence not reported) =5

Missing/Not reported=.

**Variable: Mean Age (Numeric)**

Mean Age=

Missing/Not Reported=.

**Variable: Percentage Female (Numeric)**

Percentage Female=

Missing/Not Reported=.

**Variable: Cases (Numeric)**

Number of cases of alcohol specific diagnosis=

**Variable: Denominator (Numeric)**

Total number of inpatients studied=

**Variable: Notes (String)**

Additional Notes

**Quality Assessment Variables**

**Variable: Representativeness (Numeric)**

Range=0-2

a. Truly representative of the average in the target population (random sample or whole population) (Two points)

b. Somewhat representative of the average in the target population (purposive sampling of specific wards or specialties) (One point)

c. Selected group of users (specific disease) (No points)

d. No description of the derivation of the cohort (No points)

(Two points for A; One point for B; Zero points for C or D)

**Variable: Non-participants (Numeric)**

Range=0-1

a. Comparability between participants and eligible non-participants is established, and the participation rate is satisfactory (>60%) (One point)

b. The participation rate is unsatisfactory, or the comparability between participants and eligible non-participants is unsatisfactory (No points)

c. No description of the response rate or the characteristics of the responders and non-responders (No points)

(One point for A; Zero points for B or C)

**Variable: Sample Size (Numeric)**

Range: 0-1

a) Justified and satisfactory. (>100)

b) Not justified or satisfactory (≤ 99)

(One point for A; Zero points for B)

**Variable: Comparability (Numeric)**

Range=0-2

The subjects are comparable across studies based on age and gender, based on the study design or analysis.

a) Comparable (Two points)

b) Non-Comparable (No points)

(Two points for A; Zero points for B)

**Variable: Ascertainment of the diagnosis of specific ICD-10 wholly attributable alcohol** diagnosis (Numeric)

Range=0-2

a. Established ICD-10 diagnostic code reported (Two points)

b. Appropriate validated measure used to ascertain diagnosis (One point)

c. Non-validated measure used or no description of how alcohol related diagnosis ascertained (No points)

(Two points for A; One point for B; Zero points for C)

**Variable: Prevalence Measurement (Numeric)**

Range=0-1

a. The study reports results that enable calculation of a prevalence numerator and denominator (No points)

b. The study does not report results that enable calculation of a prevalence numerator and denominator (No points)

(One point for A; Zero points for B)

**Variable Total Quality Score (Numeric)**

Range=1-9 (9 indicating highest quality)

**Figure S4: Data Extraction Spreadsheet**


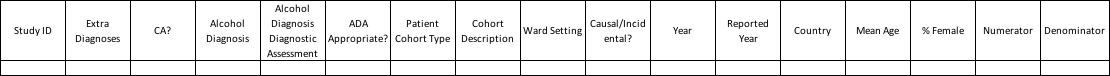


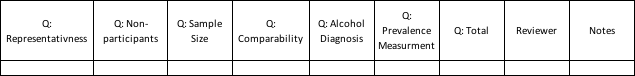


**Figure S5: Extracted Parameters**

- The type of patient population under study. This was defined a priori as either:
  - A non-selective/consecutive population of patients (i.e. patients without any pre-specified characteristics whom were sampled only due to their being in an inpatient setting),
  - A population of patients with a specific alcohol diagnosis (e.g. patients with alcohol dependence in an inpatient setting),
  - A population of patients with a specific disease, which are not explicitly alcohol related (e.g. patients with a lower respiratory tract infection in an inpatient setting), or
  - A population of patients within a specific medical speciality (e.g. patients on a gastroenterology ward)
- Whether the study was a conference abstract
- The diagnostic tool or method used to establish the wholly attributable alcohol condition, and whether that tool or method was considered a valid and appropriate diagnostic measure
- The mean age of the patient cohort
- The percentage of females in the patient cohort
- Whether or not the wholly attributable alcohol diagnosis was causal or incidental to the inpatient admission. The relationship was classified as causal where this was specifically stated or where it could reasonably be inferred from reporting. In some cases there was insufficient evidence to be able to make a clear judgment as to the causal or incidental nature in relation to the inpatient admission
- The year of data collection. This was divided into five-year time bands from 1960 until the present day. For those studies which spanned two or more time-bands the time-band corresponding to the largest proportion of the period over which data was collected was ascribed
- The setting in which the patient was seen. This was defined a priori as either:
  - Any inpatient medical or surgical ward
  - Any intensive care or critical care unit (ICU)
  - Any accident and emergency (A&E) department
  - Any psychiatric hospital
- The constituent nation of the United Kingdom the study was conducted in

**Figure S6: Adapted Newcastle-Ottawa Checklist**

**Figure S7: GRADE Quality Assessment**

Risk of Bias: Risk of bias was assessed using a quality assessment tool adapted from the Newcastle-Ottawa Scale. The maximum available score was nine, higher scores indicating higher quality. There was deemed to be minimal risk of bias, and thus the overall quality rating was not downgraded, if the pooled average total quality score was ≥ 7. The overall quality rating was downgraded by one level (e.g. a reduction from *low* to *very low*) if the weighted average total quality score per estimate was 4-6, and the overall quality rating was downgraded by two levels if the weighted average total quality score per estimate was ≤ 3.

Inconsistency: Inconsistency refers to the level of heterogeneity between prevalence estimates, and was assessed using the I^2^ statistic. The overall quality rating was not downgraded if I^2^ was < 50%, was downgraded by one level if I^2^ was ≥ 50% and < 75%, and by two levels if I^2^ was ≥ 75%.

Indirectness: Indirectness refers to the extent to which the study population or outcome differ from those specified in the inclusion criteria. As all studies fulfilled the inclusion criteria no estimate was downgraded due to indirectness.

Imprecision: Imprecision refers to the extent to which we are uncertain about the estimates, and based on examination of the confidence interval (CI). As no guidelines exist as to what represents an appropriate precision of a prevalence, the following criteria were developed: the overall quality rating was not downgraded if the precision (i.e. half the CI) was < 5%, was downgraded by one level if the precision was ≥ 5 % and < 10%, and by two levels if the precision was ≥ 10%.

Other considerations: GRADE also considers other potential sources of bias, and publication bias was assessed within this domain. Consistent with the Cochrane handbook,(1) in cases where ≥ 10 studies were included in the meta-analysis funnel plots were produced. These were visually inspected to explore the possibility of preferential publication of small studies reporting inflated prevalence estimates, and in addition we performed Egger’s test for small study effects.(2) Where evidence was suggestive of publication bias the overall quality rating was downgraded by one level.

**Table S1: Excluded Studies**

| Abou-Saleh, M. T., et al. (2008). "The prevalence of substance misuse and emotional and behavioral problems in adolescents admitted to pediatric wards and those attending the antenatal clinic." Addictive Disorders & Their Treatment **7**(1): 25-30. | Exclude: Reports ‘alcohol use’ |
| --- | --- |
| Adams, R. H. (1986). "An accident and emergency department's view of self-poisoning: a retrospective study from the United Norwich Hospitals 1978-1982." Human Toxicology **5**(1): 5-10. | Exclude: Does not report calculable prevalence of alcohol self-poisoning |
| Agarwal, M. and K. Gaskell (1996). "Clinical features of alcoholic suicide attempters/non-attempters." Psychiatric Bulletin **20**(11): 656-659. | Exclude: Specific Alcohol Detoxification Unit Patients |
| Andoh, B. (1999). "Selected characteristics of absconders and non-absconders from mental hospitals: a comparison." The International journal of social psychiatry **45**(2): 117-124. | Exclude: People with a 'history of alcohol abuse' not current diagnosis |
| Anwar, M. U., et al. (2005). "Smoking, substance abuse, psychiatric history, and burns: trends in adult patients." Journal of Burn Care & Rehabilitation **26**(6): 493-501. | Exclude: Does not report calculable prevalence of alcoholism |
| Aspinall, R. J., et al. (2011). "Rapid identification, triage and management of alcohol related hospital admissions." Gut **2)**: A12-A13. | Exclude: Reports number of patients referred to an alcohol service. No denominator reported. |
| Azorin, J.-M., et al. (2013). "Mixed states with predominant manic or depressive symptoms: Baseline characteristics and 24-month outcomes of the EMBLEM cohort." Journal of Affective Disorders **146**(3): 369-377. | Exclude: Reports non calculable prevalence in multiple counties and mixed outpatient and inpatient sample |
| Ballinger, B. R. (1974). "Self-medication in psychiatric patients." International Journal of Social Psychiatry **20**(3-4): 180-185. | Exclude: Reports compound outcomes of personality disorder or alcoholism |
| Barrison, I. G., et al. (1982). "Detecting excessive drinking among admissions to a general hospital." Health Trends **14**(3): 80-83. | Exclude: Duplicate Abstract of Barrison 1982 |
| Barrow, A., et al. (2012). "Late presentations of minor head injury." Emergency Medicine Journal **29**(12): 983-988. | Exclude: Reports alcohol intoxication at time of injury not whilst in A+ E |
| Barrowclough, C., et al. (2010). "Integrated motivational interviewing and cognitive behavioural therapy for people with psychosis and comorbid substance misuse: randomised controlled trial." BMJ (Clinical research ed.) **341 (no pagination)**(c6325). | Exclude: Unclear if inpatient cohort |
| Barry, E., et al. (2015). "Ethnic group variations in alcohol-related hospital admissions in England: does place matter?" Ethnicity & Health **20**(6): 557-563. | Exclude: Reports alcohol-related number admissions no calculable prevalence of alcohol specific condition in cohort |
| Beattie, J. O., et al. (1986). "Children intoxicated by alcohol in Nottingham and Glasgow, 1973-84." British medical journal (Clinical research ed.) **292**(6519): 519-521. | Exclude: No Denominator reported unable to calculate prevalence of alcohol poisoning |
| Bennett, S. P. H., et al. (2009). "Inhalation injury associated with smoking, alcohol and drug abuse: an increasing problem." Burns : journal of the International Society for Burn Injuries **35**(6): 882-887. | Exclude: Reports "smoking, alcohol or drug use" prevalence |
| Bennewith, O., et al. (2005). "Factors associated with the non-assessment of self-harm patients attending an Accident and Emergency Department: results of a national study." Journal of Affective Disorders **89**(1-3): 91-97. | Exclude: Reports "Illegal drugs/alcohol' prevalence |
| Bergen, H., et al. (2010). "Epidemiology and trends in non-fatal self-harm in three centres in England: 2000-2007." British Journal of Psychiatry **197**(6): 493-498. | Exclude: Reports 'alcohol involvement' |
| Bergen, H., et al. (2012). "Premature death after self-harm: A multicentre cohort study." The Lancet **380**(9853): 1568-1574. | Exclude Reports Prevalence of 'Alcohol problems' |
| Bewley, T. H. and O. Ben-Arie (1968). "Morbidity and mortality from heroin dependence. 2. Study of 100 consecutive inpatients." British Medical Journal **1**(5594): 727-730. | Exclude: Reports prevalence of excessive heavy drinkers |
| Bhala, N., et al. (2016). "Ethnic Variations in Liver- and Alcohol-Related Disease Hospitalisations and Mortality: The Scottish Health and Ethnicity Linkage Study." Alcohol and alcoholism (Oxford, Oxfordshire) **51**(5): 593-601. | Exclude: Reports rates of first hospitalisations due to alcohol attributable disease |
| Blad, W., et al. (2016). "Alcohol-related admissions to a central London hospital: Too little coding; Too much resource." Gut **65 (Supplement 1)**: A212-A213. | Exclude: Reports number of harmful drinkers admitted to A+ E; No denominator reported |
| Blenkiron, P., et al. (2000). "The timing of acts of deliberate self-harm: is there any relation with suicidal intent, mental disorder or psychiatric management?" Journal of Psychosomatic Research **49**(1): 3-6. | Exclude: Reports alcohol use at time of assessment |
| Bourke, J. B. (1975). "Variation in annual incidence of primary acute pancreatitis in Nottingham, 1969-74." Lancet (London, England) **2**(7942): 967-969. | Exclude: Duplicate data of Giggs 1998 |
| Bowden, B., et al. (2018). "Risk of suicide following an alcohol-related emergency hospital admission: An electronic cohort study of 2.8 million people." PLoS ONE [Electronic Resource] **13**(4): e0194772. | Exclude: Reports frequency and incidence rates of death from suicide by alcohol admission codes. |
| Bristow, M. and A. Clare (1992). "Prevalence and characteristics of at-risk drinkers among elderly acute medical in-patients." British Journal of Addiction **87**(2): 291-294. | Exclude: Reports prevalence of 'at risk drinkers' |
| Bruce, M., et al. (2012). "Ethnic differences in reported unmet needs among male inpatients with severe mental illness." Journal of Psychiatric and Mental Health Nursing **19**(9): 830-838. | Exclude: Duplicate data from Bruce 2014 |
| Burke, A. W. (1976). "Attempted suicide among Asian immigrants in Birmingham." British Journal of Psychiatry **128**: 528-533. | Exclude: No prevalence of alcohol related specific diagnosis reported |
| Burke, A. W. (1978). "Attempted suicide among Commonwealth immigrants in Birmingham." The International journal of social psychiatry **24**(1): 7-11. | Exclude: Reports Drug or Alcohol Addiction |
| Busby, J., et al. (2017). "How do population, general practice and hospital factors influence ambulatory care sensitive admissions: a cross sectional study." BMC Family Practice **18**(1): 67. | Exclude: Reports Alcohol Related Diseases prevalence |
| Camidge, D. R., et al. (2003). "The epidemiology of self-poisoning in the UK." British Journal of Clinical Pharmacology **56**(6): 613-619. | Exclude: Reports alcohol or substance dependency no calculable prevalence of an inpatient cohort |
| Cappai, A., et al. (2017). "Substance misuse in personality disorder and schizophrenia: Findings and clinical implications from a high secure hospital." Journal of Forensic Practice **19**(3): 217-226. | Exclude: Reports historical harmful alcohol use and dependency not current diagnoses |
| Carrà, G. and S. Johnson (2009). "Variations in rates of comorbid substance use in psychosis between mental health settings and geographical areas in the UK. A systematic review." Social Psychiatry and Psychiatric Epidemiology **44**(6): 429-447. | Exclude: Systematic review. All references checked for includable studies |
| Chakrabarti, S., et al. (2013). "Dipeptidyl peptidase-4 inhibitors and glucagon-like peptide 1 agonists are not linked with severe hypoglycaemia: An emergency department perspective." Diabetic Medicine **1)**: 148-149. | Exclude: Alcohol excess reported |
| Charlesworth, A., et al. (2015). "Acute pancreatitis associated with severe hypertriglyceridaemia; A retrospective cohort study." International journal of surgery (London, England) **23**: 23-27. | Exclude: Reports that alcohol excess could have been causative for some patients, but doesn’t report acute pancreatitis by aetiology |
| Charzynska, K., et al. (2011). "Comorbidity patterns in dual diagnosis across seven European sites." The European Journal of Psychiatry **25**(4): 179-191. | Exclude: No separate UK results reported |
| Cheng, C., et al. (2017). "Alcohol-Related Dementia: A Systemic Review of Epidemiological Studies." Psychosomatics **58**(4): 331-342. | Exclude: Non inpatient UK sample prevalences reported |
| Cherpitel, C. J. (1994). "Alcohol and injuries resulting from violence: a review of emergency room studies." Addiction (Abingdon, England) **89**(2): 157-165. | Exclude: Reports positive Blood Alcohol Concentration 'BAC' in violent injuries prevalence not specific alcohol disease specific prevalence |
| Chick, J. (1991). "Early intervention for hazardous drinking in the general hospital." Alcohol & Alcoholism. Supplement **1**: 477-479. | Exclude: Review article, all references checked for additional includes |
| Chick, J. (1991). "Early intervention for hazardous drinking in the general hospital." Alcohol & Alcoholism. Supplement **1**: 477-479. | Exclude: No library or journal able to source full text, unclear from abstract if prevalence of alcohol specific diagnosis reported |
| Cobain, K., et al. (2011). "Brief interventions in dependent drinkers: a comparative prospective analysis in two hospitals." Alcohol & Alcoholism **46**(4): 434-440. | Exclude: Problem drinkers recruited into study. No calculable prevalence reported |
| Cochrane, R. (1977). "Mental illness in immigrants to England and Wales: an analysis of mental hospital admissions, 1971." Social Psychiatry **12**(1): 25-35. | Exclude: No UK specific figures reported; "Alcohol-problems" reported |
| Cochrane, R. (1980). "Mental illness in England, in Scotland and in Scots living in England." Social Psychiatry **15**(1): 9-15. | Exclude: Reports Alcoholism accounts for 30.6% of all male Scottish migrant's admissions to hospital in England, no denominator reported |
| Cochrane, R. and S. S. Bal (1989). "Mental hospital admission rates of immigrants to England: a comparison of 1971 and 1981." Social Psychiatry and Psychiatric Epidemiology **24**(1): 2-11. | Exclude: Reports rates of mental hospital admissions in England per 100,000 population, alcohol category is compound of alcohol abuse, dependence, psychosis and non-dependent abuse |
| Compton, S. A. and O. Daly (1986). "Rates of admission to six Northern Ireland psychiatric hospitals of patients with primary alcohol-related diagnoses." The Ulster medical journal **55**(2): 154-159. | Exclude: Reports change in % of alcohol related admissions |
| Cooper, J., et al. (2005). "Suicide after deliberate self-harm: A 4-year cohort study." American Journal of Psychiatry **162**(2): 297-303. | Exclude: Reports only subjects who died with alcohol misuse |
| Coppen, A., et al. (1983). "Dexamethasone suppression test in depression and other psychiatric illness." The British Journal of Psychiatry **142**: 498-504. | Exclude: Reported on cohort is rehabilitation unit inpatients with alcohol misuse |
| Corrigan, G. V., et al. (1986). "Alcohol dependence among general medical inpatients." British Journal of Addiction **81**(2): 237-245. | Exclude: Study conducted in the Republic of Ireland |
| Craig, D. G., et al. (2012). "Staggered overdose pattern and delay to hospital presentation are associated with adverse outcomes following paracetamol-induced hepatotoxicity." British Journal of Clinical Pharmacology **73**(2): 285-294. | Exclude: Duplicate Cohort |
| Currie, C., et al. (2016). "The impact of alcohol care teams on emergency secondary care use following a diagnosis of alcoholic liver disease - a national cohort study." BMC Public Health **16**: 685. | Exclude: No alcohol specific diagnosis prevalence calculable. No denominator reported, uses a HES extract of alcoholic liver disease from both inpatient and outpatient set. |
| Da Cruz, D., et al. (2011). "Emergency department contact prior to suicide in mental health patients." Emergency Medicine Journal **28**(6): 467-471. | Exclude: Does not report an in hospital prevalence. Individuals are a hypothetical cohort of patients attending ED who subsequently died by suicide |
| Daly, A. and D. Walsh (2010). "Psychiatric disorder treated in acute general hospitals: a comparison with psychiatric units and hospitals." Irish Journal of Medical Science **179**(1): 85-89. | Exclude: Uses data from the Republic or Ireland |
| Dargan, D. P., et al. (2016). "Three-year outcomes of intracapsular femoral neck fractures fixed with sliding hip screws in adults aged under sixty-five years." Injury **47**(11): 2495-2500. | Exclude: Reports those with a documented history of alcohol dependence, not a current diagnosis |
| Dasgupta, P. and J. Barber (2004). "Admission patterns of patients with personality disorder." Psychiatric Bulletin **28**(9): 321-323. | Exclude: Reports prevalence of 'alcohol or substance misuse' |
| De Burca, C., et al. (2013). "Substance use amongst mentally disordered offenders in medium security: Prevalence and relationship to offending behaviour." Journal of Forensic Practice **15**(4): 259-268. | Exclude: Reports 'heavy past use' |
| Dean, G., et al. (1981). "First admissions to psychiatric hospitals in south-east England in 1976 among immigrants from Ireland." British Medical Journal **282**(6279): 1831-1833. | Exclude: Reports first hospital admission rate for alcohol disorders among Irish immigrants/100000 of the population not in hospital prevalence |
| Dean, G., et al. (1981). "First admissions of native-born and immigrants to psychiatric hospitals in South-East England 1976." The British journal of psychiatry : the journal of mental science **139**: 506-512. | Exclude: Reports first hospital admission rate for alcohol disorders/100000 of the population not in hospital prevalence |
| Deluca, P., et al. (2010). "Challenges and solutions in implementing screening and brief interventions for hazardous alcohol use in accident and emergency departments." Alcoholism: Clinical and Experimental Research **34 (6)**: 294A. | Exclude: Duplicate data of Drummond 2014 |
| Dennis, M., et al. (1997). "An examination of the accident and emergency management of deliberate self harm." Journal of Accident and Emergency Medicine **14**(5): 311-315. | Exclude: Reports prevalence of alcohol dependence among episodes of self-harm not among individual patients. |
| Dent, A., et al. (2010). "The impact of frequent attenders on a UK emergency department." European Journal of Emergency Medicine **17**(6): 332-336. | Exclude: Reports compound prevalence of 'concurrent alcohol use or long-term alcohol dependence' and 'presenting complaint of alcohol intoxication, not all cases' |
| Duffy, J. and N. Kreitman (1993). "Risk factors for suicide and undetermined death among in-patient alcoholics in Scotland." Addiction **88**(6): 757-766. | Exclude: No reported denominator unable to calculate prevalence |
| Eagles, J. M. and J. A. Besson (1985). "Changes in the incidence of alcohol-related problems in north-east Scotland, 1974-1982." The British journal of psychiatry : the journal of mental science **147**: 39-43. | Exclude: No calculable prevalence of alcohol specific diagnosis reported |
| Eardley, W. G., et al. (2006). "Human bite injury in North East England--the impact of alcohol intake on a mode of violent assault." Journal of the Royal Army Medical Corps **152**(1): 22-25. | Exclude: Reports people who were 'under the influence of alcohol' |
| El-Maaytah, M., et al. (2008). "The effect of the new "24 hour alcohol licensing law" on the incidence of facial trauma in London." The British journal of oral & maxillofacial surgery **46**(6): 460-463. | Exclude: Reports 'alcohol associated head and neck trauma' |
| Exiara, T., et al. (2012). "Hospital-acquired pneumonia in non-ICU patients in a rural general hospital." Clinical Microbiology and Infection **18**: 276-277. | Exclude: Unclear country of study |
| Finnegan, A., et al. (2007). "A review of one year of British Armed Forces mental health hospital admissions." Journal of the Royal Army Medical Corps **153**(1): 26-31. | Exclude: Military Hospital Inpatients |
| Fortune, Z., et al. (2011). "Clinical and economic outcomes from the UK pilot psychiatric services for personality-disordered offenders." International Review of Psychiatry **23**(1): 61-69. | Exclude: Reports a 'history of alcohol misuse' |
| Foster, J. H., et al. (2000). "Measurement of quality of life in alcohol-dependent subjects by a cancer symptoms checklist." Alcohol **20**(2): 105-110. | Exclude: Alcohol dependent cohort recruited; Nil calculable prevalence of alcohol specific diagnosis |
| Fothergill, N. J. and K. Hashemi (1990). "A prospective study of assault victims attending a suburban A&E department." Archives of emergency medicine **7**(3): 172-177. | Exclude: Reports alcohol consumption in relation to assault |
| Gaughran, F., et al. (2014). "Cardiovascular risk, lifestyle choices and substance use in the first year of psychosis." Early Intervention in Psychiatry **1)**: 27. | Exclude: Unclear if inpatient cohort |
| Geary, T., et al. (2012). "A national service evaluation of the impact of alcohol on admissions to Scottish intensive care units." Anaesthesia **67**(10): 1132-1137. | Exclude: Reports 'alcohol-related admissions' |
| Gho, J. M. I. H., et al. (2018). "An electronic health records cohort study on heart failure following myocardial infarction in England: Incidence and predictors." BMJ Open **8 (3) (no pagination)**(e018331). | Exclude: Reports "Excess Alcohol Consumption" |
| Goldacre, M. J. and S. E. Roberts (2004). "Hospital admission for acute pancreatitis in an English population, 1963-98: Database study of incidence and mortality." British Medical Journal **328**(7454): 1466-1469. | Exclude: No aetiology of pancreatitis reported |
| Goldbeck, R., et al. (2012). "Alcohol and drug misuse, risk of re-admission to a general hospital and psychiatric contact." Scottish Medical Journal **57**(1): 60. | Exclude: Reports on a hypothetical cohort of patients identified as high risk based on previous A&E visits, does not report an in hospital calculable prevalence |
| Goodall, J. A. and C. Bryan (1988). "The low incidence of alcoholic cirrhosis in the islands of Lewis and Harris." Scottish Medical Journal **33**(2): 229-230. | Exclude: No denominator reported for cases in hospital. Unclear if number of incident cases refers to those in hospital or in wider population |
| Graham, J. D. P., et al. (1979). "Self poisoning - a decennial survey from Cardiff." Public Health **93**(4): 223-229. | Exclude: Reports 'alcohol or other drugs' |
| Green, B. H. and E. C. Griffiths (2014). "Hospital admission and community treatment of mental disorders in England from 1998 to 2012." General Hospital Psychiatry **36**(4): 442-448. | Exclude: Reports 'Alcohol related admissions' |
| Green, M. A., et al. (2017). "Trends in alcohol-related admissions to hospital by age, sex and socioeconomic deprivation in England, 2002/03 to 2013/14." BMC Public Health **17**(1): 412. | Exclude: Reports number of admissions to hospital with alcohol conditions but no denominator to calculate in hospital prevalence |
| Greig, E., et al. (2012). "Maternal and neonatal outcomes following methadone substitution during pregnancy." Archives of Gynecology & Obstetrics **286**(4): 843-851. | Exclude: Reports prevalence of alcohol abuse in pregnant women attending substance misuse clinic not inpatients |
| Griffin, E., et al. (2014). "Characteristics of hospital-treated intentional drug overdose in Ireland and Northern Ireland." BMJ Open **4 (7) (no pagination)**(e005557). | Exclude: Reports 'Alcohol involvement' in intentional poisoning cases |
| Haddock, G., et al. (2018). "Influence of age on outcome of psychological treatments in first-episode psychosis." British Journal of Psychiatry **188**(3): 250-254. | Exclude: Includes inpatients and day patients in cohort, no separately calculable inpatient prevalence |
| Halbgewachs, C., et al. (2011). "Substance abuse amongst adolescents in modern suburban ireland." Acta Paediatrica, International Journal of Paediatrics **463)**: 20. | Exclude: Conducted in the Republic of Ireland |
| Halikas, J. A., et al. (1981). "Psychiatric diagnosis among female alcoholics." Curr Alcohol **8**: 283-291. | Exclude: Study conducted in the USA |
| Harris, J., et al. (2003). "Prior Alcoholics Anonymous (AA) affiliation and the acceptability of the Twelve Steps to patients entering UK statutory addiction treatment." Journal of Studies on Alcohol **64**(2): 257-261. | Exclude: Entering Alcohol Detoxification Unit |
| Harrison, D. and J. Chick (1994). "Trends in alcoholism among male doctors in Scotland." Addiction **89**(12): 1613-1617. | Exclude: Reports admissions and discharges of male doctors with alcohol problems |
| Hatton, J., et al. (2009). "Drinking patterns, dependency and life-time drinking history in alcohol-related liver disease." Addiction **104**(4): 587-592. | Exclude: Inpatient and outpatient cohort, no denominator reported for total inpatient population |
| Hawton, K. (1981). "The long-term outcome of psychiatric morbidity detected in general medical patients." Journal of Psychosomatic Research **25**(3): 237-243. | Exclude: Follow up study of inpatients discharged from hospital |
| Hawton, K., et al. (2007). "Self-harm in England: A tale of three cities: Multicentre study of self-harm." Social Psychiatry and Psychiatric Epidemiology **42**(7): 513-521. | Exclude: Reports alcohol consumption in relation to self-harm |
| Hawton, K., et al. (2012). "Epidemiology and nature of self-harm in children and adolescents: Findings from the multicentre study of self-harm in England." European Child and Adolescent Psychiatry **21**(7): 369-377. | Exclude: Reports alcohol involvement with self-harm |
| Haynes, C. L. and G. A. Cook (2009). "An audit of health education services within UK hospitals." Journal of Evaluation in Clinical Practice **15**(4): 704-712. | Exclude: Reports an audit for number of patients screened for alcohol misuse |
| Hearnshaw, S. A., et al. (2011). "Acute upper gastrointestinal bleeding in the UK: patient characteristics, diagnoses and outcomes in the 2007 UK audit." Gut **60**(10): 1327-1335. | Exclude: Reports a 'history of alcohol excess' |
| Heatley, M. K. and J. Crane (1989). "Acute pancreatitis as a cause of sudden or unexpected death in Northern Ireland." Ulster Medical Journal **58**(1): 51-55. | Exclude: Reports alcoholic pancreatitis prevalence in only deaths of people with pancreatitis |
| Henson, V. L. and D. S. Vickery (2005). "Patient self discharge from the emergency department: who is at risk?" Emergency Medicine Journal **22**(7): 499-501. | Exclude: Reports 'under the influence of drugs or alcohol' |
| Herbert, A., et al. (2015). "Violence, self-harm and drug or alcohol misuse in adolescents admitted to hospitals in England for injury: a retrospective cohort study." BMJ Open **5**(2): e006079. | Exclude: Reports 'drug or alcohol misuse' |
| Herbert, A., et al. (2017). "Time-trends in rates of hospital admission of adolescents for violent, self-inflicted or drug/alcohol-related injury in England and Scotland, 2005-11: population-based analysis." Journal of public health (Oxford, England) **39**(1): 65-73. | Exclude: Reports drug/alcohol related injury |
| Heydtmann, M. and S. A. McDonald (2013). "Survival and re-admission of patients admitted with alcoholic liver disease to a West of Scotland hospital." Scottish Medical Journal **58**(3): 134-138. | Exclude: Reports on an initial cohort of alcoholic liver disease inpatients |
| Heyes, G. J., et al. (2017). "Predictors for 1-year mortality following hip fracture: a retrospective review of 465 consecutive patients." European journal of trauma and emergency surgery : official publication of the European Trauma Society **43**(1): 113-119. | Exclude: Reports 'alcohol excess' |
| Hiles, S., et al. (2015). "General hospital-treated self-poisoning in England and Australia: comparison of presentation rates, clinical characteristics and aftercare based on sentinel unit data." Journal of Psychosomatic Research **78**(4): 356-362. | Exclude: Reports alcohol users among any substance misusers |
| Hocking, M. A. (1989). "Assaults in south east London." Journal of the Royal Society of Medicine **82**(5): 281-284. | Exclude: Reports 'Alcohol a contributing factor' |
| Hodgins, S., et al. (2008). "From conduct disorder to severe mental illness: Associations with aggressive behaviour, crime, and victimization." Psychological Medicine **38**(7): 975-987. | Exclude: Duplicate data of Hodgins 2007 |
| Hodgins, S., et al. (2009). "Do community mental health teams caring for severely mentally ill patients adjust treatments and services based on patients' antisocial or criminal behaviours?.[Erratum appears in Eur Psychiatry. 2009 Dec;24(8):552 Note: Sainz-Fuentes, R [corrected to Sainz-Fuertes, R]]." European Psychiatry: the Journal of the Association of European Psychiatrists **24**(6): 373-379. | Exclude: Duplicate data from Hodgins 2007 |
| Holding, T. A., et al. (1977). "Parasuicide in Edinburgh--a seven-year review 1968-74." The British journal of psychiatry : the journal of mental science **130**: 534-543. | Exclude: Reports 'Problem with alcohol' |
| Holloway, A. S., et al. (2007). "The effect of brief interventions on alcohol consumption among heavy drinkers in a general hospital setting." Addiction **102**(11): 1762-1770. | Exclude: Reports prevalence of 'excess drinking' not alcohol specific diagnosis |
| Holloway, A. and H. E. Watson (2000). "Screening for hazardous/harmful alcohol consumption amongst general hospital in-patients: establishing concurrent validity of the Alcohol Use Disorders Identification Test in the UK." Journal of Substance Use **5**(3): 263-271. | Exclude: Reports number of people drinking over nationally defined limits not diagnostic category. Conducts AUDIT on this subset but no numbers are reported figure too small to adequately classify numbers |
| Holmes, W. J. M., et al. (2009). "Alcohol and burns - Impact on a regional burns centre." Burns **1)**: S10-S11. | Exclude: Abstract of Holmes 2010 |
| Hoskins, R. and J. Benger (2013). "What is the burden of alcohol-related injuries in an inner city emergency department?" Emergency medicine journal : EMJ **30**(3): e21. | Exclude: Reports prevalence of alcohol related attendances to A+E not specific alcohol diagnoses. No numbers quoted for alcoholic pancreatitis |
| Howarth, M., et al. (2012). "Association of water softness and heavy alcohol consumption with higher hospital admission rates for alcoholic liver disease." Alcohol & Alcoholism **47**(6): 688-696. | Exclude: No inpatient alcohol specific prevalence calculable |
| Hoy, A. R. (2017). "Which young people in England are most at risk of an alcohol-related revolving-door readmission career?" BMC Public Health **17**(1): 185. | Exclude: Reports on cohort of young people with alcohol related ICD-10 code, but no calculable alcohol specific diagnosis prevalence available in cohort studied |
| Huang-Doran, I., et al. (2015). "Early in-patient management of alcohol-related liver disease: Results of a liver care bundle to improve quality of care." Gut **1)**: A186. | Exclude: Alcoholic Cirrhosis Cohort; No prevalence calculable |
| Hudson, S. A., et al. (2008). "Validation of a screening instrument for post-traumatic stress disorder in a clinical sample of older adults." Aging & Mental Health **12**(5): 670-673. | Exclude: Inpatients or Day patients no breakdown given |
| Iozzino, L., et al. (2015). "Prevalence and risk factors of violence by psychiatric acute inpatients: A systematic review and meta-analysis." PLoS ONE **10 (6) (no pagination)**(e012853). | Exclude: Reports 'lifetime history of alcohol abuse' |
| Jacobs, R., et al. (2015). "Determinants of hospital length of stay for people with serious mental illness in England and implications for payment systems: a regression analysis." BMC Health Services Research **15**: 439. | Exclude: Reports 'alcohol and substance misuse' |
| Jamieson, C. P., et al. (1999). "The thiamin, riboflavin and pyridoxine status of patients on emergency admission to hospital." Clinical nutrition (Edinburgh, Scotland) **18**(2): 87-91. | Exclude: Reports 'alcohol intake' |
| Jauhar, P. and A. S. Watson (1995). "Severity of alcohol dependence in the East End of Glasgow." Alcohol & Alcoholism **30**(1): 67-70. | Exclude: Reports on a cohort of alcohol dependent inpatients, no prevalence calculable |
| Johnstone, M. A., et al. (1991). "Children's admission to I.C.U. with ethanol poisoning." Pediatric Reviews and Communications **6**(2): 119-122. | Exclude: Case series of ethanol poisoning. No denominator reported to calculate prevalence |
| Jones, J. J. and R. V. Jeffreys (1983). "Head injury patients admitted to general hospitals in Merseyside." Injury **14**(6): 483-488. | Exclude: Reports number of patients who had taken alcohol |
| Keaney, F., et al. (2011). "Physical health problems among patients seeking treatment for substance use disorders: A comparison of drug dependent and alcohol dependent patients." Journal of Substance Use **16**(1): 27-37. | Exclude: Reports on mixed in and outpatient cohort |
| Kellezi, B., et al. (2017). "The impact of psychological factors on recovery from injury: A multicentre cohort study." Social Psychiatry and Psychiatric Epidemiology **52**(7): 855-866. | Exclude: Reports alcohol problems |
| Kendrick, D., et al. (2017). "Psychological morbidity and health-related quality of life after injury: Multicentre cohort study." Quality of Life Research: An International Journal of Quality of Life Aspects of Treatment, Care & Rehabilitation **26**(5): 1233-1250. | Exclude: No alcohol specific diagnosis prevalence reported in study cohort |
| Keown, P., et al. (2008). "Retrospective analysis of hospital episode statistics, involuntary admissions under the Mental Health Act 1983, and number of psychiatric beds in England 1996-2006." BMJ **337**: a1837. | Exclude: Reports 'drug and alcohol related problems' |
| Kilich, S. and M. A. Plant (1981). "Regional variations in the levels of alcohol-related problems in Britain." British Journal of Addiction **76**(1): 47-62. | Exclude: Reports alcohol-related first admissions |
| Kreitman, N. and M. Schreiber (1979). "Parasuicide in young Edinburgh women, 1968--75." Psychological Medicine **9**(3): 469-479. | Exclude: No calculable prevalence of chronic alcohol dependence reported |
| Kriese, S., et al. (2015). "Complex symptom burden and unmet need in end-stage liver disease." Gut **1)**: A264-A265. | Exclude: Mixed inpatient and outpatient cohort |
| Krishel, S. and L. J. Baraff (1996). "Feasibility of routine screening of patients for alcohol abuse." Academic emergency medicine : official journal of the Society for Academic Emergency Medicine **3**(9): 903-905. | Exclude: Study conducted in the USA |
| Latcham, R. W. and N. Kreitman (1984). "Regional variation in alcoholism rates in Britain: The effect of provision and use of services." International Journal of Epidemiology **13**(4): 442-446. | Exclude: Reports number of admission per population not inpatient prevalence |
| Latcham, R. W., et al. (1984). "Regional variations in British alcohol morbidity rates: a myth uncovered? I: Clinical surveys." British medical journal (Clinical research ed.) **289**(6455): 1341-1343. | Exclude: Sample of inpatients with alcohol misuse interviewed |
| Lawrie, S. M., et al. (1995). "Psychosis and substance abuse: cause, effect or coincidence?" Scottish Medical Journal **40**(6): 174-176. | Exclude: Reports 'Excessive consumption of alcohol ' |
| Lewis, S., et al. (2018). "Randomised controlled trial of cognitive-behavioural therapy in early schizophrenia: Acute-phase outcomes." British Journal of Psychiatry **181**(S43): s91-s97. | Exclude: Includes inpatients and day patients in cohort, no separately calculable inpatient prevalence |
| Lewsey, J., et al. (2016). "Hospital admissions and associated costs of alcoholic liver disease in Scotland between 1991 and 2011." Gut **65 (Supplement 1)**: A270. | Exclude: Reports incident ALD admissions, no denominator reported or prevalence calculable |
| Lloyd, G., et al. (1982). "Screening for problem drinkers among medical inpatients." Drug Alcohol Depend. **10**(4): 355-359. | Exclude: Reports prevalence of 'alcohol problems or if the patient had previously been in alcohol treatment' |
| Lumsden, J., et al. (2005). "The prevalence of early onset alcohol abuse in mentally disordered offenders." Journal of Forensic Psychiatry & Psychology **16**(4): 651-659. | Exclude: Reports a 'history of alcohol abuse' not current diagnosis |
| MacIntyre, D., et al. (2004). "Substance misuse in a high security hospital: Three years of urine drug testing at the State Hospital, Carstairs." Journal of Forensic Psychiatry & Psychology **15**(4): 606-619. | Exclude: Nil specific alcohol diagnosis prevalence reported |
| Mackle, I. J., et al. (2006). "One year outcome of intensive care patients with decompensated alcoholic liver disease." British Journal of Anaesthesia **97**(4): 496-498. | Exclude: Reports on alcoholic liver disease cohort, no calculable alcohol specific diagnosis prevalence |
| Maguire, H. C. (1988). "Alcohol and acute hospital admissions." Irish Medical Journal **81**(1): 32-35. | Exclude: Based in Dublin, Ireland |
| Mallawaarachchi, N., et al. (2016). "Does the use of non-selective beta-blockers in cirrhosis patients with ascites result in increased mortality?" Journal of Hepatology **1)**: S278-S279. | Exclude: No prevalence of alcoholic cirrhosis reported |
| Mangrolia, N., et al. (2010). "United kingdom model for end-stage liver disease and model for end stage liver disease scores predict outcome after oesophageal variceal haemorrhage." Gut **59 (Supplement 1)**: A104. | Exclude: Reports the number of cases of oesophageal variceal haemorrhage which were alcohol related |
| Marley, W. D., et al. (2015). "Alcohol-Related Fracture Admissions: A Retrospective Observational Study." The Ulster medical journal **84**(2): 94-97. | Exclude: Patients admitted to trauma and orthopaedic unit. Unclear if inpatient cohort |
| Martin, D. and D. Smith "Unipolar mania: A discrete nosological entity within a broad bipolar spectrum? Information from the Bipolar Disorder Research Network Study." Bipolar Disorders. Conference: 10th International Conference on Bipolar Disorder. Miami Beach, FL United States. Conference Publication: **15**(SUPPL.1): 155. | Exclude: Not inpatient cohort |
| Mason, C., et al. (2015). "Early in-patient management of alcohol-related liver disease: Results of a liver care bundle to improve quality of care." United European Gastroenterology Journal **1)**: A154. | Exclude: Alcoholic Liver Disease Cohort, No prevalence calculable |
| Mather, H. M. and D. H. Marjot (1989). "Alcohol-related admissions to a psychiatric hospital: A comparison of Asians and Europeans." British Journal of Addiction **84**(3): 327-329. | Exclude: Reports on cohort of inpatients with 'alcohol problems' |
| Mathers, D. C., et al. (1991). "Cannabis use in a large sample of acute psychiatric admissions." British Journal of Addiction **86**(6): 779-784. | Exclude: Reports 'people drinking X units/day' |
| Mayou, R. and K. Hawton (1986). "Psychiatric disorder in the general hospital." The British Journal of Psychiatry **149**: 172-190. | Exclude: Systematic Review all references check for inclusion |
| Mayou, R., et al. (1991). "Psychiatric problems among medical admissions." International Journal of Psychiatry in Medicine **21**(1): 71-84. | Exclude: Reports 'problem drinking' |
| Mayou, R., et al. (1994). "Use of psychiatric services by patients referred to a consultation unit." General Hospital Psychiatry **16**(5): 354-357. | Exclude: Reports Alcohol Problems |
| McDonald, S. A., et al. (2010). "Hospitalization of hepatitis C-diagnosed individuals in Scotland for decompensated cirrhosis: a population-based record-linkage study." European Journal of Gastroenterology & Hepatology **22**(1): 49-57. | Exclude: Reports alcohol related hospital admissions |
| McGrath, J. C. (2008). "Fear of falling after brain injury." Clinical Rehabilitation **22**(7): 635-645 | Exclude: Reports prevalence of 'premorbid alcohol misuse' or a history of alcohol misuse not current diagnosis |
| McMahon, C., et al. (2003). "Changes in patterns of excessive alcohol consumption in 25 years of high security hospital admissions from England and Wales." Criminal Behaviour & Mental Health **13**(1): 17-30. | Exclude: Reports 'problem drinkers' |
| McMurray, J. J., et al. (1987). "Trends in analgesic self-poisoning in West-Fife, 1971-1985." The Quarterly journal of medicine **65**(246): 835-843. | Exclude: Reports 'alcohol taken in conjunction with self-poisoning' |
| McPeake, J., et al. (2014). "Alcohol related admissions to ICU: An 18 month prospective cohort study." Critical Care Medicine **1)**: A1459. | Exclude: Conference Abstract of McPeake 2015 |
| Miller, P. M., et al. (2000). "Differences between patients with schizophrenia within and without a high security psychiatric hospital." Acta Psychiatrica Scandinavica **102**(1): 12-18 | Exclude: Reports history of alcohol misuse prior to index admission not current diagnosis |
| Millett, E. R. C., et al. (2015). "Risk factors for hospital admission in the 28 days following a community-acquired pneumonia diagnosis in older adults, and their contribution to increasing hospitalisation rates over time: A cohort study." BMJ Open **5 (12) (no pagination)**(e008737). | Exclude: Reports prevalence of excessive alcohol consumption |
| Misselbrook, G. P. and N. Sudhan (2014). "Epidemiology and critical care management of patients admitted after intentional self-poisoning." Critical Care **1)**: S25-S26. | Exclude: Reports 'a history of alcohol misuse', not current diagnosis |
| Mitchell, V., et al. (2018). "Exploring psychiatric morbidity in the scottish burn injury population." Critical Care Medicine **46 (Supplement 1)**: 762. | Exclude: Reports compound prevalence "mental and behavioural disorders due to use of alcohol" |
| Morleo, M., et al. (2011). "Under-reporting of foetal alcohol spectrum disorders: an analysis of hospital episode statistics." BMC Pediatrics **11**: 14. | Exclude: Reports incidence rate of FASD per population, no in hospital denominator calculable |
| Mostafa, S. M. and B. V. S. Murthy (2002). "Alcohol-associated admissions to an adult intensive care unit: an audit." European Journal of Anaesthesiology (EJA) **19**(3): 193-196. | Exclude: No alcohol specific diagnosis prevalence reported |
| Mulinga, J. D. (1999). "Elderly people with alcohol-related problems: Where do they go?" International Journal of Geriatric Psychiatry **14**(7): 564-566. | Exclude: Reports on cohort of alcohol dependent inpatients; No calculable prevalence of alcohol specific diagnosis reported |
| Murphy, H. B. and G. Vega (1982). "Schizophrenia and religious affiliation in Northern Ireland." Psychological Medicine **12**(3): 595-605. | Exclude: Reports first admission rates |
| Newton, A., et al. (2007). "Impact of the new UK licensing law on emergency hospital attendances: a cohort study." Emergency Medicine Journal **24**(8): 532-534. | Exclude: Reports 'alcohol related' attendance |
| Northcote, R. J., et al. (1983). "Changing pattern of alcohol abuse in female acute medical admissions." British medical journal (Clinical research ed.) **286**(6379): 1702. | Exclude: Reports number of people whose admissions were considered to be due to the effects of alcohol |
| Northridge, D. B., et al. (1986). "Association between liberalisation of Scotland's liquor licensing laws and admissions for self poisoning in West Fife." British Medical Journal **293**(6560): 1466-1468. | Exclude: Reports 'alcohol taken with cases of self-poisoning' |
| O'Loughlin, S. and J. Sherwood (2005). "A 20-year review of trends in deliberate self-harm in a British town, 1981-2000." Social Psychiatry & Psychiatric Epidemiology **40**(6): 446-453. | Exclude: Reports alcohol consumed at time of injury |
| Owen, G. S., et al. (2009). "Mental capacity, diagnosis and insight in psychiatric in-patients: a cross-sectional study." Psychological Medicine **39**(8): 1389-1398. | Exclude: Reports compound outcome drug or alcohol misuse |
| Owens, L., et al. (2016). "A Randomized Controlled Trial of Extended Brief Intervention for Alcohol-Dependent Patients in an Acute Hospital Setting." Alcohol and alcoholism (Oxford, Oxfordshire) **51**(5): 584-592. | Exclude: Baseline group alcohol use disorder, no calculable prevalence of an alcohol specific diagnosis |
| Pakpoor, J., et al. (2014). "Alcohol-use disorders and multiple sclerosis risk: A national record-linkage study." Multiple Sclerosis **1)**: 148-149. | Exclude: No prevalence calculable; No denominator given |
| Papastergiou, V., et al. (2014). "Nine scoring models for short-term mortality in alcoholic hepatitis: Cross-validation in a biopsy-proven cohort." Alimentary Pharmacology and Therapeutics **39**(7): 721-732. | Exclude: Primary cohort alcoholic hepatitis, no calculable alcohol specific diagnosis within cohort |
| Parfitt, V. J. and R. Bhake (2012). "An analysis of all cases of severe hypoglycaemia presenting to a major teaching hospital over one year." Diabetic Medicine **1)**: 131. | Exclude: Reports prevalence of alcohol-induced hypoglycaemia |
| Parker, R., et al. (2017). "Clinical and microbiological features of infection in alcoholic hepatitis: an international cohort study." Journal of gastroenterology **52**(11): 1192-1200. | Exclude: Included cohort of alcoholic hepatitis patient, no calculable prevalence of alcohol specific diagnosis |
| Parkinson, K., et al. (2016). "Prevalence of alcohol related attendance at an inner city emergency department and its impact: a dual prospective and retrospective cohort study." Emergency Medicine Journal **33**(3): 187-193. | Exclude: Reports prevalence of 'alcohol-related attendance' and BAC positivity |
| Paton, L., et al. (2016). "Utility of the PRE-DELIRIC delirium prediction model in a Scottish ICU cohort." The Journal of the Intensive Care Society **17**(3): 202-206. | Exclude: 'Reports alcohol or drug misuse' |
| Pentland, B., et al. (2005). "Late mortality after head injury." Journal of Neurology, Neurosurgery & Psychiatry **76**(3): 395-400. | Exclude: Reports alcohol dependence prevalence in a cohort of patients who died |
| Pentland, B., et al. (1986). "Head injury in the elderly." Age and ageing **15**(4): 193-202. | Exclude: Reports 'alcohol as a contributory factor' |
| Peppiatt, R., et al. (1978). "Blood alcohol concentrations of patients attending an accident and emergency department." Resuscitation **6**(1): 37-43. | Exclude: Reports 'detectable blood alcohol concentration' |
| Petrova, M., et al. (2011). "Patient characteristics and outcomes in a 'hub and spoke model' for liver transplantation provision: The South West Liver Unit/King's College experience." Gut **2)**: A37. | Exclude: Unclear if inpatient cohort |
| Phang, I., et al. (2012). "Paediatric head injury admissions over a 10-year period in a regional neurosurgical unit." Scottish Medical Journal **57**(3): 152-156. | Exclude: Reports 'Alcohol involvement' |
| Phillips, P. and S. Johnson (2003). "Drug and alcohol misuse among in-patients with psychotic illnesses in three inner-London psychiatric units." Psychiatric Bulletin **27**(6): 217-220. | Exclude: Reports 'drug and/or alcohol misuse or dependence' |
| Pirmohamed, M., et al. (2000). "The burden of alcohol misuse on an inner-city general hospital." QJM - Monthly Journal of the Association of Physicians **93**(5): 291-295. | Exclude: Reports 'alcohol induced medical illness' |
| Potts, J. R., et al. (2013). "Determinants of long-term outcome in severe alcoholic hepatitis." Alimentary Pharmacology & Therapeutics **38**(6): 584-595. | Exclude: Reports on cohort of alcoholic hepatitis patients, no calculable alcohol diagnosis prevalence reported |
| Quayle, M., et al. (1998). "Alcohol and secure hospital patients: I. An examination of the nature and prevalence of alcohol problems in secure hospital patients." Psychology, Crime & Law **4**(1): 27-41. | Exclude: Reports numbers of 'low' 'medium' and 'high dependency based on SADD and Hilton scales but this adds up to 100% unclear how correlates with ICD10 diagnosis of alcohol dependence |
| Rainey, S. R., et al. (2015). "The impact of violence reduction initiatives on emergency department attendance." Scottish Medical Journal **60**(2): 90-94. | Exclude: Reports 'alcohol consumption' |
| Ramayya, A. and P. Jauhar (1997). "Increasing incidence of Korsakoff's psychosis in the east end of Glasgow." Alcohol & Alcoholism **32**(3): 281-285. | Exclude: Reports Incidence of Korsakoff’s Psychosis in the general population |
| Rasoul, D., et al. (2016). "Psychiatric co-morbidities and tendencies in patients with non-ischaemic heart failure (NIHF) - A large observational cohort study spanning 14 years." Heart **102 (Supplement 6)**: A7. | Exclude: Reports compound outcome 'mainly made up by alcohol and tobacco abuse' |
| Ratib, S., et al. (2014). "Diagnosis of liver cirrhosis in England, a cohort study, 1998-2009: A comparison with cancer." American Journal of Gastroenterology **109**(2): 190-198. | Exclude: Not inpatient cohort |
| Redfern, T. R., et al. (1988). "The impact of alcohol ingestion on the orthopaedic and accident service." Alcohol and alcoholism (Oxford, Oxfordshire) **23**(5): 415-419. | Exclude: Reports alcohol use at the time of trauma |
| Reeves, G. K., et al. (2014). "Hospital admissions in relation to body mass index in UK women: a prospective cohort study." BMC Medicine **12**: 45. | Exclude: Reports Alcohol intake in Million Women Study Participants |
| Rhouma, O., et al. (2013). "Facial injuries in Scotland 2001-2009: epidemiological and sociodemographic determinants." British Journal of Oral & Maxillofacial Surgery **51**(3): 211-216. | Exclude: 'Reports alcohol related facial injury |
| Ritchie, G., et al. (2004). "The detection and treatment of substance abuse in offenders with major mental illness: an intervention study." Medicine, Science & the Law **44**(4): 317-326. | Exclude: Reports historical problems associated with alcohol |
| Rizzo, M. and S. Smith (2012). "Risk assessment and risk management among severely mentally ill patients requiring hospitalisation." Journal of Forensic Psychiatry & Psychology **23**(4): 497-509. | Exclude: Duplicate data from Hodgins 2007 |
| Roberts, S. E., et al. (2005). "Trends in mortality after hospital admission for liver cirrhosis in an English population from 1968 to 1999." Gut **54**(11): 1615-1621. | Exclude: Reports number of admissions of alcohol related diagnosis but no denominator, prevalence not calculable |
| Roberts, S. E., et al. (2014). "Mortality following acute pancreatitis: social deprivation, hospital size and time of admission: record linkage study." BMC Gastroenterology **14**: 153. | Exclude: Reports prevalence of number of cases of acute pancreatitis not number of patients as reported in paper |
| Roberts, S. E., et al. (2013). "The incidence of acute pancreatitis: Impact of social deprivation, alcohol consumption, seasonal and demographic factors." Alimentary Pharmacology and Therapeutics **38**(5): 539-548. | Exclude: Denominator of cases derived from inpatient and primary care records, not pure inpatient sample |
| Robertson, M., et al. (2017). "AIMS65, a risk stratification score for upper gastrointestinal bleeding, accurately predicts mortality in patients presenting with acute variceal bleeding: A multicenter cohort study." Gastrointestinal Endoscopy **85 (5 Supplement 1)**: AB69. | Exclude: Unclear in which country research undertaken |
| Roche, A. M., et al. (2006). "From data to evidence, to action: Findings from a systematic review of hospital screening studies for high risk alcohol consumption." Drug and Alcohol Dependence 83(1): 1-14. | Exclude: Systematic review checked for all additional references |
| Rowland, N., et al. (1992). "Screening for patients at risk of alcohol related problems: the results of the York District Hospital Alcohol Study." Health Trends **24**(3): 99-102. | Exclude: Reports number of people 'at risk' of drinking problems |
| Sadler, S., et al. (2017). "Understanding the alcohol harm paradox: an analysis of sex- and condition-specific hospital admissions by socio-economic group for alcohol-associated conditions in England." Addiction (Abingdon, England) **112**(5): 808-817. | Exclude: Reports admission rates per population, no denominator given no in hospital prevalence calculable. |
| Sagar, N., et al. (2013). "An audit to evaluate the use of the alcohol fast screening tool in acute medical admissions in a district general hospital." Gut **2)**: A12-A13. | Exclude: Reports prevalence of 'drinking over the national limit' |
| Scheffler, A., et al. (1987). "Alcohol-related problems amongst selected hospital patients and the cost incurred in their care." British Journal of Addiction **82**(3): 275-283. | Exclude: Reports on alcohol related discharges but no denominator reported to calculate prevalence |
| Schoepf, D. and R. Heun (2014). "Bipolar disorder and comorbidity: Increased prevalence and increased relevance of comorbidity for hospital-based mortality during a 12.5-year observation period in general hospital admissions." Journal of Affective Disorders **169**: 170-178. | Exclude Duplicate reporting of data from Schoepf 2015 |
| Scott, M. L., et al. (2011). "The impact of alcohol on critical care referrals and admissions in portsmouth, UK." Intensive Care Medicine **1)**: S23. | Exclude: Doesn’t report prevalence of wholly attributable alcohol condition |
| Shaik-Dawood, A. M., et al. (2011). "Long-term survival of alcoholic liver disease following critical care admission: A national cohort study." Journal of the Intensive Care Society **12 (1)**: 69. | Exclude: Reports on cohort of patients in ICU with alcoholic liver disease, no denominator reported for calculable prevalence. No other alcohol specific diagnosis prevalence calculable |
| Shepherd, J., et al. (1988). "Assault: characteristics of victims attending an inner-city hospital." Injury **19**(3): 185-190. | Exclude: Reports 'alcohol intake' |
| Shepherd, R. M., et al. (1995). "Prevalence of alcohol histories in medical and nursing notes of patients admitted with self poisoning.[Erratum appears in BMJ 1995 Oct 14;311(7011):990]." BMJ **311**(7009): 847. | Exclude: Reports prevalence of 'excessive drinking' |
| Simpson, A., et al. (2011). "The relationship between substance use and exit security on psychiatric wards." Journal of Advanced Nursing **67**(3): 519-530. | Exclude: Reports alcohol use |
| Simpson, T., et al. (2001). "Saliva alcohol concentrations in accident and emergency attendances." Emergency medicine journal : EMJ **18**(4): 250-254. | Exclude: Reports positive alcohol saliva test |
| Singh, R., et al. (2018). "Prevalence of depression after TBI in a prospective cohort: The SHEFBIT study." Brain Injury **32**(1): 84-90. | Exclude: Reports 'alcohol intoxication at time of injury' |
| Singhal, A., et al. (2014). "Risk of self-harm and suicide in people with specific psychiatric and physical disorders: comparisons between disorders using English national record linkage." Journal of the Royal Society of Medicine **107**(5): 194-204. | Exclude: Reports number of admissions due to alcohol abuse in England. No denominator quoted in hospital prevalence not calculable |
| Siriwardena, A. K., et al. (2012). "Antioxidant therapy does not reduce pain in patients with chronic pancreatitis: the ANTICIPATE study." Gastroenterology **143**(3): 655-663.e651. | Exclude: Unclear if inpatient cohort |
| Stanley, J. L., et al. (2016). "Use of novel psychoactive substances by inpatients on general adult psychiatric wards." BMJ Open **6**(5): e009430. | Exclude: Reports compound outcome of harmful use or dependence |
| Stewart, D. and L. Bowers (2015). "Substance use and violence among psychiatric inpatients." Journal of Psychiatric and Mental Health Nursing **22**(2): 116-124. | Exclude: Reports a 'history of excessive alcohol use' |
| Stewart, D., et al. (2015). "Nursing interventions for substance use during psychiatric hospital admissions: Clinical context and predictors." International Journal of Mental Health Nursing **24**(6): 527-537. | Exclude: Reports 'problematic alcohol use as defined by doctor' |
| Strang, I., et al. (1978). "Head injuries in accident and emergency departments at Scottish hospitals." Injury **10**(2): 154-159. | Exclude: Reports 'alcohol use' |
| Teo, A. I. and J. G. Cooper (2013). "The epidemiology and management of adult poisonings admitted to the short-stay ward of a large Scottish emergency department." Scottish Medical Journal **58**(3): 149-153. | Exclude: Reports 'alcohol/substance misuse' |
| Thomson, S. J., et al. (2010). "A study of patients with cirrhosis admitted to nontransplant general intensive care in the UK: Prevalence, case mix, outcomes and evaluation of critical illness and disease-specific scoring systems." Critical Care **1)**: S181. | Exclude: Duplicate abstract of Thomson 2010 |
| Thomson, S. J., et al. (2008). "Chronic liver disease--an increasing problem: a study of hospital admission and mortality rates in England, 1979-2005, with particular reference to alcoholic liver disease." Alcohol & Alcoholism **43**(4): 416-422. | Exclude: Reports rates of admission for alcoholic liver disease, in hospital prevalence not calculable |
| Tolley, K. and N. Rowland (1991). "Identification of alcohol‐related problems in a general hospital setting: a cost‐effectiveness evaluation." British Journal of Addiction **86**(4): 429-438. | Exclude: Reports number of patients screened for at risk drinking by different professional groups. Unclear if multiple patients being screened by different professionals or if unique patients, as such unclear if double counting and true prevalence reported. |
| Tulloch, A. D., et al. (2012). "Timing, prevalence, determinants and outcomes of homelessness among patients admitted to acute psychiatric wards." Social Psychiatry and Psychiatric Epidemiology **47**(7): 1181-1191. | Exclude: Reports 'lifetime diagnosis of drug or alcohol disorder' |
| Van Rompaey, B., et al. (2009). "Risk factors for delirium in intensive care patients: a prospective cohort study." Critical Care (London, England) **13**(3): R77. | Exclude: Reports alcohol use |
| Vardy, J., et al. (2009). "Are emergency department staffs' perceptions about the inappropriate use of ambulances, alcohol intoxication, verbal abuse and violence accurate?" Emergency medicine journal : EMJ **26**(3): 164-168. | Exclude: Reports prevalence of patients whom were 'acutely intoxicated', unclear which substance, and prevalence of patients with 'complaints related to chronic alcohol abuse |
| Virgo, N., et al. (2001). "The prevalence and characteristics of co-occurring serious mental illness (SMI) and substance abuse or dependence in the patients of Adult Mental Health and Addictions Services in eastern Dorset." Journal of Mental Health **10**(2): 175-188 | Exclude: Reports 'current problematic use (abuse or dependence)' compound outcome in subset of acute inpatients |
| Wadd, S. and C. Papadopoulos (2014). "Drinking behaviour and alcohol-related harm amongst older adults: analysis of existing UK datasets." BMC Research Notes **7**: 741. | Exclude: Reports total alcohol-related hospital admissions (i.e. wholly and partly attributable) per 1,000 population not calculable in hospital prevalence |
| Waddell, T. S. and W. S. Hislop (2003). "Analysis of alcohol-related admissions in gastroenterology, cardiology and respiratory medicine." Scottish Medical Journal **48**(4): 114-116. | Exclude: Reports 'alcohol-related conditions' |
| Waring, W. S., et al. (2008). "Acute ethanol coingestion confers a lower risk of hepatotoxicity after deliberate acetaminophen overdose." Academic Emergency Medicine **15**(1): 54-58. | Exclude: Reports 'alcohol excess' |
| Watson, H. (2000). "Problem drinkers among acute care inpatients." Nursing Standard **14**(40): 32-35. | Exclude: Reports number of 'potential problem drinkers' |
| Watson, H. E., et al. (1991). "Alcohol problems among women in a general hospital ward." British Journal of Addiction **86**(7): 889-894. | Exclude: Reports 'newly identified' problem drinkers, doesn’t fully report all alcohol abuse in cohort unable to accurately ascertain prevalence of alcohol abuse given newly diagnosed numbers reported |
| Webber, K. and A. N. Davies (2012). "An observational study to determine the prevalence of alcohol use disorders in advanced cancer patients." Palliative Medicine **26**(4): 360-367. | Exclude: Inpatient and Outpatient sample |
| Wheatley, M. (1998). "The prevalence and relevance of substance use in detained schizophrenic patients." The Journal of Forensic Psychiatry **9**(1): 114-129. | Exclude: Reports previous alcohol use not alcohol specific diagnosis |
| Wilkinson, S., et al. (2002). "Admissions to hospital for deliberate self-harm in England 1995-2000: An analysis of Hospital Episode Statistics." Journal of Public Health Medicine **24**(3): 179-183. | Exclude: Reports 'alcohol involvement' |
| Williams, E. R., et al. (2001). "Psychiatric status, somatisation, and health care utilization of frequent attenders at the emergency department: a comparison with routine attenders." Journal of Psychosomatic Research **50**(3): 161-167. | Exclude: Reports 'alcohol related diagnoses' |
| Williams, S., et al. (2005). "Hospital admissions for drug and alcohol use in people aged under 45." British Medical Journal **330**(7483): 115. | Exclude: Reports admission rates not calculable in hospital prevalence |
| Willox, D. G. (1985). "Self poisoning. A review of patients seen in the Victoria Infirmary, Glasgow." Scottish Medical Journal **30**(4): 220-224. | Exclude: Reports alcohol use at time of overdose |
| Wood, C. L., et al. (2013). "An audit of epidemiology of patients with non-traumatic coma presenting to a tertiary paediatric intensive care unit in South East Scotland." Developmental Medicine and Child Neurology **1)**: 60-61. | Exclude: No prevalence calculable for alcohol intoxication |
| Wright, J. and A. Kariya (1997). "Aetiology of assault with respect to alcohol, unemployment and social deprivation: A Scottish accident and emergency department case-control study." Injury **28**(5-6): 369-372. | Exclude: Reports recent consumption of alcohol |
| Wright, J. and A. Kariya (1997). "Assault patients attending a Scottish accident and emergency department." Journal of the Royal Society of Medicine **90**(6): 322-326. | Exclude: Reports alcohol consumption at time of assault |
| Wynne, H., et al. (1987). "Age and self-poisoning: the epidemiology in Newcastle upon Tyne in the 1980s." Human Toxicology **6**(6): 511-515. | Exclude: Reports overdose 'in conjunction with alcohol' |
| Yates, K., et al. (2000). "Changes in psychiatric admissions in rural northern England." Journal of Mental Health **9**(1): 105-111. | Exclude: Reports 'alcohol or drug related problems' |

**Table S2: Charicteristics of Included Studies**

| **Study ID** | **Alcohol Diagnosis** | **Alcohol Diagnosis Diagnostic Assessment** | **Patient Sample Description** | **Setting** | **Year of data collection** | **Country** | **Mean Age** | **% Female** | **Total NOS Quality Score** | **Sample Size (n)** |
| --- | --- | --- | --- | --- | --- | --- | --- | --- | --- | --- |
| **Mental and behavioural disorders due to use of alcohol (F10·x)** | | | | | | | | | | |
| Bradbury 1993(3) | Alcohol intoxication | NR | Private vehicle occupants admitted to A&E | A&E | NR | England | 33 | 36·2 | 4 | 174 |
| Holt 1980(4) | Alcohol intoxication | Physician Assessment | Patients attending A&E | A&E | Mar 1979 - Nov 1979 | Scotland | NR | 37 | 8 | 702 |
| Jain 2012(5) | Alcohol intoxication | NR | Patients less than 18 years who presented to the emergency department with a decreased conscious level | A&E | Nov 2010 - Sep 2011 | England | NR | NR | 4 | 1132 |
| Johnson 2001(6) | Alcohol intoxication  Harmful use of alcohol  Alcohol dependence  Alcohol withdrawal state with delirium  Alcohol induced psychotic disorder | ICD-10 | Patients admitted to an acute psychogeriatiric unit | Mental health inpatient unit | Dec 1997 - Apr 1999 | England | NR | NR | 9 | 149 |
| Locker 2007(7) | Alcohol intoxication | NR | Patients attending A&E and a minor injury unit | A&E | Jan 2003 - Dec 2003 | England | NR | 46·2 | 7 | 75141 |
| Lockhart 1986(8) | Alcohol intoxication  Harmful use of alcohol  Alcohol withdrawal state  Alcoholic hepatitis  Alcoholic liver disease, unspecified | NR  ≥ 5 Brief MAST  NR  NR  NR | Patients with an emergency admission to a general medical firm | General medical or surgical ward | Nov 1983 and Jun 1984 | England | NR | NR | 8 | 104 |
| Mehta 2006(9) | Alcohol intoxication  Alcohol withdrawal state  Alcohol amnestic disorder  Alcoholic Liver Disease, Unspecified | ICD-10 | Inpatients aged > 60 years referred to the alcohol liaison nurse | General medical or surgical ward | Apr 1998 - Mar 2003 | England | NR | 32·7 | 7 | 193 |
| Rainer 1996(10) | Alcohol intoxication | ICD-9 | Patients admitted to an A&E observation ward | General medical or surgical ward | Jan 1992 - Dec 1992 | Scotland | 34 | 75 | 9 | 2460 |
| Trevett 1990(11) | Alcohol intoxication  Harmful use of alcohol | Blood alcohol level > 17·4 mmol/l  ≥ 5 MAST | Patients attending A&E | General medical or surgical ward | Dec 1988 - Mar 1989 | Scotland | NR | 0 | 6 | 522 |
| Vardy 2016(12) | Alcohol intoxication  Alcohol dependence | ICD-10 | Patients admitted to A&E | A&E | Sep 2012 - Dec 2012 | Scotland | 54·1 | 48·9 | 9 | 5497 |
| Wilkinson 1984(13) | Alcohol intoxication  Alcohol dependence | NR | Patients with a diagnosis of schizophrenia admitted to psychiatric hospital with parasuicide | Mental health inpatient unit | 1968 - 1981 | Scotland | NR | 48·7 | 4 | 343 |
| Zisman 2015(14) | Alcohol intoxication | Used either a breathalyser or blood alcohol content | Patients admitted under Section 136 of the Mental Health Act | Mental health inpatient unit | Feb 2012 - Jul 2012 | England | NR | 43·2 | 8 | 245 |
| Ball 2011(15) | Harmful use of alcohol  Alcohol dependence | ≥ 8 & < 20 AUDIT  ≥ 20 AUDIT | Medical patients | General medical or surgical ward | Jan 2010 - Mar 2010 | England | 70 | NR | 8 | 188 |
| Barnaby 2003(16) | Harmful use of alcohol  Alcohol dependence | ≥ 8 & < 15 AUDIT  ≥ 15 AUDIT | Patients newly admitted to six acute psychiatric wards | Mental health inpatient unit | NR | England | 41 | 47 | 7 | 200 |
| Barr 2004(17) | Harmful use of alcohol | NR | Patients presenting to A&E with deliberate self-harm for the first time | A&E | 1996 - 2000 | Wales | NR | 34·52 | 4 | 2417 |
| Burke 1976a(18) | Harmful use of alcohol | NR | Patients admitted for intentional self-poisoning whom were Irish | General medical or surgical ward | Jan 1969 - Dec 1972 | England | NR | NR | 2 | 258 |
| Burke 1976b(18) | Harmful use of alcohol | NR | Patients admitted for intentional self-poisoning whom were Asian | General medical or surgical ward | Jan 1969 - Dec 1972 | England | NR | NR | 1 | 52 |
| Cameron 2006(19) | Harmful use of alcohol | > 5 MAST | Patients admitted to the medical receiving unit | General medical or surgical ward | Dec 2002 - Mar 2003 | Scotland | NR | 49·2 | 8 | 850 |
| Canning 1999(20) | Harmful use of alcohol | ≥ 8 AUDIT or Physician Assessment | Patients admitted to medicine | General medical or surgical ward | Jan 1995 - Jan 1997 | England | NR | 47·9 | 8 | 2988 |
| Chalmers 2009(21) | Harmful use of alcohol | NR | Patients admitted with a primary diagnosis of community acquired pneumonia | General medical or surgical ward | Jan 2005 - Jan 2008 | Scotland | 66 | 51·8 | 5 | 1269 |
| Cherry 2016(22) | Harmful use of alcohol  Alcohol dependence | AUDIT-C | Patients over 18 attending A&E | A&E | NR | Northern Ireland | NR | NR | 7 | 1114 |
| Chick 1991(23) | Harmful use of alcohol | ≥ 2 study specific questionnaire | Patients admitted to an acute male orthopaedic ward | General medical or surgical ward | Sep 1987 - Nov 1987 | Scotland | NR | 0 | 3 | 369 |
| Craig 2011(24) | Harmful use of alcohol | An excess of 56 units/week for men, and 42 units/week for women | Patients with paracetamol induced hepatotoxicity admitted to the Scottish liver transplant unit | General medical or surgical ward | Nov 1992 - Oct 2008 | Scotland | NR | 52·5 | 4 | 581 |
| Dolman 2005(25) | Harmful use of alcohol | ≥ 8 AUDIT | Patients admitted to the acute medical ward | General medical or surgical ward | NR | Wales | 61·7 | 50·7 | 8 | 874 |
| Donoghue 2017(26) | Harmful use of alcohol | ≥ 3 AUDIT- C | Patients attending A&E aged 10-17 | A&E | Dec 2012 - May 2015 | England | 13·28 | 46·2 | 6 | 5576 |
| Drummond 2014(27) | Harmful use of alcohol | M-SASQ, FAST or SIPS-PAT | Patients attending A&E | A&E | Mar 2008 - Apr 2009 | England | NR | NR | 8 | 5899 |
| Dunn 2003(28) | Harmful use of alcohol | NR | Patients attending A&E with a closed head injury | A&E | Jul 1996 - Dec 2000 | Scotland | NR | NR | 5 | 24927 |
| Gwaspari 2011(29) | Harmful use of alcohol  Alcohol dependence | ≥ 8 & < 16 AUDIT  ≥ 16 AUDIT | African or Caribbean patients admitted to psychiatric hospital with a psychotic disorder | Mental health inpatient unit | Sep 2008 - May 2009 | England | NR | NR | 2 | 79 |
| Hawton 1993(30) | Harmful use of alcohol | NR | Patients aged 15-24 years admitted to the regional poisoning centre because of self-poisoning or self-injury | General medical or surgical ward | 1968 - 1985 | Scotland | NR | NR | 3 | 140 |
| Hawton 2008(31) | Harmful use of alcohol | Drinking > nationally recommended adult maximum weekly limits | Patients under 15 years old presenting with deliberate self-harm | General medical or surgical ward | Jan 1978 - Dec 2003 | England | NR | 86·8 | 2 | 404 |
| Hodgins 2007(32) | Harmful use of alcohol  Alcohol dependence | ≥ 8 & < 16 AUDIT: ♂  ≥ 6 & < 16 AUDIT: ♀  ≥ 16 AUDIT | Psychiatric inpatients with severe mental illness | Mental health inpatient unit | Jul 2004 - Apr 2005 | England | 38·4 | 41·4 | 5 | 205 |
| Howell 2016(33) | Harmful use of alcohol | NR | Inpatients with suspected bone, native joint or soft tissue infection | General medical or surgical ward | 2012 | England | 43 | 41·7 | 5 | 169 |
| Jarman 1979(34) | Harmful use of alcohol | ≥ 5 semi-structured drinking questionnaire(35) | Patients admitted to a general medical or orthopaedic ward or attending A&E | General medical or surgical ward |  | England | NR | 46·9 | 4 | 303 |
| Knightly 2016(36) | Harmful use of alcohol | ≥ 5 MAST-G | Inpatients aged 65 or over admitted to the acute medical unit | General medical or surgical ward | NR | England | 79 | 61 | 6 | 100 |
| Kouimtsidis 2003(37) | Harmful use of alcohol | ≥ 8 AUDIT | Patients admitted to the general hospital | General medical or surgical ward | 2000 | England | 53·2 | 56 | 8 | 264 |
| Lennox 1979(38) | Harmful use of alcohol | MAST or Physician Assessment | Patients admitted to the general medical unit | General medical or surgical ward | Jan 1977 - Mar 1977 | Scotland | NR | 100 | 6 | 328 |
| Lockhart 1987a(39) | Harmful use of alcohol | NR | Patients admitted for self-poisoning | General medical or surgical ward | Nov 1971 - Feb 1972 | England | NR | NR | 5 | 84 |
| Lockhart 1987b(39) | Harmful use of alcohol | NR | Patients admitted for self-poisoning | General medical or surgical ward | Sep 1983 - Jun 1984 | England | NR | NR | 5 | 87 |
| Lumsden 1998(40) | Harmful use of alcohol | A note of alcohol being a significant factor in the patient's presentation, with validation from independent sources | Patients admitted to a high security hospital | Mental health inpatient unit | Nov 1990 - Aug 1992 | England | 31·3 | 0 | 5 | 100 |
| Luttrell 1997(41) | Harmful use of alcohol | ≥ 5 MAST-G | Patients admitted as an emergency to two acute admission wards aged > 65 years | General medical or surgical ward | NR | England | 78 | 57 | 7 | 162 |
| MacKenzie 1996(42) | Harmful use of alcohol | ≥ 8 AUDIT | Patients admitted to the acute medical receiving ward | General medical or surgical ward | Feb 1995 - May 1995 | Scotland | NR | NR | 5 | 239 |
| Mangion 1992(43) | Harmful use of alcohol | Alcohol intake greater than 21 units/ week for men and 14 units/week for women and/or; ≥ 2 CAGE and/or; Elevated GGT (>41 IU/1) or MCV (> 98 fl) levels, where corroboration from informants revealed an excessive alcohol intake | Patients admitted to medicine | General medical or surgical ward | NR | England | 77·3 | 49 | 8 | 530 |
| Martin 1983(44) | Harmful use of alcohol | MAST and Physician Assessment | Patents admitted to the medical unit | General medical or surgical ward | Dec 1981 - Mar 1982 | Scotland | NR | 0 | 6 | 528 |
| McCloud 2004(45) | Harmful use of alcohol  Alcohol dependence | ≥ 8 AUDIT  ≥ 16 AUDIT | Patients admitted to psychiatric hospital | Mental health inpatient unit | Nov 2001 - Jun 2002 | England | NR | 47·2 | 8 | 199 |
| McCusker 2002(46) | Harmful use of alcohol | AUDIT | Patients admitted to general medicine | General medical or surgical ward | Jun 1997 - Feb 1998 | England | 56 | 49·5 | 7 | 103 |
| McPeake 2015(47) | Harmful use of alcohol  Alcohol dependence | ICD-10 | Patients admitted to ICU | ICU | Jun 2012 - Dec 2013 | Scotland | 57 | NR | 9 | 580 |
| McQueen 2015(48) | Harmful use of alcohol  Alcohol dependence | ≥ 3 & < 13 FAST  ≥ 13 FAST | Patients admitted to medical and orthopaedic wards | General medical or surgical ward | NR | Scotland | NR | NR | 6 | 2398 |
| Morgan 1975(49) | Harmful use of alcohol  Alcohol dependence  Alcohol induced psychotic disorder | Interviews with patients and the hospital notes | Patients attending A&E following deliberate self-harm | A&E | 1972 | England | NR | 66·2 | 7 | 337 |
| Muzaimi 2010(50) | Harmful use of alcohol | NR | Patients admitted to the stroke rehabilitation unit aged 15 - 45 | General medical or surgical ward | Jan 2003 - Jan 2009 | Wales | 37·2 | 47·6 | 4 | 21 |
| Ninkovic 2012(51) | Harmful use of alcohol  Alcohol dependence | ≥ 8 & < 20 AUDIT  ≥ 20 AUDIT | Inpatients | General medical or surgical ward | NR | England | 69 | NR | 8 | 380 |
| Oude Voshaar 2011(52) | Harmful use of alcohol | NR | Patients presenting to A&E as a result of deliberate self-harm | A&E | Sep 1997 - Aug 2007 | England | 48·2 | 50·4 | 7 | 2217 |
| Poole 2014(53) | Harmful use of alcohol | NR | Inpatients experiencing delayed discharge from a mental health hospital | Mental health inpatient unit | Oct 2009 and Jan 2010 | England | 48·1 | 50 | 4 | 79 |
| Sangha 2015(54) | Harmful use of alcohol | NR | Patients admitted with stroke | General medical or surgical ward | Jan 2000 - Dec 2007 | England | 71·5 | 47·5 | 7 | 4804 |
| Sharkey 1996a(55) | Harmful use of alcohol | ≥ 8 AUDIT | Inpatients | General medical or surgical ward | Nov 1994 - | Northern Ireland | 56 | 58 | 8 | 136 |
| Sharkey 1996b(55) | Harmful use of alcohol | ≥ 8 AUDIT | Patients attending A&E | A&E | Nov 1994 - | Northern Ireland | 36 | 43 | 8 | 104 |
| Sinclair 2008(56) | Harmful use of alcohol  Alcohol dependence | ≥ 8 & < 15 AUDIT  ≥ 15 AUDIT | Patients admitted to the acute adult wards of a psychiatric hospital | Mental health inpatient unit | Jul 2005 - Oct 2005 | England | NR | NR | 8 | 178 |
| Taylor 1986(57) | Harmful use of alcohol | ≥ 6 Brief MAST | Patients admitted as an emergency to medical and surgical wards | General medical or surgical ward | Oct 1984 - Jan 1985 | England | NR | NR | 7 | 1628 |
| Taylor 1998(58) | Harmful use of alcohol | NR | Inpatients in the special hospitals (i·e· high security patients with mental disorders) | Mental health inpatient unit | Jan 1993 - Jun 1993 | England | 39 | 17 | 7 | 1740 |
| Thom 1999(59) | Harmful use of alcohol | ≥ 8 AUDIT | Patients attending A&E | A&E | Mar 1996 - May 1996 | England | NR | 46·8 | 8 | 679 |
| Alavi 2016(60) | Alcohol dependence | NR | Patients with hepatitis C virus related liver disease admitted with decompensated cirrhosis | General medical or surgical ward | 2001 - 2014 | Scotland | NR | NR | 4 | 1222 |
| Barrison 1982(61) | Alcohol dependence | ≥ 2 CAGE or Consumption Index | Inpatients except paediatric, geriatric or two surgical wards | General medical or surgical ward | Sep 1980 and Dec 1980 | England | NR | NR | 7 | 520 |
| Ben-Shlomo 1992(62) | Alcohol dependence | ≥ 2 CAGE | Patients admitted to three adjoining hospitals | General medical or surgical ward | NR | England | NR | NR | 5 | 105 |
| Bernadt 1986(63) | Alcohol dependence | 25 questions relating to alcohol consumption | Patients admitted to psychiatric hospital | Mental health inpatient unit | Feb 1980 - Nov 1980 | England | NR | 50·1 | 7 | 371 |
| Bruce 2014(64) | Alcohol dependence | ≥ 16 AUDIT | Patients with serious mental illness of black Caribbean, black African, and white British ethnicity | Mental health inpatient unit | Sep 2008 - Jul 2010 | England | 37·4 | 0 | 3 | 165 |
| Carney 1995(65) | Alcohol dependence | ICD-9 | Patients admitted to psychiatric hospital | Mental health inpatient unit | Jun 1987 -Jun 1990 | England | NR | 54 | 9 | 1048 |
| Corbbett 1998a(66) | Alcohol dependence | Consultant diagnosis | Caucasian patients with schizophrenia convicted of serious offences | Mental health inpatient unit | 1972 - 1995 | England | NR | NR | 5 | 1111 |
| Corbbett 1998b(66) | Alcohol dependence | Consultant diagnosis | Caucasian patients with personality disorders convicted of serious offences | Mental health inpatient unit | 1972 - 1995 | England | NR | NR | 5 | 885 |
| Feldman 1986(67) | Alcohol dependence | ≥ 2 CAGE | Patients admitted to three general medical firms | General medical or surgical ward | Oct 1983 - Dec 1983 | England | NR | NR | 8 | 382 |
| Forrest 1973(68) | Alcohol dependence | NR | Patients admitted to the regional poisoning treatment centre having taken lysergide | General medical or surgical ward | Jan 1971 - Jul 1973 | Scotland | 20 | 33·3 | 4 | 60 |
| Franklin 1977(69) | Alcohol dependence | NR | Doctors admitted to a private psychiatric hospital | Mental health inpatient unit | Jan 1965 - Dec 1974 | England | NR | NR | 3 | 100 |
| Glass 1988(70) | Alcohol dependence  Alcohol induced psychotic disorder | ICD-8 | Patients admitted to psychiatric hospital | Mental health inpatient unit | 1970-1981 | England | NR | NR | 9 | 43552 |
| Hall 1995(71) | Alcohol dependence | NR | Patients detained under section 5(2) of the Mental Health Act in psychiatric hospital | Psychiatric Hospital | Apr 1992 - Mar 1993 | England | NR | 57 | 4 | 61 |
| Hamlyn 1978(72) | Alcohol dependence | NR | Patients admitted to hospital with paracetamol overdose | General medical or surgical ward | Nov 1974 - Nov 1975 | England | NR | NR | 6 | 201 |
| Herzberg 1987(73) | Alcohol dependence  Alcoholic gastritis | NR | Patients admitted to psychiatric hospital who were homeless upon admission | Mental health inpatient unit | 1971 - 1980 | England | NR | 22·6 | 6 | 110 |
| Holmes 2010(74) | Alcohol dependence | NR | All patients admitted with burns to a regional burns centre | General medical or surgical ward | 2003 - 2008 | England | NR | NR | 5 | 1293 |
| Kelleher 1975(75) | Alcohol dependence  Alcohol withdrawal state with delirium  Alcohol induced psychotic disorder | NR | Patients admitted to psychiatric hospital | Mental health inpatient unit | NR | England | NR | NR | 4 | 174 |
| Laugharne 1997(76) | Alcohol dependence | ICD-10 | Inpatients referred to liaison psychiatry | General medical or surgical ward | NR | England | NR | NR | 9 | 94 |
| MacIntyre 1979(77) | Alcohol dependence  Alcohol withdrawal state with delirium  Alcoholic cirrhosis of liver  Alcoholic polyneuropathy | MAST | Patients admitted as emergencies to a general medical ward | General medical or surgical ward | 1977 | Scotland | NR | 0 | 5 | 301 |
| Maguire 1974(78) | Alcohol dependence | NR | Patients admitted to two medical wards | General medical or surgical ward | Nov 1971 - Dec 1971 | England | NR | 52·1 | 7 | 230 |
| Mangan 1994(79) | Alcohol dependence | ≥ 2 CAGE | Inpatient in a rural hospital | General medical or surgical ward | Mar 1993 - | Northern Ireland | NR | 59·3 | 5 | 91 |
| Merrill 1986(80) | Alcohol dependence | NR | Patients admitted following deliberate self-poisoning | General medical or surgical ward | Jan 1979 - Dec 1981 | England | 30·3 | 63 | 7 | 1160 |
| Merrill 1992(81) | Alcohol dependence | NR | English-born patients whom had attempted suicide admitted to the West Midlands Poisons Unit | General medical or surgical ward | Apr 1988 - | England | 33·4 | 59·2 | 5 | 250 |
| Orford 1992(82) | Alcohol dependence | ≥ 2 CAGE | Patients who were acute admissions to medical wards | General medical or surgical ward | NR | England | 56·6 | 39·1 | 8 | 546 |
| Peters 2002(83) | Alcohol dependence | ≥ 2 CAGE | Patients attending A&E | A&E | Oct 1995 - Mar 1996 | England | NR | NR | 5 | 3381 |
| Platt 1991(84) | Alcohol dependence | NR | Patients presenting with parasuicide treated at the regional poisoning treatment centre | General medical or surgical ward | 1968 - 1987 | Scotland | NR | 58·7 | 7 | 5120 |
| Saxena 2000(85) | Alcohol dependence | ICD-10 | Patients admitted to hospital | General medical or surgical ward | Apr 1992 - Sep 1998 | England | NR | NR | 6 | 530 |
| Schoepf 2015(86) | Alcohol dependence  Alcoholic gastritis  Alcoholic hepatic failure  Alcoholic liver disease, unspecified  Alcohol-induced acute pancreatitis  Alcohol-induced chronic pancreatitis | ICD-10 | Patients admitted to hospital | General medical or surgical ward | Jan 2000 - Jun 2012 | England | NR | NR | 9 | 929456 |
| Van der Pol 1996(87) | Alcohol dependence | CAGE | Patients attending A&E | A&E | NR | England | 77 | 74·2 | 6 | 105 |
| Husain 2013(88) | Alcohol dependence  Alcohol withdrawal state  Alcoholic liver disease, unspecified | Examination of the case notes | Patients admitted to medicine | General medical or surgical ward | Jan 2010 - Dec 2011 | England | NR | NR | 7 | 48200 |
| Dowey 1993(89) | Alcohol withdrawal state with delirium | NR | Patients attending A&E | A&E | NR | Northern Ireland | NR | 48·8 | 6 | 6625 |
| Ramakrishna 2012(90) | Alcohol induced amnestic disorder | ICD-10 | Inpatients with acquired brain injury in the medium and low secure neurorehabilitation units | Mental health inpatient unit | Aug 2011 - | England | 41·2 | 0 | 6 | 43 |
| Clark 2011(91) | Intentional self-poisoning by and exposure to alcohol | NR | All patients admitted to ICU | ICU | Jan 2005 - Dec 2009 | Scotland | NR | NR | 7 | 12702 |
| **Poisoning due to alcohol** | | | | | | | | | | |
| Lawson 1983(92) | Poisoning by and exposure to alcohol, undetermined intent | NR | All patients admitted to hospital under the aged 10- 15 with a diagnosis of poisoning | General medical or surgical ward | 1974 - 1981 | England | NR | NR | 5 | 417 |
| **Liver disorders due to alcohol K70·x** | | | | | | | | | | |
| Bretherick 2011(93) | Alcoholic hepatitis | NR | Patients admitted to the Scottish liver transplant unit | General medical or surgical ward | Nov 1992 - Mar 2009 | Scotland | 38·1 | NR | 5 | 949 |
| Johnston 1991(94) | Alcoholic fibrosis and sclerosis of liver | NR | Patients admitted with Child’s Class C liver disease with acute variceal bleeding | General medical or surgical ward | Jan 1980 - Dec 1989 | Northern Ireland | 55 | 45·1 | 5 | 102 |
| Ahmadnia 2015(95) | Alcoholic cirrhosis of liver | NR | Patients with decompensated chronic liver disease | ICU | Jan 2012 - Dec 2013 | England | 57 | 38 | 3 | 37 |
| Berry 2012(96) | Alcoholic cirrhosis of liver  Alcoholic hepatitis | NR | Patients admitted with decompensated cirrhosis | General medical or surgical ward | Nov 2010 - Nov 2011 | England | NR | NR | 6 | 66 |
| Cole 2016(97) | Alcoholic cirrhosis of liver | NR | Patients discharged from the hepatology ward | General medical or surgical ward | Jan 2013 - Dec 2013 | Scotland | 56 | 34·8 | 4 | 198 |
| Dyson 2016(98) | Alcoholic cirrhosis of liver | NR | Patients with decompensated cirrhosis | General medical or surgical ward | Nov 2013 - Mar 2014 | England | 53 | 41 | 2 | 228 |
| Emerson 2014(99) | Alcoholic cirrhosis of liver | NR | Patients admitted to ICU with cirrhosis | ICU | Jun 2012 - May 2013 | Scotland | 51 | 32 | 4 | 59 |
| Hampshire 2014(100) | Alcoholic cirrhosis of liver | NR | Patients admitted to ICU | ICU | Jul 2003 - Sep 2011 | England | NR | NR | 7 | 4178 |
| Lloyd-Evans 2015(101) | Alcoholic cirrhosis of liver | NR | Patients admitted to critical care | ICU | 2009 - 2014 | Wales | NR | · | 6 | 5394 |
| Musumba 2013(102) | Alcoholic cirrhosis of liver | NR | Patients admitted to ICU | ICU | Jul 2003 - Sep 2011 | England | NR | NR | 7 | 4178 |
| Shawcross 2012(103) | Alcoholic cirrhosis of liver  Alcoholic hepatitis | NR | Patients admitted to a liver ICU with cirrhosis and organ dysfunction | ICU | Jan 2000 - Jun 2007 | England | NR | NR | 7 | 563 |
| Thomson 2010(104) | Alcoholic cirrhosis of liver | NR | Patients admitted to ICU | ICU | Oct 2007 - Jul 2009 | England | NR | NR | 7 | 4198 |
| Al-Freah 2010(105) | Alcoholic Liver Disease, Unspecified | NR | Patients admitted to the liver intensive therapy unit with severe upper gastrointestinal variceal bleeding | ICU | Jan 2000 - Mar 2008 | England | 49 | 42·7 | 8 | 157 |
| Bugeja 2012(106) | Alcoholic Liver Disease, Unspecified | NR | Patients diagnosed with spontaneous bacterial peritonitis whom were admitted with ascites to the liver unit | General medical or surgical ward | 2009 | England | NR | NR | 3 | 26 |
| Burke 2017(107) | Alcoholic Liver Disease, Unspecified | NR | Patients with decompensated chronic liver disease | General medical or surgical ward | Apr 2014 - Jun 2015 | England | NR | NR | 3 | 117 |
| Butler 2001(108) | Alcoholic Liver Disease, Unspecified  Alcohol withdrawal state | NR | Patients admitted to the acute medical receiving unit | General medical or surgical ward | NR | Scotland | NR | NR | 7 | 360 |
| Corbett 2012(109) | Alcoholic Liver Disease, Unspecified | NR | Patients with a history of variceal bleeding admitted to an intensive care unit | ICU | NR | England | 53·7 | NR | 3 | 41 |
| Davies 1992(110) | Alcoholic Liver Disease, Unspecified | NR | Patients admitted with liver disease | General medical or surgical ward | Jan 1987 - Dec 1989 | Wales | NR | NR | 4 | 74 |
| Docking 2014(111) | Alcoholic Liver Disease, Unspecified | NR | Patients admitted to the intensive care unit | ICU | Oct 2008 - Nov 2010 | Scotland | NR | 39·8 | 7 | 1029 |
| Dsouza 2015(112) | Alcoholic Liver Disease, Unspecified | NR | Patients admitted for a liver transplant assessment | General medical or surgical ward | Feb 2012 - Mar 2013 | England | NR | NR | 6 | 109 |
| Hislop 2004(113) | Alcoholic Liver Disease, Unspecified | NR | Inpatients in general medical and gastroenterology wards | General medical or surgical ward | 2000 - 2001 | Scotland | NR | 51·5 | 6 | 1637 |
| Lewis 2003(114) | Alcoholic Liver Disease, Unspecified | NR | Patients who received liver transplants | General medical or surgical ward | Sep 1990 - Aug 2000 | England | 46·9 | 40 | 7 | 627 |
| McPhail 2018(115) | Alcoholic Liver Disease, Unspecified | ARLD was identified where primary, secondary, or ultimate primary reason for admission was either alcohol withdrawal seizures, alcoholic cirrhosis, acute alcoholic hepatitis, alcohol-induced chronic pancreatitis, self-poisoning with alcohol, alcohol overdose, delirium tremens or alcohol dependence | Patients with cirrhosis admitted to ICU | ICU | Jan 1998 - Dec 2012 | United Kingdom (constituent nation prevalence not reported) | 52·7 | 38·5 | 8 | 31363 |
| Welch 2008(116) | Alcoholic Liver Disease, Unspecified | NR | Patients admitted to ICU | ICU | Dec 1995 - July 2005 | United Kingdom (constituent nation prevalence not reported) | NR | NR | 7 | 385429 |
| **Gastrointestinal disorders due to alcohol** | | | | | | | | | | |
| Ellis 2009(117) | Alcohol-induced acute pancreatitis | Information in the clinical records | Patients admitted with acute pancreatitis | General medical or surgical ward | Sep 2006 - Mar 2007 | England | 57 | 47·4 | 5 | 963 |
| Giggs 1998(118) | Alcohol-induced acute pancreatitis | NR | Patients admitted with primary acute pancreatitis | General medical or surgical ward | 1969 - 1983 | England | NR | 54·2 | 4 | 498 |
| Imrie 1975(119) | Alcohol-induced acute pancreatitis | NR | Patients admitted with acute pancreatitis | General medical or surgical ward | 1971 - 1972 | Scotland | NR | 53·8 | 6 | 78 |
| Lowham 1999(120) | Alcohol-induced acute pancreatitis | NR | Patients admitted with a diagnosis of acute pancreatitis | General medical or surgical ward | Jan 1996 - Dec 1997 | England | 62·3 | 46·7 | 7 | 105 |
| O'Reilly 2017(121) | Alcohol-induced acute pancreatitis | NR | Patients admitted with severe acute pancreatitis | General medical or surgical ward | Jan 2014 - Jun 2014 | United Kingdom (constituent Nation prevalence not reported) | NR | NR | 6 | 692 |
| Pavlidis 2012(122) | Alcohol-induced acute pancreatitis | NR | Patients with severe acute pancreatitis admitted to ICU | ICU | Jan 2005 - Dec 2010 | England | NR | NR | 4 | 50 |
| Read 1976(123) | Alcohol-induced acute pancreatitis Alcohol-induced chronic pancreatitis | NR | Patients admitted with exocrine pancreatic disease | General medical or surgical ward | 1968 - 1974 | England | NR | 46·2 | 7 | 119 |
| Toh 2000(124) | Alcohol-induced acute pancreatitis | NR | Patients admitted with acute pancreatitis | General medical or surgical ward | Dec 1994 - Nov 1995 | England | 54·3 | 43 | 7 | 186 |
| Howat 1968(125) | Alcohol-induced chronic pancreatitis | NR | Patients admitted with chronic pancreatitis | General medical or surgical ward | NR | England | NR | 51·9 | 3 | 54 |
| **Other disorders due to alcohol** | | | | | | | | | | |
| Peters 1985(126) | Alcoholic myopathy | Quadriceps muscle biopsy | Patients admitted to a district general hospital for assessment and alcohol re-education | General medical or surgical ward | NR | England | 47 | 30·4 | 6 | 151 |

^NR=Not reported; NOS=Newcatsle-Ottawa Scale; ICD=International Classification of Disease; DSM=Diagnostic and Statistical Manual of Mental Disorders; A&E=Accident and Emergency; ICU=In tensive Care Unit; AUDIT= Alcohol Use Disorders Identification Test; CAGE=CAGE Questionnaire; MAST= Michigan Alcohol Screening Test; M-SASQ=Modified Single Alcohol Screening Question; FAST=Fast Alcohol Screening Test; SIPS-PAT=Screening and Intervention Programme for Sensible drinking Paddington Alcohol Test; MCV=Mean Corpuscular Volume; GGT=Gamma-Glutamyl Transferase; ARLD=Alcohol Related Liver Disease^

**Table S3: GRADE clinical evidence profile for overall prevalence estimates in non-selective patients**

| **Quality Assessment** | | | | | | **Prevalence %  (95% CI)** | **Quality** |
| --- | --- | --- | --- | --- | --- | --- | --- |
| **No of prevalence estimates** | **Risk of bias** | **Inconsistency** | **Indirectness** | **Imprecision** | **Publication Bias** |  |  |
| Alcohol intoxication F10.0 | | | | | | | |
| 5 | None | Very Serious | None | Very Serious | N/A | 8.99  (0.58 -25.38) | VERY LOW |
| Harmful use of alcohol F10.1 | | | | | | | |
| 29 | None | Very Serious | None | None | None | 19.76  (15.61 - 24.26) | VERY LOW |
| Alcohol dependence F10.2 | | | | | | | |
| 23 | None | Very Serious | None | None | None | 10.25  (7.06 - 13.96) | VERY LOW |
| Alcohol withdrawal state F10.3 | | | | | | | |
| 3 | None | Very Serious | None | None | N/A | 1.17  (0.03 - 3.47) | VERY LOW |
| Alcohol withdrawal state with delirium F10.4 | | | | | | | |
| 3 | Serious | Very Serious | None | None | N/A | 0.28  (0.00 - 1.68) | VERY LOW |
| Alcohol induced psychotic disorder F10.5 | | | | | | | |
| 2 | Serious | N/A | None | None | N/A | 0.04  (0.02 - 0.08) | VERY LOW |
| Intentional self-poisoning by and exposure to alcohol X65 | | | | | | | |
| 1 | None | N/A | None | None | N/A | 1.54  (1.34 - 1.76) | LOW |
| Alcoholic hepatitis K70.1 | | | | | | | |
| 1 | None | N/A | None | None | N/A | 0.96  (0.17 - 5.25) | LOW |
| Alcoholic cirrhosis of liver K70.3 | | | | | | | |
| 5 | Serious | Very Serious | None | None | N/A | 2.23  (1.77 - 2.74) | VERY LOW |
| Alcoholic hepatic failure K70.4 | | | | | | | |
| 1 | Serious | N/A | None | None | N/A | 0.22  (0.20 - 0.24) | VERY LOW |
| Alcoholic liver disease, unspecified K70.9 | | | | | | | |
| 5 | Serious | Very Serious | None | None | N/A | 2.01  (1.52 - 2.56) | VERY LOW |
| Alcohol-induced acute pancreatitis K85.2 | | | | | | | |
| 1 | Serious | N/A | None | None | N/A | 0.41  (0.39 - 0.44) | VERY LOW |
| Alcohol-induced chronic pancreatitis K86.0 | | | | | | | |
| 1 | Serious | N/A | None | None | N/A | 0.41  (0.39 - 0.44) | VERY LOW |
| Alcoholic gastritis K29.2 | | | | | | | |
| 1 | Serious | N/A | None | None | N/A | 2.15  (2.09 - 2.20) | VERY LOW |
| Alcoholic polyneuropathy G62.1 | | | | | | | |
| 1 | Serious | N/A | None | None | N/A | 1.00  (0.34 - 2.89) | VERY LOW |

**Figure S8: Forest plots of meta-analysis for pooled prevalence of wholly attributable alcohol conditions in non-selective patients in the UK hospital system**

**Table S4: Pooled prevalence for wholly attributable alcohol conditions in patients with an alcohol diagnosis in the UK hospital system**

| **Patients with an alcohol diagnosis** | **Patient cohort studied** | **Number of Prevalence Estimates (n)** | **Prevalence % (95% CI)** |
| --- | --- | --- | --- |
| **Mental and behavioural disorders due to use of alcohol (F10.x)** |  | | |
| Alcohol dependence F10.2 | Patients with alcohol withdrawal syndrome | 1 | 77.25 (71.14 - 82.39)* |
| **Liver disorders due to alcohol K70.x** |  | | |
| Alcoholic liver disease, unspecified K70.9 | Patients with alcohol withdrawal syndrome | 1 | 21.80 (16.76 - 27.85) |
| **Other disorders due to alcohol** |  | | |
| Alcoholic myopathy G72.1 | Patients in a district general hospital admitted for alcohol re-education | 1 | 59.60 (51.63 - 67.10) |

^All estimates were deemed^ *^very low^* ^quality according to GRADE unless marked by * which indicates^ *^low^* ^quality^

**Table S5: GRADE clinical evidence profile for wholly attributable alcohol conditions in patients with an alcohol diagnosis in the UK hospital system**

| **Quality Assessment** | | | | | | **Prevalence %  (95% CI)** | **Quality** |
| --- | --- | --- | --- | --- | --- | --- | --- |
| **No of prevalence estimates** | **Risk of bias** | **Inconsistency** | **Indirectness** | **Imprecision** | **Publication Bias** |  |  |
| Alcohol dependence F10.2 in patients with alcohol withdrawal syndrome | | | | | | | |
| 1 | None | N/A | None | None | N/A | 77.25  (71.14 - 82.39) | LOW |
| Alcoholic liver disease, unspecified K70.9 in patients with alcohol withdrawal syndrome | | | | | | | |
| 1 | None | N/A | None | Serious | N/A | 21.80  (16.76 - 27.85) | VERY LOW |
| Alcoholic myopathy G72.1 Patients in a district general hospital admitted for alcohol re-education | | | | | | | |
| 1 | Serious | N/A | None | Serious | N/A | 59.60  (51.63 - 67.10) | VERY LOW |

**Table S6: Pooled prevalence for wholly attributable alcohol conditions in patients with specific health disorders in the UK hospital system**

| **Patients with specific health disorders** |  | | **Prevalence % (95% CI)** | | | | |
| --- | --- | --- | --- | --- | --- | --- | --- |
|  | **Patient cohort studied** | **Number of Prevalence Estimates (n)** | **General medical or surgical ward** | **ICU** | **A&E** | **Mental health inpatient unit** | **Overall** |
| **Mental and behavioural disorders due to use of alcohol (F10.x)** | | | | | | | |
| Alcohol Intoxication F10.0 | Patients referred to the alcohol liaison nurse | 1 | 17.62 (12.89 - 23.61) | - | - | - | 17.62 (12.89 - 23.61) |
|  | Patients admitted to an A&E observation ward | 1 | 15.89 (14.50 - 17.39) | - | - | - | 15.89 (14.50 - 17.39)* |
|  | Patients who presented with a decreased conscious level | 1 | - | - | 27.83 (25.29 - 30.51) | - | 27.83 (25.29 - 30.51) |
|  | Patients who were private vehicle occupants | 1 | - | - | 5.75 (3.15 - 10.25) | - | 5.75 (3.15 - 10.25) |
|  | Patients with a diagnosis of schizophrenia admitted with parasuicide | 1 | - | - | - | 8.16 (5.71 - 11.55) | 8.16 (5.71 - 11.55) |
|  | Patients admitted under Section 136 of the Mental Health Act | 1 | - | - | - | 38.37 (32.50 - 44.59) | 38.37 (32.50 - 44.59) |
| Harmful use of alcohol F10.1 | Patients with deliberate self-harm | 4 | 8.64 (6.39 - 11.18) | - | 30.81 (29.10 - 32.55) | - | 16.14 (3.07 -36.54) |
|  | Patients with self-poisoning | 2 | 2.50 (1.01 - 4.54) | - | - | - | 2.50 (1.01 - 4.54) |
|  | Patients with a psychotic disorder | 1 | - | - | - | 15.19 (8.91 - 24.70) | 15.19 (8.91 - 24.70) |
|  | Patients with a severe mental illness | 1 | - | - | - | 33.66 (27.54 - 40.38) | 33.66 (27.54 - 40.38) |
|  | Patients experiencing a delayed discharge | 1 | - | - | - | 43.04 (32.69 - 54.03) | 43.04 (32.69 - 54.03) |
|  | Patients with a closed head injury | 1 | - | - | 17.64 (17.17 - 18.12) | - | 17.64 (17.17 - 18.12) |
|  | Patients with suspected bone, native joint or soft tissue infection | 1 | - | - | 11.24 (7.32 - 16.89) | - | 11.24 (7.32 - 16.89) |
|  | Patients with paracetamol induced hepatotoxicity | 1 | 45.27 (41.26 - 49.33) | - | - | - | 45.27 (41.26 - 49.33) |
|  | Patients with stroke | 1 | 2.27 (1.88 - 2.73) | - | - | - | 2.27 (1.88 - 2.73)* |
|  | Patients with community acquired pneumonia | 1 | 5.52 (4.39 - 6.91) | - | - | - | 5.52 (4.39 - 6.91) |
| Alcohol dependence F10.2 | Patients who are homeless | 1 | - | - | - | 23.64 (16.67 - 32.38) | 23.64 (16.67 - 32.38) |
|  | Patients who are doctors | 1 | - | - | - | 20.00 (13.34 - 28.88) | 20.00 (13.34 - 28.88) |
|  | Patients with schizophrenia convicted of serious offences | 1 | - | - | - | 2.34 (1.60 - 3.41) | 2.34 (1.60 - 3.41) |
|  | Patients with a psychotic disorder | 1 | - | - | - | 18.99 (11.86 - 28.99) | 18.99 (11.86 - 28.99) |
|  | Patients with schizophrenia admitted with parasuicide | 1 | - | - | - | 1.46 (0.62 - 3.37) | 1.46 (0.62 - 3.37) |
|  | Patients with serious mental illness | 2 | - | - | - | 16.76 (13.10 - 20.76) | 16.76 (13.10 - 20.76) |
|  | Patients detained under section 5(2) of the Mental Health Act | 1 | - | - | - | 3.28 (0.90 - 11.19) | 3.28 (0.90 - 11.19) |
|  | Patients with personality disorders convicted of serious offences | 1 | - | - | - | 6.44 (5.00 - 8.25) | 6.44 (5.00 - 8.25) |
|  | Patients with deliberate self-harm | 3 | 9.77 (8.99 - 10.59) | - | 10.39 (7.56 - 14.10) | - | 11.17 (8.35 - 14.32) |
|  | Patients with hepatitis C virus related decompensated cirrhosis | 1 | 50.98 (48.18 - 53.78) | - | - | - | 50.98 (48.18 - 53.78) |
|  | Patients with self-poisoning | 1 | 6.12 (4.88 - 7.65) | - | - | - | 6.12 (4.88 - 7.65) |
|  | Patients unless they were admitted with an acute general surgical condition, a social problem, an overdose, or mental illness | 1 | 16.19 (10.36 - 24.41) | - | - | - | 16.19 (10.36 - 24.41) |
|  | Patients whom were poisoned by lysergide | 1 | 5.00 (1.71 - 13.70) | - | - | - | 5.00 (1.71 - 13.70) |
|  | Patients with paracetamol overdose | 1 | 9.95 (6.53 - 14.87) | - | - | - | 9.95 (6.53 - 14.87) |
|  | Patients with burns | 1 | 12.30 (10.62 - 14.20) | - | - | - | 12.30 (10.62 - 14.20) |
| Alcohol withdrawal state F10.3 | Patients referred to the alcohol liaison nurse | 1 | 9.33 (5.98 - 14.26) | - | - | - | 9.33 (5.98 - 14.26)* |
| Alcohol induced psychotic disorder F10.5 | Patients with deliberate self-harm | 1 | - | - | 8.31 (5.81 - 11.75) | - | 8.31 (5.81 - 11.75)* |
| Alcohol induced amnestic disorder F10.6 | Patients referred to the alcohol liaison nurse | 1 | 0.52 (0.09 - 2.88) | - | - | - | 0.52 (0.09 - 2.88)* |
|  | Patients with acquired brain injury | 1 | - | - | - | 2.33 (0.41 - 12.06) | 2.33 (0.41 - 12.06) |
| **Poisoning due to alcohol** | | | | | | | |
| Poisoning by and exposure to alcohol, undetermined intent Y15 | Patients with a diagnosis of poisoning | 1 | 21.34 (17.68 - 25.53) | - | - | - | 21.34 (17.68 - 25.53) |
| **Liver disorders due to alcohol K70.x** | | | | | | | |
| Alcoholic hepatitis K70.1 | Patients with decompensated cirrhosis | 2 | 36.36 (25.81 - 48.42) | 7.99 (6.03 - 10.53) | - | - | 9.93 (7.67 - 12.44) |
|  | Patients admitted to a liver transplant unit | 1 | 0.95 (0.50 - 1.79) | - | - | - | 0.95 (0.50 - 1.79) |
| Alcoholic fibrosis and sclerosis of liver K70.2 | Patients with Child’s Class C liver disease with acute variceal bleeding | 1 | 51.96 (42.37 - 61.41) | - | - | - | 51.96 (42.37 - 61.41) |
| Alcoholic cirrhosis of liver K70.3 | Patients with decompensated cirrhosis | 5 | 84.48 (80.06 - 88.46) | 67.27 (41.50 - 88.54) | - | - | 74.39 (52.82 - 91.16) |
| Alcoholic liver disease, unspecified K70.9 | Patients with chronic liver disease | 3 | 63.44 (56.43 - 70.17) | 34.87 (34.34 - 35.40) | - | - | 52.70 (29.55 - 75.25) |
|  | Patients who received liver transplants | 1 | 17.38 (14.62 -20.55) | - | - | - | 17.38 (14.62 -20.55) |
|  | Patients with spontaneous bacterial peritonitis in those admitted with ascites | 1 | 65.38 (46.22 - 80.59) | - | - | - | 65.38 (46.22 - 80.59) |
|  | Patients referred to the alcohol liaison nurse | 1 | 10.88 (7.23 - 16.06) | - | - | - | 10.88 (7.23 - 16.06) |
|  | Patients admitted for a liver transplant assessment | 1 | 33.03 (24.91 - 42.30) | - | - | - | 33.03 (24.91 - 42.30) |
|  | Patients with a history of variceal bleeding | 1 | - | 56.10 (41.04 - 70.11) | - | - | 56.10 (41.04 - 70.11) |
|  | Patients with severe upper gastrointestinal variceal bleeding | 1 | - | 52.87 (45.08 - 60.51) | - | - | 52.87 (45.08 - 60.51) |
| **Gastrointestinal disorders due to alcohol** | | | | | | | |
| Alcohol-induced acute pancreatitis K85.2 | Patients with acute pancreatitis | 7 | 21.77 (15.67 - 28.56) | 40.00 (27.61 - 53.82) | - | - | 23.55 (17.39 - 30.32) |
|  | Patients with exocrine pancreatic disease | 1 | 6.72 (3.45 - 12.71) | - | - | - | 6.72 (3.45 - 12.71) |
| Alcohol-induced chronic pancreatitis K86.0 | Patients with chronic pancreatitis | 1 | 20.37 (11.77 - 32.90) | - | - | - | 20.37 (11.77 - 32.90) |
|  | Patients with exocrine pancreatic disease | 1 | 14.29 (9.11 - 21.69) | - | - | - | 14.29 (9.11 - 21.69) |
| Alcoholic gastritis K29.2 | Patients who are homeless | 1 | - | - | - | 0.91 (0.16 - 4.97) | 0.91 (0.16 - 4.97) |

^CI=Confidence Interval; A&E=Accident and Emergency; ICU=In tensive Care Unit^

**Table S7: GRADE clinical evidence profile for wholly attributable alcohol conditions in patients with specific health disorders in the UK hospital system**

| **Quality Assessment** | | | | | | **Prevalence %  (95% CI)** | **Quality** |
| --- | --- | --- | --- | --- | --- | --- | --- |
| **No of prevalence estimates** | **Risk of bias** | **Inconsistency** | **Indirectness** | **Imprecision** | **Publication Bias** |  |  |
| Alcohol Intoxication F10.0 in patients referred to the alcohol liaison nurse | | | | | | | |
| 1 | None | N/A | None | Serious | N/A | 17.62  (12.89 - 23.61) | VERY LOW |
| Alcohol Intoxication F10.0 in patients admitted to an A&E observation ward | | | | | | | |
| 1 | None | N/A | None | None | N/A | 15.89  (14.50 - 17.39) | LOW |
| Alcohol Intoxication F10.0 in patients who presented with a decreased conscious level | | | | | | | |
| 1 | Serious | N/A | None | None | N/A | 27.83  (25.29 - 30.51) | VERY LOW |
| Alcohol Intoxication F10.0 in patients who were private vehicle occupants | | | | | | | |
| 1 | Serious | N/A | None | None | N/A | 5.75  (3.15 - 10.25) | VERY LOW |
| Alcohol Intoxication F10.0 in patients with a diagnosis of schizophrenia admitted with parasuicide | | | | | | | |
| 1 | Serious | N/A | None | None | N/A | 8.16  (5.71 - 11.55) | VERY LOW |
| Alcohol Intoxication F10.0 in patients admitted under Section 136 of the Mental Health Act | | | | | | | |
| 1 | None | N/A | None | Serious | N/A | 38.37  (32.50 - 44.59) | VERY LOW |
| Harmful use of alcohol F10.1 in patients with deliberate self-harm | | | | | | | |
| 4 | Serious | Very Serious | None | Very Serious | N/A | 16.14  (3.07 - 36.54) | VERY LOW |
| Harmful use of alcohol F10.1 in patients with self-poisoning | | | | | | | |
| 2 | Serious | N/A | None | None | N/A | 2.50  (1.01 - 4.54) | VERY LOW |
| Harmful use of alcohol F10.1 in patients with a psychotic disorder | | | | | | | |
| 1 | Very Serious | N/A | None | Serious | N/A | 15.19  (8.91 - 24.70) | VERY LOW |
| Harmful use of alcohol F10.1 in patients with a severe mental illness | | | | | | | |
| 1 | Serious | N/A | None | Serious | N/A | 33.66  (27.54 - 40.38) | VERY LOW |
| Harmful use of alcohol F10.1 in patients experiencing a delayed discharge | | | | | | | |
| 1 | Serious | N/A | None | Very serious | N/A | 43.04  (32.69 - 54.03) | VERY LOW |
| Harmful use of alcohol F10.1 in patients with a closed head injury | | | | | | | |
| 1 | Serious | N/A | None | None | N/A | 17.64  (17.17 - 18.12) | VERY LOW |
| Harmful use of alcohol F10.1 in patients with suspected bone, native joint or soft tissue infection | | | | | | | |
| 1 | Serious | N/A | None | Serious | N/A | 11.24  (7.32 - 16.89) | VERY LOW |
| Harmful use of alcohol F10.1 in patients with paracetamol induced hepatotoxicity | | | | | | | |
| 1 | Serious | N/A | None | None | N/A | 45.27  (41.26 - 49.33) | VERY LOW |
| Harmful use of alcohol F10.1 in patients with stroke | | | | | | | |
| 1 | None | N/A | None | None | N/A | 2.27  (1.88 - 2.73) | LOW |
| Harmful use of alcohol F10.1 in patients with community acquired pneumonia | | | | | | | |
| 1 | Serious | N/A | None | None | N/A | 5.52  (4.39 - 6.91) | VERY LOW |
| Alcohol dependence F10.2 in patients who are homeless | | | | | | | |
| 1 | Serious | N/A | None | Serious | N/A | 23.64  (16.67 - 32.38) | VERY LOW |
| Alcohol dependence F10.2 in patients who are doctors | | | | | | | |
| 1 | Very Serious | N/A | None | Serious | N/A | 20.00  (13.34 - 28.88) | VERY LOW |
| Alcohol dependence F10.2 in patients with schizophrenia convicted of serious offences | | | | | | | |
| 1 | Serious | N/A | None | None | N/A | 2.34  (1.60 - 3.41) | VERY LOW |
| Alcohol dependence F10.2 in patients with a psychotic disorder | | | | | | | |
| 1 | Very Serious | N/A | None | Very Serious | N/A | 18.99  (11.86 - 28.99) | VERY LOW |
| Alcohol dependence F10.2 in patients with schizophrenia admitted with parasuicide | | | | | | | |
| 1 | Serious | N/A | None | None | N/A | 1.46  (0.62 - 3.37) | VERY LOW |
| Alcohol dependence F10.2 in patients with serious mental illness | | | | | | | |
| 2 | Serious | N/A | None | None | N/A | 16.76  (13.10 - 20.76) | VERY LOW |
| Alcohol dependence F10.2 in patients detained under section 5(2) of the Mental Health Act | | | | | | | |
| 1 | Serious | N/A | None | Serious | N/A | 3.28  (0.90 - 11.19) | VERY LOW |
| Alcohol dependence F10.2 in patients with personality disorders convicted of serious offences | | | | | | | |
| 1 | Serious | N/A | None | None | N/A | 6.44  (5.00 - 8.25) | VERY LOW |
| Alcohol dependence F10.2 in patients with deliberate self-harm | | | | | | | |
| 3 | Serious | Serious | None | None | N/A | 11.17  (8.35 - 14.32) | VERY LOW |
| Alcohol dependence F10.2 in patients with hepatitis C virus related decompensated cirrhosis | | | | | | | |
| 1 | Serious | N/A | None | None | N/A | 50.98  (48.18 - 53.78) | VERY LOW |
| Alcohol dependence F10.2 in patients with self-poisoning | | | | | | | |
| 1 | Very Serious | N/A | None | None | N/A | 6.12  (4.88 - 7.65) | VERY LOW |
| Alcohol dependence F10.2 in patients unless they were admitted with an acute general surgical condition, a social problem, an overdose, or mental illness | | | | | | | |
| 1 | Serious | N/A | None | Serious | N/A | 16.19  (10.36 - 24.41) | VERY LOW |
| Alcohol dependence F10.2 in patients whom were poisoned by lysergide | | | | | | | |
| 1 | Serious | N/A | None | Serious | N/A | 5.00  (1.71 - 13.70) | VERY LOW |
| Alcohol dependence F10.2 in patients with paracetamol overdose | | | | | | | |
| 1 | Serious | N/A | None | None | N/A | 9.95  (6.53 - 14.87) | VERY LOW |
| Alcohol dependence F10.2 in patients with burns | | | | | | | |
| 1 | Serious | N/A | None | None | N/A | 12.30  (10.62 - 14.20) | VERY LOW |
| Alcohol withdrawal state F10.3 in patients referred to the alcohol liaison nurse | | | | | | | |
| 1 | None | N/A | None | None | N/A | 9.33  (5.98 - 14.26) | LOW |
| Alcohol induced psychotic disorder F10.5 in patients with deliberate self-harm | | | | | | | |
| 1 | None | N/A | None | None | N/A | 8.31  (5.81 - 11.75) | LOW |
| Alcohol induced amnestic disorder F10.6 in patients referred to the alcohol liaison nurse | | | | | | | |
| 1 | None | N/A | None | None | N/A | 0.52  (0.09 - 2.88) | LOW |
| Alcohol induced amnestic disorder F10.6 in patients with acquired brain injury | | | | | | | |
| 1 | Serious | N/A | None | Serious | N/A | 2.33  (0.41 - 12.06) | VERY LOW |
| Poisoning by and exposure to alcohol, undetermined intent Y15 in patients with a diagnosis of poisoning | | | | | | | |
| 1 | Serious | N/A | None | None | N/A | 21.34  (17.68 - 25.53) | VERY LOW |
| Alcoholic hepatitis K70.1 in patients with decompensated cirrhosis | | | | | | | |
| 2 | Serious | N/A | None | None | N/A | 9.93  (7.67 - 12.44) | VERY LOW |
| Alcoholic hepatitis K70.1 in patients admitted to a liver transplant unit | | | | | | | |
| 1 | Serious | N/A | None | None | N/A | 0.95  (0.50 - 1.79) | VERY LOW |
| Alcoholic fibrosis and sclerosis of liver K70.2 in patients with Child’s Class C liver disease with acute variceal bleeding | | | | | | | |
| 1 | Serious | N/A | None | Serious | N/A | 51.96  (42.37 - 61.41) | VERY LOW |
| Alcoholic cirrhosis of liver K70.3 in patients with decompensated cirrhosis | | | | | | | |
| 5 | Serious | Very Serious | None | Very Serious | N/A | 74.39  (52.82 - 91.16) | VERY LOW |
| Alcoholic liver disease, unspecified K70.9 in patients with chronic liver disease | | | | | | | |
| 3 | Serious | Very Serious | None | Very Serious | N/A | 52.70  (29.55 - 75.25) | VERY LOW |
| Alcoholic liver disease, unspecified K70.9 in patients who received liver transplants | | | | | | | |
| 1 | None | N/A | None | None | N/A | 17.38  (14.62 -20.55) | VERY LOW |
| Alcoholic liver disease, unspecified K70.9 in patients with spontaneous bacterial peritonitis in those admitted with ascites | | | | | | | |
| 1 | Very Serious | N/A | None | Very Serious | N/A | 65.38  (46.22 - 80.59) | VERY LOW |
| Alcoholic liver disease, unspecified K70.9 in patients referred to the alcohol liaison nurse | | | | | | | |
| 1 | None | N/A | None | Serious | N/A | 10.88  (7.23 - 16.06) | VERY LOW |
| Alcoholic liver disease, unspecified K70.9 in patients admitted for a liver transplant assessment | | | | | | | |
| 1 | Serious | N/A | None | Serious | N/A | 33.03  (24.91 - 42.30) | VERY LOW |
| Alcoholic liver disease, unspecified K70.9 in patients with a history of variceal bleeding | | | | | | | |
| 1 | Very Serious | N/A | None | Very Serious | N/A | 56.10  (41.04 - 70.11) | VERY LOW |
| Alcoholic liver disease, unspecified K70.9 in patients with severe upper gastrointestinal variceal bleeding | | | | | | | |
| 1 | None | N/A | None | Serious | N/A | 52.87  (45.08 - 60.51) | VERY LOW |
| Alcohol-induced acute pancreatitis K85.2 in patients with acute pancreatitis | | | | | | | |
| 7 | Serious | Very Serious | None | Serious | N/A | 23.55  (17.39 - 30.32) | VERY LOW |
| Alcohol-induced acute pancreatitis K85.2 in patients with exocrine pancreatic disease | | | | | | | |
| 1 | None | N/A | None | Serious | N/A | 6.72  (3.45 - 12.71) | VERY LOW |
| Alcohol-induced chronic pancreatitis K86.0 in patients with chronic pancreatitis | | | | | | | |
| 1 | Very Serious | N/A | None | Very Serious | N/A | 20.37  (11.77 - 32.90) | VERY LOW |
| Alcohol-induced chronic pancreatitis K86.0 in patients with exocrine pancreatic disease | | | | | | | |
| 1 | None | N/A | None | Serious | N/A | 14.29  (9.11 - 21.69) | VERY LOW |
| Alcoholic gastritis K29.2 in patients who are homeless | | | | | | | |
| 1 | Serious | N/A | None | None | N/A | 0.91  (0.16 - 4.97) | VERY LOW |

**Figure S9: Forest plots of meta-analysis for pooled prevalence of wholly attributable alcohol conditions in patients with specific health disorders in the UK hospital system**

**Table S8: Pooled prevalence for wholly attributable alcohol conditions in patients within a specific medical speciality in the UK hospital system**

| Patients within a specific medical specialty | Specialty | Number of Prevalence Estimates (n) | Prevalence % (95%CI) |
| --- | --- | --- | --- |
| **Mental and behavioural disorders due to use of alcohol (F10.x)** |  | | |
| Alcohol Intoxication F10.0 | Psychogeriatrics | 1 | 2.01 (0.69 - 5.75)* |
| Harmful use of alcohol F10.1 | Psychogeriatrics | 1 | 0.67 (0.12 - 3.70)* |
|  | High-security | 2 | 7.19 (6.03 - 8.43) |
|  | Orthopaedics | 1 | 33.88 (29.23 - 38.85) |
| Alcohol dependence F10.2 | Psychogeriatrics | 1 | 0.67 (0.12 - 3.70)* |
|  | Liaison Psychiatry | 1 | 13.83 (8.26 - 22.24) |
| Alcohol withdrawal state with delirium F10.4 | Psychogeriatrics | 1 | 0.67 (0.12 - 3.70)* |
| Alcohol induced psychotic disorder F10.5 | Psychogeriatrics | 1 | 1.34 (0.37 - 4.76)* |
| **Liver disorders due to alcohol K70.x** |  | | |
| Alcoholic cirrhosis of liver K70.3 | Hepatology | 1 | 67.68 (60.88 - 73.80) |
| Alcoholic liver disease, unspecified K70.9 | Gastroenterology | 1 | 14.70 (12.93 - 16.68) |

^All estimates were deemed^ *^very low^* ^quality according to GRADE unless marked by * which indicates^ *^low^* ^quality^

**Table S9: GRADE clinical evidence profile for wholly attributable alcohol conditions in patients within a specific medical speciality in the UK hospital system**

| **Quality Assessment** | | | | | | **Prevalence %  (95% CI)** | **Quality** |
| --- | --- | --- | --- | --- | --- | --- | --- |
| **No of prevalence estimates** | **Risk of bias** | **Inconsistency** | **Indirectness** | **Imprecision** | **Publication Bias** |  |  |
| Alcohol Intoxication F10.0 in psychogeriatric patients | | | | | | | |
| 1 | None | N/A | None | None | N/A | 2.01  (0.69 - 5.75) | LOW |
| Harmful use of alcohol F10.1 in psychogeriatric patients | | | | | | | |
| 1 | None | N/A | None | None | N/A | 0.67  (0.12 - 3.70) | LOW |
| Harmful use of alcohol F10.1 in high security patients | | | | | | | |
| 2 | Serious | N/A | None | None | N/A | 7.19  (6.03 - 8.43) | VERY LOW |
| Harmful use of alcohol F10.1 in orthopaedic patients | | | | | | | |
| 1 | Very Serious | N/A | None | None | N/A | 33.88  (29.23 - 38.85) | VERY LOW |
| Alcohol dependence F10.2 in psychogeriatric patients | | | | | | | |
| 1 | None | N/A | None | None | N/A | 0.67  (0.12 - 3.70) | LOW |
| Alcohol dependence F10.2 in liaison psychiatry patients | | | | | | | |
| 1 | None | N/A | None | Serious | N/A | 13.83  (8.26 - 22.24) | VERY LOW |
| Alcohol withdrawal state with delirium F10.4 in psychogeriatric patients | | | | | | | |
| 1 | None | N/A | None | None | N/A | 0.67  (0.12 - 3.70) | LOW |
| Alcohol induced psychotic disorder F10.5 in psychogeriatric patients | | | | | | | |
| 1 | None | N/A | None | None | N/A | 1.34  (0.37 - 4.76) | LOW |
| Alcoholic cirrhosis of liver K70.3 in hepatology patients | | | | | | | |
| 1 | Serious | N/A | None | Serious | N/A | 67.68  (60.88 - 73.80) | VERY LOW |
| Alcoholic liver disease, unspecified K70.9 in gastroenterology patients | | | | | | | |
| 1 | Serious | N/A | None | None | N/A | 14.70  (12.93 - 16.68) | VERY LOW |

**Figure S10: Forest plots of meta-analysis for pooled prevalence of wholly attributable alcohol conditions in patients within specific medical specialties in the UK hospital system**

**Figure S11: Bubble plots to demonstrate the relationship of mean age to prevalence of wholly attributable alcohol conditions in non-selective patients in the UK hospital system adjusted for setting**

**Figure S12: Funnel plot of prevalence estimates for wholly attributable alcohol conditions in non-selective patients in the UK hospital reported by setting**

**Table S10: Preferred Reporting Items for Systematic Reviews and Meta-Analyses (PRISMA) Checklist**

| **Section/topic** | **#** | **Checklist item** | **Reported on page #** |  |
| --- | --- | --- | --- | --- |
| **TITLE** | | |  |  |
| Title | 1 | Identify the report as a systematic review, meta-analysis, or both. | 1 |  |
| **ABSTRACT** | | |  |  |
| Structured summary | 2 | Provide a structured summary including, as applicable: background; objectives; data sources; study eligibility criteria, participants, and interventions; study appraisal and synthesis methods; results; limitations; conclusions and implications of key findings; systematic review registration number. | 3 |  |
| **INTRODUCTION** | | |  |  |
| Rationale | 3 | Describe the rationale for the review in the context of what is already known. | 5-6 |  |
| Objectives | 4 | Provide an explicit statement of questions being addressed with reference to participants, interventions, comparisons, outcomes, and study design (PICOS). | 6 |  |
| **METHODS** | | |  |  |
| Protocol and registration | 5 | Indicate if a review protocol exists, if and where it can be accessed (e.g., Web address), and, if available, provide registration information including registration number. | 6; OSM |  |
| Eligibility criteria | 6 | Specify study characteristics (e.g., PICOS, length of follow-up) and report characteristics (e.g., years considered, language, publication status) used as criteria for eligibility, giving rationale. | 6-7 |  |
| Information sources | 7 | Describe all information sources (e.g., databases with dates of coverage, contact with study authors to identify additional studies) in the search and date last searched. | 6 |  |
| Search | 8 | Present full electronic search strategy for at least one database, including any limits used, such that it could be repeated. | 6; OSM |  |
| Study selection | 9 | State the process for selecting studies (i.e., screening, eligibility, included in systematic review, and, if applicable, included in the meta-analysis). | 6-7 |  |
| Data collection process | 10 | Describe method of data extraction from reports (e.g., piloted forms, independently, in duplicate) and any processes for obtaining and confirming data from investigators. | 7-8; OSM |  |
| Data items | 11 | List and define all variables for which data were sought (e.g., PICOS, funding sources) and any assumptions and simplifications made. | 7-8 |  |
| Risk of bias in individual studies | 12 | Describe methods used for assessing risk of bias of individual studies (including specification of whether this was done at the study or outcome level), and how this information is to be used in any data synthesis. | 8-9 |  |
| Summary measures | 13 | State the principal summary measures (e.g., risk ratio, difference in means). | 7 |  |
| Synthesis of results | 14 | Describe the methods of handling data and combining results of studies, if done, including measures of consistency (e.g., I^2^) for each meta-analysis. | 9-11 |  |
| Risk of bias across studies | 15 | Specify any assessment of risk of bias that may affect the cumulative evidence (e.g., publication bias, selective reporting within studies). | 8-11 |  |
| Additional analyses | 16 | Describe methods of additional analyses (e.g., sensitivity or subgroup analyses, meta-regression), if done, indicating which were pre-specified. | 10-11 |  |
| **RESULTS** | | | |  |
| Study selection | | 17 | Give numbers of studies screened, assessed for eligibility, and included in the review, with reasons for exclusions at each stage, ideally with a flow diagram. | 12 |
| Study characteristics | | 18 | For each study, present characteristics for which data were extracted (e.g., study size, PICOS, follow-up period) and provide the citations. | Table One |
| Risk of bias within studies | | 19 | Present data on risk of bias of each study and, if available, any outcome level assessment (see item 12). | Table One; OSM |
| Results of individual studies | | 20 | For all outcomes considered (benefits or harms), present, for each study: (a) simple summary data for each intervention group (b) effect estimates and confidence intervals, ideally with a forest plot. | Figure three and four; OSM |
| Synthesis of results | | 21 | Present results of each meta-analysis done, including confidence intervals and measures of consistency. | 12-14Figure three and four; OSM |
| Risk of bias across studies | | 22 | Present results of any assessment of risk of bias across studies (see Item 15). | 12-14; OSM |
| Additional analysis | | 23 | Give results of additional analyses, if done (e.g., sensitivity or subgroup analyses, meta-regression [see Item 16]). | 14-15 |
| **DISCUSSION** | | | |  |
| Summary of evidence | | 24 | Summarize the main findings including the strength of evidence for each main outcome; consider their relevance to key groups (e.g., healthcare providers, users, and policy makers). | 16 |
| Limitations | | 25 | Discuss limitations at study and outcome level (e.g., risk of bias), and at review-level (e.g., incomplete retrieval of identified research, reporting bias). | 17-18 |
| Conclusions | | 26 | Provide a general interpretation of the results in the context of other evidence, and implications for future research. | 18-20 |
| **FUNDING** | | | |  |
| Funding | | 27 | Describe sources of funding for the systematic review and other support (e.g., supply of data); role of funders for the systematic review. | 2 |

**Table S11: Meta-analysis of Observational Studies in Epidemiology (MOOSE) checklist**

| **Item No** | **Recommendation** | **Reported on Page No** |
| --- | --- | --- |
| Reporting of background should include | | |
| 1 | Problem definition | 6 |
| 2 | Hypothesis statement | 6 |
| 3 | Description of study outcome(s) | 7 |
| 4 | Type of exposure or intervention used | 7 |
| 5 | Type of study designs used | 6-7 |
| 6 | Study population | 6-7 |
| Reporting of search strategy should include | | |
| 7 | Qualifications of searchers (eg, librarians and investigators) | 6 |
| 8 | Search strategy, including time period included in the synthesis and key words | 6; OSM |
| 9 | Effort to include all available studies, including contact with authors | 6-7 |
| 10 | Databases and registries searched | 6 |
| 11 | Search software used, name and version, including special features used (eg, explosion) | 6; OSM |
| 12 | Use of hand searching (eg, reference lists of obtained articles) | 6-7 |
| 13 | List of citations located and those excluded, including justification | 12; OSM |
| 14 | Method of addressing articles published in languages other than English | 6; 17 |
| 15 | Method of handling abstracts and unpublished studies | 7 |
| 16 | Description of any contact with authors | 7 |
| Reporting of methods should include | | |
| 17 | Description of relevance or appropriateness of studies assembled for assessing the hypothesis to be tested | 6-7 |
| 18 | Rationale for the selection and coding of data (eg, sound clinical principles or convenience) | 7-8 |
| 19 | Documentation of how data were classified and coded (eg, multiple raters, blinding and interrater reliability) | 7-8 |
| 20 | Assessment of confounding (eg, comparability of cases and controls in studies where appropriate) | N/A |
| 21 | Assessment of study quality, including blinding of quality assessors, stratification or regression on possible predictors of study results | 8-9 |
| 22 | Assessment of heterogeneity | 10-11 |
| 23 | Description of statistical methods (eg, complete description of fixed or random effects models, justification of whether the chosen models account for predictors of study results, dose-response models, or cumulative meta-analysis) in sufficient detail to be replicated | 10-12 |
| 24 | Provision of appropriate tables and graphics | 33-51 |
| Reporting of results should include | | |
| 25 | Graphic summarizing individual study estimates and overall estimate | 50-51; OSM |
| 26 | Table giving descriptive information for each study included | 45; OSM |
| 27 | Results of sensitivity testing (eg, subgroup analysis) | 14-15; 46-47 |
| 28 | Indication of statistical uncertainty of findings | Throughout; 14-15 |

**References:**

1. Higgins JPT, Green S (editors). Cochrane Handbook for Systematic Reviews of Interventions Version 5.1.0 [updated March 2011]. The Cochrane Collaboration, 2011. Available from <http://handbook.cochrane.org>.

2. Egger M., Smith G. D., Schneider M., Minder C. Bias in meta-analysis detected by a simple, graphical test, BMJ 1997: 315: 629.

3. Bradbury A., Robertson C. Prospective audit of the pattern, severity and circumstances of injury sustained by vehicle occupants as a result of road traffic accidents, Archives of emergency medicine 1993: 10: 15-23.

4. Holt S., Stewart I. C., Dixon J. M., Elton R. A., Taylor T. V., Little K. Alcohol and the emergency service patient, British Medical Journal 1980: 281: 638-640.

5. Jain T., Long C., Rashid A., Denney G., Maconochie I., Grey J. et al. The impact of alcohol on conscious level in children and young people, Archives of Disease in Childhood 2012: 97: A61.

6. Johnson I., Nowers M. P., West S. H. Alcohol problems in the elderly: a survey of psychogeriatric admissions, International journal of geriatric psychiatry 2001: 16: 235-236.

7. Locker T. E., Baston S., Mason S. M., Nicholl J. Defining frequent use of an urban emergency department, Emergency medicine journal : EMJ 2007: 24: 398-401.

8. Lockhart S. P., BSc Y. H. C., Straffen A. M., Pang K. K., McLoughlin J., Baron J. H. Detecting Alcohol Consumption as a Cause of Emergency General Medical Admissions, Journal of the Royal Society of Medicine 1986: 79: 132-136.

9. Mehta M. M., Moriarty K. J., Proctor D., Bird M., Darling W. Alcohol misuse in older people: Heavy consumption and protean presentations, Journal of Epidemiology and Community Health 2006: 60: 1048-1052.

10. Rainer T. H., Swann I. J., Crawford R. Critical analysis of an accident and emergency ward, Journal of Accident & Emergency Medicine 1996: 13: 325-329.

11. Trevett A. J., Currie N. M., MacConnell T. J. Alcohol intoxication and alcoholism in acute male medical admissions, Scottish Medical Journal 1990: 35: 134-135.

12. Vardy J., Keliher T., Fisher J., Ritchie F., Bell C., Chekroud M. et al. Quantifying alcohol-related emergency admissions in a UK tertiary referral hospital: a cross-sectional study of chronic alcohol dependency and acute alcohol intoxication, BMJ Open 2016: 6: e010005.

13. Wilkinson G., Bacon N. A. A clinical and epidemiological survey of parasuicide and suicide in Edinburgh schizophrenics, Psychological Medicine 1984: 14: 899-912.

14. Zisman S., O'Brien A. A retrospective cohort study describing six months of admissions under Section 136 of the Mental Health Act; the problem of alcohol misuse, Medicine, science, and the law 2015: 55: 216-222.

15. Ball A. J., Leaning D., Elphick D. A. Improving the acute medical take: The identification of hazardous alcohol consumption, Gut 2011: 60: A49.

16. Barnaby B., Drummond C., McCloud A., Burns T., Omu N. Substance misuse in psychiatric inpatients: Comparison of a screening questionnaire survey with case notes, BMJ: British Medical Journal 2003: 327: 783-784.

17. Barr W., Leitner M., Thomas J. Self-harm patients who take early discharge from the accident and emergency department: how do they differ from those who stay?, Accident and emergency nursing 2004: 12: 108-113.

18. Burke A. W. Attempted suicide among Asian immigrants in Birmingham, British Journal of Psychiatry 1976: 128: 528-533.

19. Cameron A., Morris J. M., Forrest E. H. The prevalence of alcohol misuse among acute admissions: Current experience and historical camparisons, Scottish Medical Journal 2006: 51: 21-23.

20. Canning U. P., Kennell-Webb S. A., Marshall E. J., Wessely S. C., Peters T. J. Substance misuse in acute general medical admissions, Qjm 1999: 92: 319-326.

21. Chalmers J. D., Singanayagam A., Murray M. P., Scally C., Fawzi A., Hill A. T. Risk factors for complicated parapneumonic effusion and empyema on presentation to hospital with community-acquired pneumonia, Thorax 2009: 64: 592-597.

22. Cherry R. Screening for alcohol misuse disorders in our emergency Departments, Ulster Medical Journal 2016: 85 (3): 214.

23. Chick J., Rund D., Gilbert M. A. Orthopaedic trauma in men: the relative risk among drinkers and the prevalence of problem drinking in male orthopaedic admissions, Annals of the Royal College of Surgeons of England 1991: 73: 311.

24. Craig D. G., Bates C. M., Davidson J. S., Martin K. G., Hayes P. C., Simpson K. J. Overdose pattern and outcome in paracetamol-induced acute severe hepatotoxicity, British Journal of Clinical Pharmacology 2011: 71: 273-282.

25. Dolman J. M., Hawkes N. D. Combining the audit questionnaire and biochemical markers to assess alcohol use and risk of alcohol withdrawal in medical inpatients, Alcohol and alcoholism (Oxford, Oxfordshire) 2005: 40: 515-519.

26. Donoghue K., Rose H., Boniface S., Deluca P., Coulton S., Alam M. F. et al. Alcohol Consumption, Early-Onset Drinking, and Health-Related Consequences in Adolescents Presenting at Emergency Departments in England, Journal of Adolescent Health 2017: 60: 438-446.

27. Drummond C., Deluca P., Coulton S., Bland M., Cassidy P., Crawford M. et al. The effectiveness of alcohol screening and brief intervention in emergency departments: A multicentre pragmatic cluster randomized controlled trial, PLoS ONE 2014: 9 (6) (no pagination).

28. Dunn L., Henry J., Beard D. Social deprivation and adult head injury: A national study, Journal of Neurology Neurosurgery and Psychiatry 2003: 74: 1060-1064.

29. Gwaspari M., Hochhauser S., Bruce M. Unmet needs and antisocial personality disorder among Black African and Caribbean service users with severe mental illness, Ethnicity and Inequalities in Health and Social Care 2011: 4: 38-48.

30. Hawton K., Fagg J., Platt S., Hawkins M. Factors associated with suicide after parasuicide in young people, BMJ (Clinical research ed) 1993: 306: 1641-1644.

31. Hawton K., Harriss L. Deliberate self-harm by under-15-year-olds: Characteristics, trends and outcome, Journal of Child Psychology and Psychiatry 2008: 49: 441-448.

32. Hodgins S., Alderton J., Cree A., Aboud A., Mak T. Aggressive behaviour, victimisation and crime among severely mentally ill patients requiring hospitalisation, The British Journal of Psychiatry 2007: 191: 343-350.

33. Howell A., Parker S., Tsitskaris K., Oddy M. J. The burden of bone, native joint and soft tissue infections on orthopaedic emergency referrals in a city hospital, Annals of the Royal College of Surgeons of England 2016: 98: 34-39.

34. Jarman C. M., Kellett J. M. Alcoholism in the general hospital, British Medical Journal 1979: 2: 469-472.

35. Edwards, G, Chandler, J, and Hensman, C,.1972, suppl No 6, pp 94, 120., Quarterly Journal of Studies

on Alcohol 1972: suppl No 6: 120.

36. Knightly R., Tadros G., Sharma J., Duffield P., Carnall E., Fisher J. et al. Alcohol screening for older adults in an acute general hospital: FAST v. MAST-G assessments, BJPsych Bulletin 2016: 40: 72-76.

37. Kouimtsidis C., Reynolds M., Hunt M., Lind J., Beckett J., Drummond C. et al. Substance use in the general hospital, Addictive Behaviors 2003: 28: 483-499.

38. Lennox I. M., Tait C. M. Blood alcohol levels in female acute medical admissions, Health bulletin 1979: 37: 127-129.

39. Lockhart S. P., Baron J. H. Changing ethnic and social characteristics of patients admitted for self-poisoning in West London during 1971/2 and 1983/4, Journal of the Royal Society of Medicine 1987: 80: 145-148.

40. Lumsden J., Chesterman L. P., Hill G. M. Neuropsychiatric indices in a high security admission sample I: Estimating the prevalence, Criminal Behaviour and Mental Health 1998: 8: 285-310.

41. Luttrell S., Watkin V., Livingston G., Walker Z., D'Ath P., Patel P. et al. Screening for alcohol misuse in older people, International Journal of Geriatric Psychiatry 1997: 12: 1151-1154.

42. MacKenzie D., Langa A., Brown T. M. Identifying hazardous or harmful alcohol use in medical admissions: a comparison of audit, cage and brief mast, Alcohol and alcoholism (Oxford, Oxfordshire) 1996: 31: 591-599.

43. Mangion D. M., Platt J. S., Syam V. Alcohol and acute medical admission of elderly people, Age & Ageing 1992: 21: 362-367.

44. Martin B. J., Northcote R. J., Scullion H., Reilly D. Alcohol related morbidity in acute male medical admissions, Health bulletin 1983: 41: 263-267.

45. McCloud A., Barnaby B., Omu N., Drummond C., Aboud A. Relationship between alcohol use disorders and suicidality in a psychiatric population: In-patient prevalence study, The British Journal of Psychiatry 2004: 184: 439-445.

46. McCusker M. T., Basquille J., Khwaja M., Murray-Lyon I. M., Catalan J. Hazardous and harmful drinking: A comparison of the AUDIT and CAGE screening questionnaires, QJM: An International Journal of Medicine 2002: 95: 591-595.

47. McPeake J. M., Shaw M., O'Neill A., Forrest E., Puxty A., Quasim T. et al. Do alcohol use disorders impact on long term outcomes from intensive care?, Critical Care (London, England) 2015: 19: 185.

48. McQueen J. M., Howe T. E., Ballinger C., Godwin J. Effectiveness of Alcohol Brief Intervention in a General Hospital: A Randomized Controlled Trial, Journal of studies on alcohol and drugs 2015: 76: 838-844.

49. Morgan H. G., Burns-Cox C. J., Pocock H., Pottle S. Deliberate self-harm: clinical and socio-economic characteristics of 368 patients, The British journal of psychiatry : the journal of mental science 1975: 127: 564-574.

50. Muzaimi M., Shetty H., Nicholson T. Stroke in young adults in southeast wales, UK: A cohort from a stroke rehabilitation unit, International Journal of Stroke 2010: 2): 299.

51. Ninkovic M. Prevalence of harmful, hazardous or dependent drinking in hospital inpatients on a single day using audit questionnaire, Gut 2012: 2): A404-A405.

52. Oude Voshaar R. C., Cooper J., Murphy E., Steeg S., Kapur N., Purandare N. B. First episode of self-harm in older age: A report from the 10-year prospective manchester self-harm project, Journal of Clinical Psychiatry 2011: 72: 737-743.

53. Poole R., Pearsall A., Ryan T. Delayed discharges in an urban in-patient mental health service in England, The Psychiatric Bulletin 2014: 38: 66-70.

54. Sangha J., Natalwala A., Mann J., Uppal H., Mummadi S. M., Haque A. et al. Co-morbidities and mortality associated with intracranial bleeds and ischaemic stroke, International Journal of Neuroscience 2015: 125: 256-263.

55. Sharkey J., Brennan D., Curran P. The pattern of alcohol consumption of a general hospital population in north Belfast, Alcohol & Alcoholism 1996: 31: 279-285.

56. Sinclair J. M. A., Latifi A. H., Latifi A. W. Co-morbid substance misuse in psychiatric patients: prevalence and association with length of inpatient stay, Journal of psychopharmacology (Oxford, England) 2008: 22: 92-99.

57. Taylor C. L., Kilbane P., Passmore N., Davies R. Prospective study of alcohol-related admissions in an inner-city hospital, Lancet 1986: 2: 265-268.

58. Taylor P. J., Leese M., Williams D., Butwell M., Daly R., Larkin E. Mental disorder and violence. A special (high security) hospital study, British journal of psychiatry 1998: 172: 218-226.

59. Thom B., Herring R., Judd A. Identifying alcohol-related harm in young drinkers: The role of accident and emergency departments, Alcohol and Alcoholism 1999: 34: 910-915.

60. Alavi M., Janjua N., Yu A., Grebely J., Aspinall E., Innes H. et al. Does alcohol dependency explain differences in rates of decompensated cirrhosis among people with a hepatitis C notification? An international comparison, Journal of Hepatology 2016: 1): S462-S463.

61. Barrison I. G., Viola L., Mumford J., Murray R. M., Gordon M., Murray-Lyon I. M. Detecting excessive drinking among admissions to a general hospital, Health trends 1982: 14: 80-83.

62. Ben-Shlomo Y., Markowe H., Shipley M., Marmot M. G. Stroke risk from alcohol consumption using different control groups, Stroke 1992: 23: 1093-1098.

63. Bernadt M. W., Murray R. M. Psychiatric disorder, drinking and alcoholism: What are the links?, The British Journal of Psychiatry 1986: 148: 393-400.

64. Bruce M., Cobb D., Clisby H., Ndegwa D., Hodgins S. Violence and crime among male inpatients with severe mental illness: Attempting to explain ethnic differences, Social Psychiatry and Psychiatric Epidemiology 2014: 49: 549-558.

65. Carney M. W., Sheffield B. F. Alcoholism diagnosis and Celtic names, Irish Journal of Psychological Medicine 1995: 12: 95-100.

66. Corbett C., Duggan, Larkin. Substance misuse and violence: a comparison of special hospital inpatients diagnosed with either schizophrenia or personality disorder, Criminal Behaviour and Mental Health 1998: 8: 311-321.

67. Feldman E., Mayou R., Hawton K., Ardern M., Smith E. B. O. Psychiatric Disorder in Medical In-patients, QJM: An International Journal of Medicine 1987: 63: 405-412.

68. Forrest J. A., Tarala R. A. 60 hospital admissions due to reactions to lysergide (L.S.D.), Lancet (London, England) 1973: 2: 1310-1313.

69. Franklin R. A. One hundred doctors at The Retreat. A contribution to the subject of mental disorder in the medical profession, The British journal of psychiatry : the journal of mental science 1977: 131: 11-14.

70. Glass I. B., Jackson P. Maudsley Hospital Survey: Prevalence of alcohol problems and other psychiatric disorders in a hospital population, British Journal of Addiction 1988: 83: 1105-1111.

71. Hall A. D., Puri B. K., Stewart T., Grahame P. S. Doctors' holding power in practice: Section 5 (2) of the Mental Health Act 1983, Medicine, Science and the Law 1995: 35: 231-236.

72. Hamlyn A. N., Douglas A. P., James O. The spectrum of paracetamol (acetaminophen) overdose: Clinical and epidemiological studies, Postgraduate Medical Journal 1978: 54: 400-404.

73. Herzberg J. L. No fixed abode: A comparison of men and women admitted to an East London psychiatric hospital, The British Journal of Psychiatry 1987: 150: 621-627.

74. Holmes W. J. M., Hold P., James M. I. The increasing trend in alcohol-related burns: it's impact on a tertiary burn centre, Burns : journal of the International Society for Burn Injuries 2010: 36: 938-943.

75. Kelleher M. J. Reasons for the increase in Irish admission rates for alcoholic disorders, The British journal of addiction to alcohol and other drugs 1975: 70: 175-178.

76. Laugharne R. A., Daniels O. J., Lutchman R. The prevalence of alcohol problems amongst in-patients referred to the liaison psychiatrist, Addiction Research 1997: 5: 379-382.

77. MacIntyre D. Alcohol-related problems among male patients admitted to a general medical ward--their identification and follow up, Health bulletin 1979: 37: 213-217.

78. Maguire G. P., Julier D. L., Hawton K. E., Bancroft J. H. J. Psychiatric Morbidity and Referral on Two General Medical Wards, British Medical Journal 1974: 1: 268-270.

79. Mangan B. G., Patterson D. G. The prevalence of alcohol dependence syndrome in a rural general hospital in Northern Ireland, Irish Journal of Psychological Medicine 1994: 11: 73-75.

80. Merrill J., Owens J. Ethnic differences in self-poisoning: a comparison of Asian and white groups, The British journal of psychiatry : the journal of mental science 1986: 148: 708-712.

81. Merrill J., Milner G., Owens J., Vale A. Alcohol and attempted suicide, British Journal of Addiction 1992: 87: 83-89.

82. Orford J. I. M., Somers M., Daniels V., Kirby B. Drinking amongst medical patients: levels of risk and models of change, British Journal of Addiction 1992: 87: 1691-1702.

83. Peters J., Brooker C., McCabe C., Short N. Problems encountered with opportunistic screening for alcohol‐related problems in patients attending an Accident and Emergency department, Addiction 2002: 93: 589-594.

84. Platt S., Robinson A. Parasuicide and alcohol: a 20 year survey of admissions to a regional poisoning treatment centre, International Journal of Social Psychiatry 1991: 37: 159-172.

85. Saxena P., Shankar J. Contralateral hip fractures - can predisposing factors be determined?, Injury 2000: 31: 421-424.

86. Schoepf D., Heun R. Alcohol dependence and physical comorbidity: Increased prevalence but reduced relevance of individual comorbidities for hospital-based mortality during a 12.5-year observation period in general hospital admissions in urban North-West England, European Psychiatry: the Journal of the Association of European Psychiatrists 2015: 30: 459-468.

87. van der Pol V., Rodgers H., Aitken P., James O., Curless R. Does alcohol contribute to accident and emergency department attendance in elderly people?, Journal of Accident & Emergency Medicine 1996: 13: 258.

88. Husain O. M., Lynas P. S., Totty J. P., Williams K., Waring W. S. Unplanned alcohol withdrawal: a survey of consecutive admissions to an acute medical unit in 2010 and 2011, Qjm 2013: 106: 43-49.

89. Dowey K. E. Alcohol-related attendances at an accident and emergency department, Ulster Medical Journal 1993: 62: 58-62.

90. Ramakrishna V. B. Acquired brain injury and secondary organic mental disorders in a secure neurorehabilitation hospital in England: A prevalence study, Brain Injury 2012: 26 (4-5): 687-688.

91. Clark D., Murray D. B., Ray D. Epidemiology and outcomes of patients admitted to critical care after selfpoisoning, Journal of the Intensive Care Society 2011: 12: 268-273.

92. Lawson G. R., Craft A. W., Jackson R. H. Changing pattern of poisoning in children in Newcastle, 1974-81, British Medical Journal 1983: 287: 15-17.

93. Bretherick A. D., Craig D. G. N., Masterton G., Bates C., Davidson J., Martin K. et al. Acute liver failure in scotland between 1992 and 2009; incidence, aetiology and outcome, Qjm 2011: 104: 945-956.

94. Johnston G. W., Spencer E. F., Mullan F. J. Are child's class C patients with acute variceal bleeding worth treating?, HPB surgery : a world journal of hepatic, pancreatic and biliary surgery 1991: 4: 271.

95. Ahmadnia E., Manneh F., Raveendran K. Outcomes of decompensated chronic liver disease in a UK district general hospital critical care setting, Critical Care 2015: 1): S133.

96. Berry P., Thomson S., Ahmed A., Davies M., Ala A. Levels of care and outcomes in decompensated cirrhosis: A descriptive study, Gut 2012: 61: A188.

97. Cole H. L., Pennycook S., Hayes P. C. The impact of proton pump inhibitor therapy on patients with liver disease, Alimentary Pharmacology & Therapeutics 2016: 44: 1213-1223.

98. Dyson J. K., Rajasekhar P., Wetten A., Ashraf H. H., Ng S., Paremal S. et al. Implementation of a 'care bundle' improves the management of patients admitted to hospital with decompensated cirrhosis, Alimentary pharmacology & therapeutics 2016: 44: 1030-1038.

99. Emerson P., McPeake J., O'Neill A., Gilmour H., Forrest E., Puxty A. et al. The utility of scoring systems in critically ill cirrhotic patients admitted to a general intensive care unit, Journal of Critical Care 2014: 29: 1131.e1131-1131.e1136.

100. Hampshire P. A., Musumba C., Shaw R., Gao W., Richardson P. Long-term outcomes of patients with cirrhosis admitted to a general intensive care unit at a tertiary hospital, Journal of the Intensive Care Society 2014: 1): S31.

101. Lloyd-Evans J., Pembroke T., Godkin A. Long term survival of cirrhotics following ICU admission-a subgroup of patients with 'resilient' cirrhosis, Gut 2015: 1): A258-A259.

102. Musumba C., Shaw R., Gao W., Richardson P., Hampshire P. Long-term outcomes of patients with alcohol-related cirrhosis admitted to a general intensive care unit at a tertiary hospital in the United Kingdom, Journal of Hepatology 2013: 1): S224.

103. Shawcross D. L., Austin M. J., Abeles R. D., McPhail M. J. W., Yeoman A. D., Taylor N. J. et al. The impact of organ dysfunction in cirrhosis: survival at a cost?, Journal of hepatology 2012: 56: 1054-1062.

104. Thomson S. J., Moran C., Cowan M. L., Musa S., Beale R., Treacher D. et al. Outcomes of critically ill patients with cirrhosis admitted to intensive care: An important perspective from the non-transplant setting, Alimentary Pharmacology and Therapeutics 2010: 32: 233-243.

105. Al-Freah M. A., Gera A., Martini S., Shawcross D., Abeles R. D., Taylor N. et al. Factors that influence outcome of patients with severe upper gastrointestinal variceal bleeding. a single centre experience, Gut 2010: 59: A103.

106. Bugeja T., Gelson W. T., Griffiths W. J. H. Spontaneous bacterial peritonitis: Prevalence on admission to a tertiary centre and subsequent outcome, Gut 2012: 2): A415.

107. Burke L., Lane C., Gao-Du Y., Driver R., Corless L. Presence of ACLF is the best predictor of mortality in patients with decompensated chronic liver disease managed in a non-specialist environment, Journal of Hepatology 2017: 66 (1 Supplement 1): S383-S384.

108. Butler S. R., Hislop W. S., Fisher B. M., McPeake J. R. Consultants workload due to alcohol related conditions in acute medical receiving gastroenterology and endocrinology, Scottish medical journal 2001: 46: 104-105.

109. Corbett C., Tripathi D., Murphy N., Olliff S., Mangat K. Improved outcomes following covered TIPSS in patients admitted to intensive care following a variceal bleed: A single centre study, Gut 2012: 2): A213.

110. Davies M. H., Langman M. J. S., Elias E., Neuberger J. M. Liver disease in a district hospital remote from a transplant centre: A study of admissions and deaths, Gut 1992: 33: 1397-1399.

111. Docking R. I., Mackay A., Williams C., Lewsey J., Kinsella J., Booth M. G. Comorbidity and intensive care outcome - A multivariable analysis 2C01, 3C00, Journal of the Intensive Care Society 2014: 15: 205-212.

112. Dsouza S., Garner M., Westbrook R. Prevalence and treatment of reduced BMD and vitamin D levels in patients assessed for liver transplantation, Gut 2015: 64: A467-A468.

113. Hislop W. S., Heading R. C., Caledonian Society of G. Impact of alcohol related disease and inpatient workload of gastroenterologists in Scotland, Scottish Medical Journal 2004: 49: 57-60.

114. Lewis M. B., Howdle P. D. Neurologic complications of liver transplantation in adults, Neurology 2003: 61: 1174-1178.

115. McPhail M. J. W., Parrott F., Wendon J. A., Harrison D. A., Rowan K. A., Bernal W. Incidence and Outcomes for Patients With Cirrhosis Admitted to the United Kingdom Critical Care Units, Critical Care Medicine 2018: 46: 705-712.

116. Welch C., Harrison D., Short A., Rowan K. The increasing burden of alcoholic liver disease on United Kingdom critical care units: secondary analysis of a high quality clinical database, Journal of health services research & policy 2008: 13.

117. Ellis M. P., French J. J., Charnley R. M. Acute pancreatitis and the influence of socioeconomic deprivation, British Journal of Surgery 2009: 96: 74-80.

118. Giggs J. A., Bourke J. B., Katschinski B. The epidemiology of primary acute pancreatitis in Greater Nottingham: 1969-1983, Social Science & Medicine 1988: 26: 79-89.

119. Imrie C. W., Whyte A. S. A prospective study of acute pancreatitis, BJS 1975: 62: 490-494.

120. Lowham A., Lavelle J., Leese T. Mortality from acute pancreatitis. Late septic deaths can be avoided but some early deaths still occur, International journal of pancreatology : official journal of the International Association of Pancreatology 1999: 25: 103-106.

121. O'Reilly D. A., McPherson S. J., Sinclair M. T., Smith N. Lessons from a national audit of acute pancreatitis: A summary of the NCEPOD report 'Treat the Cause', Pancreatology : official journal of the International Association of Pancreatology (IAP) [et al] 2017: 17: 329-333.

122. Pavlidis P., Crichton S., Lemmich Smith J., Morrison D., McKenzie C., Atkinson S. et al. Improved outcome of severe acute pancreatitis in the intensive care unit, Intensive Care Medicine 2012: 1): S188.

123. Read G., Braganza J. M., Howat H. T. Pancreatitis--a retrospective study, Gut 1976: 17: 945-952.

124. Toh S. K. C., Phillips S., Johnson C. D. A prospective audit against national standards of the presentation and management of acute pancreatitis in the south of england, Gut 2000: 46: 239-243.

125. Howat H. T. Chronic pancreatitis: medical aspects, Postgraduate Medical Journal 1968: 44: 733-736.

126. Peters T. J., Martin F., Ward K. Chronic alcoholic skeletal myopathy—Common and reversible, Alcohol 1985: 2: 485-489.
